# HPV-Related Oral Lesions: YouTube Videos Suitability for Preventive Interventions including Mass-Reach Health Communication and Promotion of HPV Vaccination

**DOI:** 10.3390/ijerph20115972

**Published:** 2023-05-27

**Authors:** Federica Di Spirito, Alessandra Amato, Francesco D’Ambrosio, Davide Cannatà, Maria Pia Di Palo, Nicoletta Coppola, Massimo Amato

**Affiliations:** 1 Department of Medicine, Surgery and Dentistry, University of Salerno, 84084 Salerno, Italy; 2 Department of Neuroscience, Reproductive Science and Dentistry, University of Naples Federico II, 80131 Naples, Italy

**Keywords:** human papillomavirus, HPV, prevention, vaccination, education, oral lesions, oral cancer, video, content, YouTube

## Abstract

Given the need to improve patient knowledge about HPV-related oral lesions, awareness of HPV infection prevention measures, and compliance with vaccination, as well as patient demand for free and easy access to well-tailored and time-saving health information, the present cross-sectional study examined the accuracy of relevant YouTube videos and their suitability for mass-reach health communication and HPV vaccination promotion. A video search was performed, using keywords obtained from the Google Trends website, until 9 January 2023. Video selection and data collection were performed by independent, pre-calibrated examiners. Descriptive statistics were performed on videos’ general characteristics, source reliability, popularity, information and quality, content topics, vaccination-encouraging/discouraging messages, and educational value. Pearson’s correlation was calculated between educational value and all parameters. Mann–Whitney U test compared very low/low vs. medium/good/excellent educational value and HPV vaccination-encouraging vs. -discouraging videos. Most of the 97 YouTube videos analyzed were moderately accurate and reliable, 53% had moderate/good/excellent educational value, and 80% encouraged HPV vaccination, making them suitable for mass-reach communication. The limited role of oral healthcare providers in uploading relevant content, with the poor dissemination of information about HPV-related benign and malignant oral lesions, may be expanded by purposefully using YouTube and other mass media to improve patient knowledge of HPV-related oral lesions and promote HPV vaccination, which also underscores its potential beneficial oral effects.

## 1. Introduction

Human papillomavirus (HPV) is the most common sexually transmitted pathogen in the world population [1,2]. HPV can also be transmitted by skin-to-skin or skin-to-mucosa contact [3,4], and causes benign and malignant mucocutaneous lesions in females and males [5].

Approximately 200 viral genotypes, over 40 associated with low-risk HPV and 15 with high-risk HPV [6], have been identified, 25 of which are associated with benign and malignant oral lesions: HPV-1, -2, -3, -4, -6, -7, -10, -11, -13, -16, -45, -18, -31, -32, -33, -35, -40, -45, -52, -55, -57, -58, -59, -69, -72, -73. HPV-6 and HPV-11 are found in squamous cell papillomas, HPV-6 and HPV-11 in condyloma acuminata [7,8], HPV-2, -57, -4, and -40 in verrucae vulgaris [9], and HPV-13 and -32 in focal epithelial hyperplasia [7,8].

HPV-16 and HPV-18 genotypes are the most commonly associated with HPV-related Oral Squamous Cell Carcinoma (OSCC) [10]. Approximately 3% of OSCCs are likely related to the HPV carcinogenic pathway rather than the oncogenic effects of tobacco and alcohol, which are actually recognized as predominant risk factors for oral cancer [7,8]. Nevertheless, more than 80% of oropharyngeal cancers in the United States are estimated to be associated with HPV infection [11].

Moreover, while the early detection of HPV-related cervical cancer can be aided by screening tests, such as the Pap smear and HPV test [12], there are currently no secondary prevention measures for either HVP-related OSCC or oral benign lesions [12]. Consequently, the prevention of oral HPV infection and related lesions mainly relies on primary prevention strategies aimed at infection control through vaccination and education [13,14].

Three types of HPV vaccines are currently administered: Gardisil (Quadrivalent; Merck & Co, Kenilworth, NJ, USA) against HPV-6, -11, -16, and -18 genotypes; Gardisil9 (Nonavalent; Merck & Co, Kenilworth, NJ, USA) against HPV-6, -11, -16, -18, -31, -33, -45, -52, and -58 genotypes; and Cervarix (Bivalent; GSK, Brentford, UK) against HPV-16 and -18 [15]. Over 90% of vaccinated individuals have been shown to develop HPV-16 antibodies (Immunoglobulin G) in oral fluids following vaccine administration [16]. Accordingly, HPV vaccination is also among the measures endorsed to control OSCC risk factors, along with tobacco and alcohol restrictions [11]. In addition, it has been estimated that more than 5000 new cases of oropharyngeal cancer in 50 years and 50,000 cases in 100 years may be prevented by also vaccinating males [17]. The American Cancer Society recommends HPV gender-neutral vaccination between 9 and 12 years of age, regardless of HPV status [18]. However, HPV vaccination has not been officially approved by the Food and Drug Administration for the prevention of oropharyngeal cancer, which indirectly contributes to decreased awareness of the positive association between HPV vaccination and OSCC [19].

Nevertheless, because HPV vaccination provides more effective protection against cancer when given at a younger age [20], pediatricians and pediatric dentists can play a crucial role in educating patients about HPV infection, related lesions, including oral ones, and HPV vaccination [21,22]. Accordingly, although oral and dental healthcare providers have long limited their role to the secondary prevention of HPV-related benign lesions and oral/oropharyngeal cancer through clinical examination [23], the American Dental Association (ADA) and the American Academy of Pediatric Dentistry (AAPD) have recently proposed expanding their role [24] in educating and raising awareness of HPV-related oral lesions and recommending HPV vaccination [25,26].

Patient education regarding HPV transmission routes, virus-related mucocutaneous and oral lesions, and awareness of possible latent or persistent HPV infection, especially if sustained by the so-called high-risk viral genotypes, can help limit viral transmission [8,27,28]. Similarly, sexual education encouraging safe practices, such as condom use and barriers during oro-genital intercourse [14,19,29], and promoting healthy lifestyles, such as by supporting smoking cessation [30], as tobacco smoking is significantly associated with HPV infection [31], are critical prevention strategies [14,29].

Patient education that increases literacy and leads to behavior changes is based on direct communication in clinical environments [32,33], indirect advice in selected settings (i.e., schools), and population-based approaches [34]. Among the latter, mass media campaigns have been shown to positively affect tobacco use, dietary habits, sun protection, and other health-related issues [34]. The proliferation of digital communication in recent decades has further expanded the range of tools for mass-reach health communication, with promising results regarding the provision of trustworthy and truthful information [35].

Social media is considered the easiest and fastest means of mass-reach communication at present [22,36,37]. YouTube, in particular, is a free web platform that allows for users to share personal experiences, obtain clinical information, and upload health-related content, reaching, and thus potentially educating, a large audience [38]. In addition, YouTube, like other social media, has a high persuasive power for the population. From this perspective, preventive health-related topics covered on YouTube may positively impact awareness and attitudes toward preventive measures and proposed interventions for a large number of people who have access to the Internet [39].

The disseminated information should be understandable to everyone, easy to remember [32], and accurate to avoid misinformation. However, users can upload arbitrary videos on YouTube, which may contain unreliable information [40].

Therefore, given the need to improve patients’ knowledge about HPV-related oral lesions, awareness of HPV infection prevention measures, and compliance with vaccination, as well as patients’ demand for easy access to tailored and time-saving health information, the present cross-sectional study investigated the accuracy of YouTube videos about HPV infection, mucocutaneous and oral lesions, and vaccination, as well as their suitability for preventive interventions, including mass-reach health communication and the promotion of HPV vaccination.

## 2. Materials and Methods

### 2.1. Study Design

The study protocol was established before the search began. Computer history and cookies were cleared so as not to affect the results based on previous computer searches. The “Return YouTube Dislike” extension (freely available at https://returnyoutubedislike.com/install, accessed on 9 January 2023) was downloaded and installed to record YouTube video dislikes.

Because the content of all videos contained public data, obtaining approval from the local research ethics committee was unnecessary.

### 2.2. Search Strategy

The Google Trends website (freely available at https://trends.google.com/trends/, accessed on 9 January 2023) was searched to determine the most frequently used search term worldwide in the past five years among “HPV/Human papillomavirus infection”, “HPV/Human papillomavirus lesions”, “HPV/Human papillomavirus vaccine”, and “HPV/Human papillomavirus vaccination”. The relevant term searched most frequently on both Google and YouTube was “HPV vaccine”.

Accordingly, the video search was performed using the keyword “HPV vaccine” by selecting the source “youtube.com” in the “Videos” section of the Google search engine, without upload date restrictions and limiting video duration to 4–20 min.

Of the videos uploaded by 9 January 2023, and sorted by order of appearance, the first 120 were considered because 80–90% of YouTube users only view the first three pages of a search, as previous studies have shown [41,42].

All video source paths (URLs) were saved and recorded on the same day to avoid losing search results, which may change in the following days.

### 2.3. Eligibility Criteria

Three independent investigators (M.P.D.P, D.C., N.C.) eliminated duplicates, and videos retrieved from YouTube (freely available at http://www.youtube.com, accessed on 9 January 2023) that were compliant with the eligibility criteria shown in Table 1 were included in the present analysis.

### 2.4. Data Collection

Three independent and pre-calibrated investigators M.P.D.P, D.C., N.C. extracted, calculated, and collected the following data in a standardized extraction form for each video included in this study:

▪ Characteristics: link, length (minutes), number of views, number of likes, number of dislikes, number of comments, number of subscriptions, time elapsed since upload;
▪ Source: classified as dental care providers, other healthcare providers (any), hospital/university, pharmaceutical industries, commercial, other;
▪ Target audience: classified as laypersons, professionals, both;
▪ Score: Video Power Index (VPI) [36,43], Video Information and Quality (VIQI) [43], Video Content [44], Video Source Reliability [45], Video Educational Value (GQS) [44,46].

In case of disagreement, a fourth examiner was involved (F.D.A.)

### 2.5. Video Power Index

The *Video Power Index*, assessing video popularity, was calculated as follows [36,40,43]:

$$\mathrm{Like}\;\mathrm{ratio}\;=\;\frac{N.\;\mathrm{of}\;\mathrm{likes}\;+\;N.\;\mathrm{of}\;\mathrm{dislikes}}{N.\;\mathrm{of}\;\mathrm{views}}\;\times \;100$$

$$\mathrm{View}\;\mathrm{ratio}\;=\;\frac{N.\;\mathrm{of}\;\mathrm{views}}{N.\;\mathrm{of}\;\mathrm{days}\;\mathrm{since}\;\mathrm{the}\;\mathrm{video}\;\mathrm{was}\;\mathrm{uploaded}}\;\times \;100$$

$$\mathrm{Video}\;\mathrm{Power}\;\mathrm{Index}\;=\;\frac{\mathrm{Like}\;\mathrm{ratio}\;\times \mathrm{View}\;\mathrm{ratio}}{100}$$

### 2.6. Video Information and Quality Assessment

The Video Information and Quality Index (VIQI), with a score from 1 to 20 [46], evaluated the overall quality of the videos.

A 5-point Likert-type scale (1 = low quality to 5 = high quality) was used to assess each of the following parameters: “flow of information; accuracy of information; quality (use of photographs, animations, reporting by audience members, video titles and summaries); sensitivity (level of consistency between video title and content)”.

### 2.7. Video Content Assessment

The content of the videos was evaluated, providing a total content score of 1–23, according to the following topics:

- Content on HPV infection: route of transmission; risk factors; screening; genotypes; oncogenic role.
- Content on HPV-related lesions: skin lesions (benign); mucosal lesions (benign); oral lesions (benign); female genital cancer; male genital cancer; oral cancer (oral squamous cell carcinoma, OSCC).
- Content on HPV vaccine; age; gender; type of vaccines; HPV vaccine safety; HPV vaccination advice (encouraging, discouraging or neutral); face news.
- Content on HPV-related lesions and vaccination epidemiology: vaccination course; projections of female genital cancer; projection of male genital cancer; projections of oral cancer (oral squamous cell carcinoma, OSCC) [47,48,49].

### 2.8. Video Source Reliability

The four benchmark criteria of the Journal of American Medical Association (JAMA), suggested by Silberg et al. [45], were used to assess the reliability of the source of the medical information contained in the videos. The JAMA score ranged from 1 to 4 according to the following criteria:

- “Authorship (authors and contributors, their affiliations, and relevant credentials should be provided)
- Attribution (references and sources for all content should be listed clearly, and all relevant copyright information reported)
- Disclosure (website “ownership” should be prominently and fully disclosed, as should any sponsorship, advertising, underwriting, commercial funding arrangements or support, or potential conflicts of interest)
- Currency (dates that content was posted and updated should be indicated)”.

### 2.9. Video Educational Value

The videos’ educational value was evaluated based on the 5-point Global Quality Scale (GQS) criteria [44,46], as follows:

- “Score 1 = Poor quality; very unlikely to be of any use to patients
- Score 2 = Poor quality but some information present; of very limited use to patients
- Score 3 = Suboptimal flow, some information covered but important topics missing; somewhat useful to patients
- Score 4 = Good quality and flow, most important topics covered; useful to patients
- Score 5 = Excellent quality and flow; highly useful to patients.

Videos rated 3< were classified as having very low/low educational value, and those rated ≥ 3 as having medium/good/excellent educational value”.

### 2.10. Statistical Analysis

The Shapiro–Wilk test was used to calculate the normality of the data distribution. Descriptive statistical analysis was performed for all the included YouTube videos.

The correlation between educational value and video characteristics, popularity, Information and Quality Index (VIQI), content topics and total score, and video source reliability was calculated through Pearson’s correlation test.

Very low/low and medium/good/excellent videos based on their educational value were compared using the Mann–Whitney U-test. A further comparison was similarly performed between HPV vaccination-encouraging and -discouraging YouTube videos.

## 3. Results

### 3.1. YouTube Videos on HPV Infection, Mucocutaneous and Oral Lesions, and Vaccination: Inclusion and Data Collection

A total of 313 videos were retrieved, of which 23 were excluded using the duration filter, eliminating videos of less than 4 min and more than 20 min.

Of the remaining 290 videos, sorted by order of appearance, the first 120 were included.

Twenty-three videos were further excluded, specifically 4 that were not in English and 19 in which HPV-related lesions and vaccination were not the primary content(s).

A total of 97 YouTube videos on HPV met the eligibility criteria and were included in the present study.

The extracted, calculated, and collected data are summarized in Supplementary Table S1.

### 3.2. YouTube Videos on HPV for Mass-Reach Health Communication and Vaccination Promotion: Descriptive Analysis

The mean length of the videos was 7.88 (4.02–17.4). Videos were uploaded between 5 and 4885 days (mean time elapsed since upload 1527.82) before the search, and received a mean number of views of 27,026.6, with a mean number of likes of 375.77 (0–13,002) and a mean number of dislikes of 47.66 (0–2796), providing a mean Video Power Index (VPI) of 48.03.

The mean Video Information and Quality Index VIQI (0–20 score) was 12.

The mean video educational value (0–5 score) was 2.59, meaning that, on average, the videos were classified as being of low to medium educational value.

Descriptive statistics of video length, number of views, likes, dislikes, comments, and subscriptions, the time elapsed since upload, the Video Power Index (VPI), the Video Source Reliability, the Video Information and Quality Index (VIQI), the video content, and the video educational value (GQS) of the YouTube contents on HPV that were analyzed are detailed in Table 2.

The mean Video Source Reliability (0–4 score) was 2.78, and no videos were uploaded by dental care providers. Other healthcare providers, who uploaded about 9% of the videos, were obstetrician–gynecologists (33%), general practitioners (22%), pediatricians (22%), infectious disease specialists (11%), and sexual health physicians (11%).

Most of the videos (58%) were directed to laypersons.

**Figure 1:**
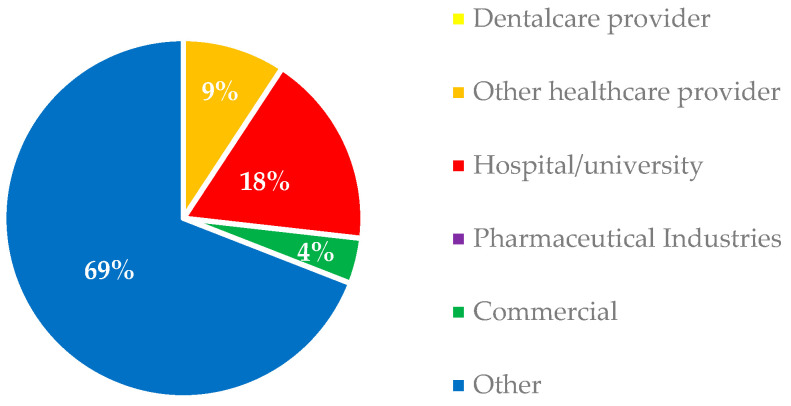
Sources of YouTube videos on HPV infection, mucocutaneous and oral lesions and vaccination.

**Figure 2:**
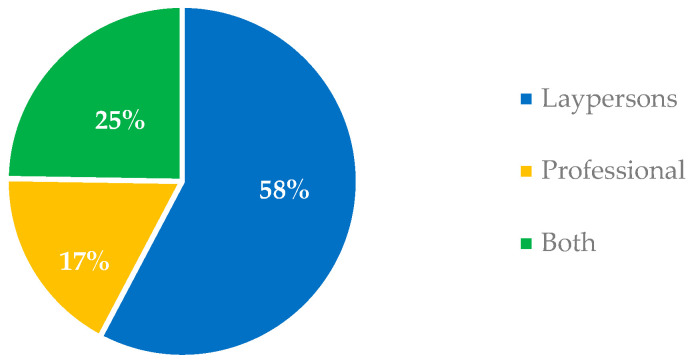
Target audience of YouTube videos on HPV infection, mucocutaneous and oral lesions and vaccination.

The source and target audience of YouTube videos on HPV are shown in Figure 1 and Figure 2.

The mean video total content score (1–23 score) was 9.91, indicating that the videos included covered less than half of the investigated topics.

**Figure 3:**
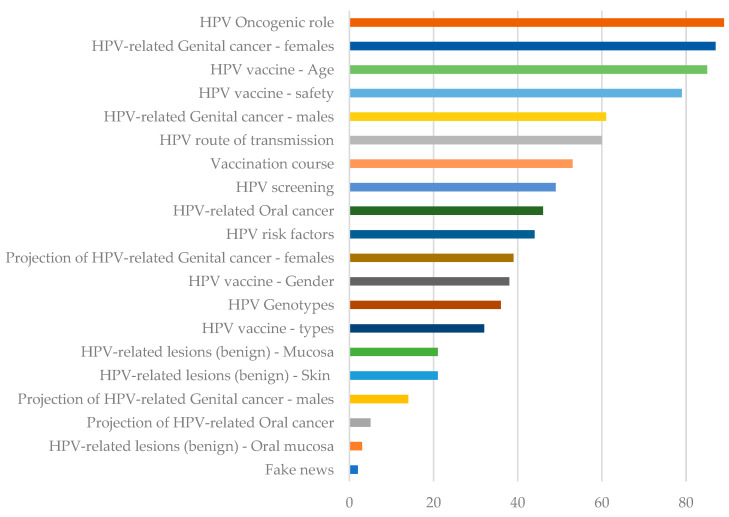
Content topics of YouTube videos on HPV infection, mucocutaneous and oral lesions and vaccination.

The oncogenic role of HPV was the most frequently covered topic (92%), followed by the role of HPV in female genital cancer (90%), the recommended age for HPV vaccination (88%), and the safety of HPV vaccines (81%). HPV-related oral lesions were the least covered topic (3%) (Figure 3).

The educational value of the videos, as assessed by the 5-point Global Quality Scale (GQS), was distributed as follows: 14% of the videos had a very low value, 32% had a low value, while 36%, 15%, and 2% of the analyzed videos were assigned a medium, good, and excellent value, respectively.

### 3.3. YouTube Videos on HPV for Mass-Reach Health Communication and Vaccination Promotion: Correlation between Video Educational Value and Other Parameters

No statistically significant correlation was found between video educational value and any other parameters, except for the video length (Pearson’s r = 0.096, *p*-value = 0.0498), loosely significant, and HPV vaccination content concerning “Gender” (Pearson’s r = −0.341 *, *p*-value = 0.013).

The results of the Pearson correlation test are shown in Table 3.

### 3.4. YouTube Videos on HPV for Mass-Reach Health Communication and Vaccination Promotion: Comparison between Very Low/Low and Medium/Good/Excellent Educational Value Videos

Forty-five (46.39%) YouTube videos on HPV infection, mucocutaneous and oral lesions and vaccination were rated 3< and classified as having very low/low educational value, and the remaining 52 (53.60%) were rated ≥ 3 as having medium/good/excellent educational value, based on the 5-point Global Quality Scale (GQS).

Variables of very low/low and medium/good/excellent regarding the educational value of videos are reported in Table 4.

When comparing YouTube videos on HPV with very low/low and medium/good/excellent educational value, significant differences (*p*-value < 0.05) were found for time elapsed since upload (*p*-value = 0.022), Video Information and Quality Index (VIQI) (*p*-value = 0.015) and “Professionals” audience (*p* value = 0.016). A comparison of variables between videos with very low/low and medium/good/excellent educational value, calculated with the Mann–Whitney U test, is shown in Table 5.

### 3.5. YouTube Videos on HPV for Mass-Reach Health Communication and Vaccination Promotion: Comparison between Vaccination Encouraging and Discouraging Videos

**Figure 4:**
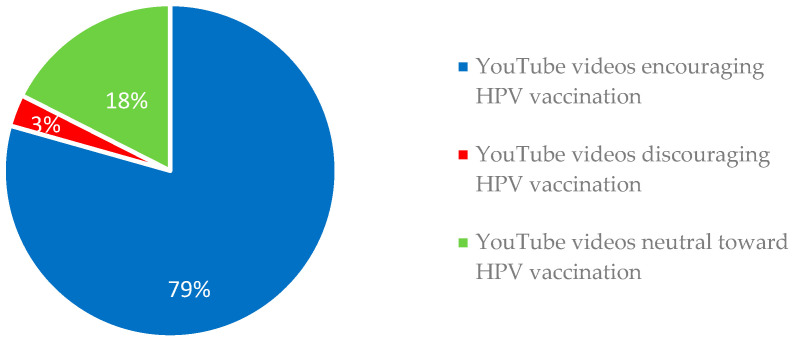
Videos encouraging, discouraging and neutral toward HPV vaccination.

As shown in Figure 4, 79% of the analyzed YouTube videos encouraged HPV vaccination, while 3% were discouraging, and 18% were neutral towards vaccination.

Variables of vaccination-encouraging and -discouraging YouTube videos are reported in Table 6.

When comparing YouTube videos encouraging and discouraging vaccination, a significant difference was found in the number of views (*p*-value = 0.01), the time elapsed since upload (*p*-value = 0.03), Video Power Index (VPI) (*p*-value = 0.014), Video Content score (*p*-value < 0.049) and Video Source Reliability (*p*-value < 0.001). A comparison of variables between vaccination-encouraging and -discouraging videos computed with the Mann–Whitney U test is shown in Table 7.

During synthesis, most of the 97 YouTube videos on HPV infection, mucocutaneous and oral lesions and vaccination that were included in the present study and analyzed were moderately accurate and reliable, with a mean Video Source Reliability (0–4 score) of 2.78. The mean video educational value (0–5 score) was 2.59, meaning that, on average, the videos were classified as being of from low to medium educational value. The mean Video Total Content score (1–23 score) was 9.91, indicating that the videos included covered less than half of the investigated topics, with the oncogenic role of HPV being the most frequently covered topic (92%), followed by the role of HPV in female genital cancer (90%), the recommended age for HPV vaccination (88%), and the safety of HPV vaccines (81%). HPV-related oral lesions were the least covered topic (3%).

No statistically significant correlation was found between video educational value and any other parameters, except for the video length and HPV vaccination content concerning gender.

About half (46.39%) of YouTube videos on HPV infection, mucocutaneous and oral lesions and vaccination were rated 3< and classified as having very low/low educational value, and the remaining 52 (53.60%) were rated ≥ 3 as having medium/good/excellent educational value, based on the 5-point Global Quality Scale (GQS). When comparing YouTube videos on HPV with very low/low and medium/good/excellent educational value, significant differences (*p*-value < 0.05) were found for time elapsed since upload (*p*-value = 0.022), Video Information and Quality Index (VIQI) (*p*-value = 0.015) and “Professionals” audience (*p* value = 0.016).

The majority (79%) of the videos encouraged HPV vaccination, while only 3% were discouraging, and 18% were neutral towards vaccination. When comparing YouTube videos encouraging and discouraging vaccination, a significant difference was found in the number of views (*p*-value = 0.01), time elapsed since upload (*p*-value = 0.03), Video Power Index (VPI) (*p*-value = 0.014), Video Content score (*p*-value < 0.049) and Video Source Reliability (*p*-value < 0.001).

## 4. Discussion

Given the need to improve patient knowledge about HPV-related oral lesions [50], awareness of HPV infection prevention measures, and compliance with vaccination, as well as patient demand for easy access to well-tailored and time-saving health information, the present cross-sectional study examined the accuracy of YouTube videos on HPV infection, oral and mucocutaneous lesions and vaccines, and their suitability for preventive interventions including mass-reach health communication and the promotion of HPV vaccination.

A total of 97 YouTube videos on HPV were included in the analysis. Compared to the 120 videos that were initially found in the search, based on the general likelihood that YouTube users view only the first few pages of results [41], the reduction in the number of analyzed videos was mainly due to the lack of information regarding HPV vaccination among the primary topics divulged.

In addition, restricting the video duration to 4–20 min certainly limited the search results. Nonetheless, given that the suggested optimal video length to maintain viewer attention is between 5–6 and 10 min [51,52], the applied duration filter may have indirectly increased the likelihood that videos were watched from beginning to end, likely making the presented results generalizable, especially in terms of mass-reach effective communication. However, no data on viewing duration could be found on YouTube.

### 4.1. YouTube Videos on HPV Infection, Lesions and Vaccination for Mass-Reach Health Communication: Characteristics, Popularity, Source Reliability, Flow and Accuracy of Information

The number of views of the analyzed videos varied widely, from 5 to 695,711 (Table 2); however, on average, the included videos had a wider distribution compared to previous findings [53]. Instead, the popularity of the videos was consistent with the literature, with an average of approximately 375.77 likes, a mean of approximately 47.66 likes, and a mean Video Power Index (VPI) of 48.03 (Table 2).

Most videos (69%) were uploaded by nonprofit or academic organizations and fell into the “Other” category (Figure 1). Hospitals and universities uploaded 18% of YouTube videos on the HPV, and 9% were produced by health care providers, of whom 33% were gynecologists, 22% were general practitioners, 22% were pediatricians, 11% were infectious disease specialists, and 11% were sexual health physicians (Figure 1). No dental care providers uploaded videos on this topic, suggesting that dentists and dental hygienists still play a minor role in disseminating knowledge about HPV infection and oral lesions and promoting HPV vaccination. In addition, no video produced by the pharmaceutical industry was found on the first pages of the YouTube searches.

The mean Video Source Reliability (0–4 score) was 2.78 (Table 2). The criterion least met by the videos was that of “disclosure”.

More than half of the videos (58%) were directed to laypersons (Figure 1), indicating that most of the YouTube videos on HPV were likely uploaded to share knowledge with nonprofessional audiences, which include the majority of the vaccine’s target audience, such as preschool-aged children, adolescents, young adults, and their parents.

In addition, the mean Video Information and Quality Index (VIQI) (0–20) was 12 (Table 2), indicating that the information flow and accuracy, the consistency between the video title and content, and the overall quality of the video (photos, animations, video headlines, summary, etc.) were generally moderate.

### 4.2. YouTube Videos on HPV Infection, Oral Lesions and Vaccination for Mass-Reach Health Communication: Content Topics

The mean Video Content (1–23) score was 9.91, suggesting that YouTube videos, on average, covered less than half of the examined information about HPV infection, oral and mucocutaneous lesions, and vaccination.

In detail, HPV infection and risk factors, although crucial to primary prevention, were addressed in less than half of the videos.

Distinguishing between HPV genotypes occurred in less than a quarter of the YouTube videos, likely because they were primarily aimed at laypeople whose cultural background may not be sufficient to understand this topic.

Slightly more than 60% of YouTube videos about HPV discussed the route of viral transmission, but they generally described only sexual transmission. However, it should be noted that HPV is primarily transmitted through skin-to-skin or skin-to-mucosa contact [3].

Furthermore, nearly half of the videos provided information on screening methods for HPV, and some emphasized the importance of HPV screening even in individuals who have received an HPV vaccine.

HPV-related benign lesions were a less-discussed topic compared with malignancies. Specifically, benign HPV-related skin lesions and benign mucosal lesions (anogenital warts) were addressed in two of the ten videos. It would be desirable for this last topic to be emphasized more, as previous studies have shown that the association between HPV infection and skin and genital warts is poorly understood in the general population [54].

Moreover, only 3% of YouTube videos addressed benign HPV-related oral lesions (squamous cell papilloma, condyloma acuminatum, verruca vulgaris, and focal epithelial hyperplasia) (Figure 3). This finding highlighted that knowledge of the consequences of HPV infection in the oral cavity is low, suggesting that the role of dental care providers, who are often the first to recognize these lesions, needs to be strengthened to motivate protective measures [55].

Notably, 92% of YouTube videos addressed the oncogenic role of HPV, the most frequently covered topic in YouTube videos about HPV (Figure 3). Indeed, most of the authors of these analyzed videos emphasized that HPV vaccination is not only a vaccine to prevent infection but, more importantly, an effective means of cancer prevention, as HPV genotypes cause nearly 5% of human cancers worldwide [56], contrary to findings from a previous study, conducted about a decade ago [57]. Accordingly, HPV-related cancers were also a very frequently covered topic. Specifically, female genital cancers associated with HPV infection (cervical cancer, vaginal cancer, vulvar cancer, and anal cancer) were discussed in 90% of the included YouTube videos. In contrast, male genital cancers associated with HPV (penile and anal cancers) were discussed in six of ten videos (Figure 3). On the other hand, OSCC and oropharyngeal cancers were discussed in less than half of the videos. This finding contrasts with the rapidly increasing incidence of HPV-positive squamous cell carcinoma of the oral cavity and oropharynx in high-income countries [53].

Regarding information about HPV vaccination, it is worth highlighting that 81% of the YouTube videos guaranteed the safety of vaccine administration, as there is no evidence that the vaccine is associated with serious physical risks or side effects [58].

Age for vaccination was another frequently raised issue, further underpinning that individuals should be vaccinated before they potentially come into contact with the virus [59]. However, the need to improve gender-neutral HPV vaccination did not appear to be fully appreciated, as only 39% of YouTube videos addressed this topic, and a negative correlation was found between video educational value and HPV vaccination content concerning the “Gender” (Pearson’s r = −0.341 *, *p*-value = 0.013).

The included YouTube videos did not contain “Fake News”. This was a promising finding, as inaccurate content could potentially mislead public opinion and negatively impact vaccination programs and other prevention efforts [40].

Finally, epidemiologic information on HPV, such as vaccination progression and projections regarding female and male genital cancers and OSCC, was unfortunately not emphasized, nor was the potentially positive role of HPV vaccination on OSCC prevention.

### 4.3. YouTube Videos on HPV for Mass-Reach Health Communication and Vaccination Promotion: Educational Value

Nearly half (46%) of YouTube video on HPV vaccine were of very low/low educational value (GQS < 3), and just over half (54%) were of medium/good/excellent educational value (GQS ≥ 3); specifically, 36% of the videos had a medium educational value, and 15% and 2% had a good and excellent educational value, respectively. However, the majority of YouTube videos about HPV were rated as poor for patient education.

These findings are consistent with those of YouTube videos on other medical and dental content. For example, studies of the accuracy and quality of YouTube videos in orthopedics [36] and allergology/immunology [60] found that the content is inadequate for educational purposes. Similarly, the information quality of YouTube videos on burning mouth syndrome [61] and endodontic treatment [62] was rated as poor for patient education. In contrast, more than half of the videos on Sjogren’s syndrome [63] and about two-thirds of the YouTube videos on type 2 diabetes [64] were considered very useful for patients.

No significant correlations were found between the video educational value and all other parameters (Table 3).

When comparing the YouTube videos with very low/low and medium/good/excellent educational value, a significant difference was found in Video Information and Quality Index (VIQI) (*p*-value < 0.05), indicating how closely the quality of the information provided is related to its usefulness in improving patient knowledge for educational purposes (Table 5 and Table 6).

### 4.4. YouTube Videos on HPV Infection, Oral Lesions and Vaccination for the Promotion of HPV Vaccination

The HPV vaccine effectively prevents infections caused by oral HPV genotypes 16 and 18 [6]. However, because the vaccine’s efficacy lasts < 10 years [65,66,67], its long-term effectiveness in reducing the incidence and mortality rates associated with HPV-related head and neck cancers is questionable [6]. In the United States, HPV vaccine administration is currently recommended for girls aged 11–12 years, which was extended to 26 years in 2019, according to the Advisory Committee on Immunization Practices (ACIP), and for boys up to 21 years [68]. Nevertheless, HPV vaccination may also benefit individuals at high risk for new viral infection between the ages of 27 and 45 [6,27,69]. However, according to the American Cancer Society (ACS), HPV vaccines should be administered between the ages of 9 and 12 years for optimal efficacy [18], as administration at an older age seems less effective for cancer prevention [18,20,70].

Since HPV vaccine administration has become a burning public issue and the advancement in digital medicine [71], the Internet has become an essential channel for disseminating information on this topic. In particular, organizations, hospitals, universities, professionals, and laypersons uploaded YouTube videos to disseminate knowledge and share their experiences and opinions about HPV vaccination [57,72,73]. In addition, the messages conveyed through YouTube videos is known to have the potential to influence the risk–benefit perception of the vaccine and the vaccination intentions of the audience [74,75]. In this regard, most videos uploaded just prior to the release of HPV vaccines encouraged vaccination [75]. Later, a reversal in social media trends was observed instead, as highlighted by Briones et al. [57] in 2012 and Ekram et al. [76] in 2019, and most videos discouraged HPV vaccination. In the present analysis, nearly 80% of videos endorsed HPV vaccination, while 3% advised against it; the remaining videos were neutral toward such a preventive measure (Figure 4).

When comparing YouTube videos encouraging and discouraging HPV vaccination (Table 7), significant differences were found in the time since upload (*p*-value = 0.03), the number of views (*p*-value = 0.01), and the Video Power Index (VPI) (*p*-value = 0.014), suggesting that vaccination-discouraging videos were generally uploaded earlier and had greater distribution and popularity than those encouraging vaccination (Table 6). This finding is consistent with a 2005 study examining factors associated with parental opposition to vaccination and showing that parents who refuse to vaccinate their children are more likely to search the Internet for information about HPV vaccines than parents of vaccinated children [77].

However, a significant difference was also found in the Video Source Reliability (*p*-value < 0.001) (Table 6 and Table 7) suggesting that vaccination-encouraging videos were generally uploaded by more trusted sources, rather than nonmedical YouTube users.

The main limitation of the present cross-sectional study of YouTube videos for mass-reach health communication and HPV vaccination promotion may be related to the dynamic nature of the YouTube platform, where new videos are uploaded daily, and the exclusion of non-English-language videos from the study that could have provided alternative perspectives on this topic.

Nonetheless, the present study may be the first to comprehensively assess the dissemination of information (topic coverage), popularity, accuracy, and reliability of YouTube content on oral HPV-related lesions, along with information on viral infection, clinical manifestation, and control measures, including vaccination, as well as epidemiologic data on HPV vaccine administration and HPV-related cancer prediction.

Further cross-sectional studies should be conducted without language restrictions, on other websites and social media platforms, and continuously updated.

In addition, the impact of mass-reach communication on patient awareness of HPV infection control measures, knowledge of HPV-related mucocutaneous and oral lesions, and attitudes toward HPV vaccination should be investigated.

## 5. Conclusions

A total of 97 videos were included in the present cross-sectional analysis of the accuracy of YouTube contents on HPV infection, oral and mucocutaneous lesions and vaccines, and their suitability for mass-reach health communication and the promotion of HPV vaccination strategies.

Most videos were uploaded by nonprofit or academic organizations (69%), and the mean Video Source Reliability (0–4 score) was 2.78 (Table 2), with the last criterion being “disclosure”. No dental care providers uploaded videos on this topic, suggesting that dentists and dental hygienists still play a minor role in disseminating knowledge about HPV infection and oral lesions and promoting HPV vaccination.

The mean Video Information and Quality Index (VIQI) (0–20) was 12 (Table 2), indicating that information flow and accuracy, consistency between video title and content, and quality of photos, animations, video captions, summary, etc., were generally moderate.

More than half of the videos (58%) were directed at laypersons (Figure 1), and thus likely uploaded to provide knowledge, raise awareness, and improve attitudes among nonprofessional YouTube users, who make up the majority of the vaccine’s target audience, such as preschool-aged children, adolescents, young adults, and their parents.

The oncogenic role of HPV was the most frequently discussed topic (92%) in the analyzed YouTube videos, followed by the role of HPV infection in female genital cancer (90%), the recommended age for HPV vaccination (88%), and the safety of HPV vaccines (81%). HPV-related cancers were generally a highly divulged topic, whereas HPV-related benign mucocutaneous lesions were a less covered topic, with oral lesions being the least covered topic (3%), revealing a poor dissemination of related information on YouTube. Similarly, the recognized risk factors for HPV infection and lesion development, as well as the related preventive strategies, although critical for primary prevention, were addressed in less than half of the videos. This last finding underscores the need to strengthen the potential role of dentists, who are often the first to recognize these lesions, in improving patient knowledge and awareness of the consequences of oral HPV infection and motivating patients to take infection control measures through both direct communication and population-based approaches, such as mass-reach communication.

About half (46%) of YouTube videos on HPV infection, oral lesions, and vaccination had very low/low educational value (GQS < 0.05), and only 15% and 2% had a good and excellent educational value, respectively.

Nearly 80% of videos advocated HPV vaccination, while 3% advised against it. However, vaccination-discouraging videos had a higher number of views and greater popularity compared to those that encouraged HPV vaccination. The need to improve gender-neutral HPV vaccination did not appear to be fully recognized, with only 39% of YouTube videos addressing this issue. Similarly, epidemiologic data on vaccination were barely reported, and projections of female and male genital cancers and oral carcinomas were not highlighted in the YouTube videos.

Overall, these data suggest that currently available YouTube videos on HPV infection, mucocutaneous and oral lesions, and vaccination were moderately accurate and reliable as a mass-reach communication tool. The limited role of oral and dental healthcare providers in uploading relevant content, with the poor dissemination of information about HPV-related benign and malignant oral lesions, may be expanded by purposefully using YouTube and other mass media to further improve patient knowledge about HPV-related oral lesions and promote HPV vaccination, which also underscores the potentially positive role of HPV vaccination on OSCC prevention. YouTube and other mass media videos about HPV infection, mucocutaneous and oral lesions, and vaccination may be appropriately created by reliable sources as a mass-reach communication tool, especially content about HPV-related oral lesions and pangender HPV vaccination targeting laypersons with simple and easy-to-understand language. Information flow and quality (titles, animations, images), as well as consistency between video titles and content), should be accurate, and video length should be limited to about 10 min for videos that cover all or most of the topics currently being analyzed, or shorter for those that describe fewer topics to allow for the accurate and complete delivery of information.

## Figures and Tables

**Table 1:** Inclusion and exclusion criteria of the analyzed YouTube videos.

	Inclusion Criteria	Exclusion Criteria
Video quality	≥240 p	<240 p
Video characteristics	Written explanations Spoken explanations	No written explanations No spoken explanations
Video content	HPV lesions and vaccination (as primary topics)	YouTube advertisements
Video language	English language	Non-English language

**Table 2:** Characteristics of the YouTube videos regarding HPV infection, mucocutaneous and oral lesions and vaccination.

	Mean	Median	SD ^1^	Minimum	Maximum
Videos length (min)	7.88	7.02	3.67	4.02	17.4
Number of views	27,026.6	414	102,701.42	5	695,711
Number of likes	375.77	3	1592.29	0	13,002
Number of dislikes	47.66	0	293.68	0	2796
Number of likes and dislikes disabled	0.082	0	0.28	0	1
Number of comments	134.1	1	575.29	0	4016
Number of comments disabled	0.3	0	0.46	0	1
Number of subscriptions	1.17 × 10^6^	20,700	5.36 × 10^6^	66	3.73 × 10^7^
Time elapsed since upload (days)	1527.82	1398	1254.38	5	4885
Video Power Index VPI	48.03	0.65	201.1	0	1597
Video Information and Quality Index VIQI (0–20 score)	12	12	3.39	5	20
Video Content (1–23 score)	9.91	10	3.1	3	16
Video Source Reliability (0–4 score)	2.78	3	0.83	1	5
Video educational value (0–5 score)	2.59	3	0.99	1	5

Abbreviations: ^1^ SD: standard deviation.

**Table 3:** Correlation between video’s educational value and video characteristics, popularity (VPI), general quality (VIQI), total content score and investigated topics, and source reliability, computed through Pearson’s correlation test.

Variables	Educational Value of YouTube Videos on HPV Vaccine	
	Pearson’s r	*p*-Value
Lenght	0.096	0.0498
Number of views	0.038	0.790
Number of likes	0.006	0.967
View ratio	0.036	0.799
Time elapsed since upload (days)	−0.040	0.777
Video Power Index (VPI)	−0.057	0.686
Video Information and Quality Index (VIQI)	0.105	0.460
Video Content (1–23 score)	0.076	0.593
Video Content on HPV:		
Ruote of transmission	0.237	0.091
Risk factors	0.262	0.061
Screening	0.213	0.130
Genotypes	0.104	0.465
Oncogenic role	−0.048	0.733
Skin lesions (benign)	−0.196	0.164
Mucosal lesions (benign)	0.083	0.558
Oral lesions (benign)	0.048	0.733
Genital cancer in females	−0.70	0.622
Genital cancer in males	0.003	0.985
Oral squamous cell carcinoma (OSCC)	0.035	0.808
Video Content on HPV vaccination:		
Age (recommended)	0.057	0.691
Gender	−0.341	0.013
Vaccine type(s)	−0.220	0.117
Vaccine safety	0.089	0.533
Messages encouraging vaccination	0.118	0.405
Messages discouraging vaccination	0.134	0.343
Messages neutral	−0.237	0.090
HPV vaccine Epidemiology:		
Vaccination course	0.073	0.608
Projections of genital cancer Fx	−0.053	0.707
Projection of genital cancer Mx	−0.026	0.853
Projection of squamous cell carcinoma (OSCC)	−0.014	0.920
Video Source Reliability	−0.033	0.816

**Table 4:** Characteristics of very-low/low- and medium/good/excellent-educational-value YouTube videos on HPV infection, mucocutaneous and oral lesions and vaccination.

YouTube Videos on HPV Infection, Mucocutaneous and Oral Lesions and Vaccination	Very Low/Low Educational Value			Medium/Good/Excellent Educational Value		
	Mean	Median	SD ^1^	Mean	Median	SD ^1^
length	7.44	6.33	3.27	8.26	7.25	3.976
Number of views	18,820.83	399.00	52,183.53	34,127.75	454.00	131,886.640
Number of likes	412.07	6	1945.15	344.37	3.00	1227.208
Number of dislikes	75.00	0	416.81	24.00	0.00	105.958
Time elapsed since upload (days)	123.2667	826	1204.674	16,784.4615	1574.500	1251.018
Video Power Index (VPI)	68.4531	1.45	256.571	30.3554	0.405	136.542
Video Information and Quality Index (VIQI)	12.9556	12	3.337	11.1731	11.000	3.240
Video Content (1–23 score)	9.7333	10	3.333	10.0577	10.000	2.718
Video Content on HPV:						
Ruote of transmission	0.5556	1	0.503	0.6731	1.000	0.474
Risk factors	0.3556	0	0.484	0.5385	1.000	0.503
Screening	0.3778	0	0.490	0.5769	1.000	0.499
Genotypes	0.4222	0	0.499	0.3462	0.000	0.480
Oncogenic role	0.8444	1	0.397	0.9615	1.000	0.194
Skin lesions (benign)	0.2000	0	0.405	0.2308	0.000	0.425
Mucosal lesions (benign)	0.2222	0	0.420	0.2115	0.000	0.412
Oral lesions (benign)	0.0222	0	0.149	0.0385	0.000	0.194
Genital cancer in females	0.8667	1	0.344	0.9231	1.000	0.269
Genital cancer in males	0.6667	1	0.477	0.5769	1.000	0.499
Oral squamous cell carcinoma (OSCC)	0.4444	0	0.503	0.5000	0.500	0.505
Video Content on HPV vaccination:						
Age (recommended)	0.8889	1	0.318	0.8654	1.000	0.345
Gender	0.6000	1	0.495	0.2115	0.000	0.412
Vaccine type(s)	0.4444	0	0.503	0.2500	0.000	0.437
Vaccine safety	0.7778	1	0.420	0.8462	1.000	0.364
Messages encouraging vaccination	0.7556	1	0.435	0.8269	1.000	0.382
Messages discouraging vaccination	0.0000	0	0.000	0.0577	0.000	0.235
Messages neutral	0.2667	0	0.447	0.1158	0.000	0.323
HPV vaccine Epidemiology:						
Vaccination course	0.4889	0	0.506	0.5769	1.000	0.499
Projections of genital cancer Fx	0.3333	0	0.477	0.4615	0.000	0.503
Projection of genital cancer Mx	0.1333	0	0.344	0.1731	0.000	0.382
Projection of oral squamous cell carcinoma (OSCC)	0.0444	0	0.208	0.0577	0.000	0.235
Video Source Reliability (0–4 score) Source:	2.9111	3	0.821	2.6731	3.000	0.834
Dentalcare provider (source)	0.0000	0	0.000	0.0000	0.000	0.000
Other healthcare provider (source)	0.1111	0	0.318	0.0769	0.000	0.269
University/hospital (source)	0.1556	0	0.367	0.1923	0.000	0.398
Pharmaceutical Industries (source)	0.0000	0	0.000	0.0000	0.000	0.000
Commercial (source)	0.0000	0	0.000	0.0769	0.000	0.269
Other (source)	0.7333	0	0.447	0.6538	1.000	0.480
Video educational value (0–5 score) Target Audience:	1.6889	2	0.468	3.37	3.00	0.561
Professionals	0.2889	0	0.458	0.0962	0.000	0.298
Laypersons	0.6000	1	0.495	0.5769	1.000	0.499
Both	0.1556	0	0.367	0.3269	0.000	0.474

Abbreviations: ^1^ SD: standard deviation.

**Table 5:** Comparison of variables between very-low/low- and medium/good/excellent-educational-value YouTube videos on HPV infection, mucocutaneous and oral lesions and vaccination.

Very-Low/Low- vs. Medium/Good/Excellent-Educational-Value YouTube Videos on HPV	Mann–Whitney U Test	*p*-Value
Length	1043	0.358
Number of views	1121	0.726
Time elapsed since upload (days)	852	0.022
Video Power Index (VPI)	1040	0.340
Target Audience:		
Laypersons	1143	0.822
Professional	945	0.016
Both	970	0.053
Video Information and Quality Index (VIQI) (0–20 score)	836	0.015
Video Content (1–23 score)	1122	0.730
Video Content HPV vaccine:		
Encouraging vaccination	1087	0.392
Discouraging vaccination	1103	0.106
Neutral toward vaccination	993	0.058
Fake news	1125	0.191
Video Source Reliability based on the JAMA benchmark criteria (0–4 score)	1005	0.183

**Table 6:** Characteristics of vaccination-encouraging and -discouraging YouTube videos on HPV infection, mucocutaneous and oral lesions and vaccination.

YouTube Videos on HPV Infection, Mucocutaneous and Oral Lesions and Vaccination	Vaccination-Encouraging			Vaccination-Discouraging		
	Mean	Median	SD ^1^	Mean	Median	SD ^1^
Lenght	7.7897	6.500	3.648	6.425	6.450	2.385
Number of views	18,651.2141	409.500	80,677.360	103,256.750	104,008.500	112,548.024
Time elapsed since upload (days)	1454.7895	1241.000	1222.437	1715.750	1542.500	1530.998
Video Power Index (VPI) Target Audience:	55.9254	0.610	225.491	14.380	5.975	21.015
Laypersons	0.5526	1.000	0.5526	0.750	1.000	0.500
Professional	0.1974	0.000	0.1974	0.250	0.000	0.500
Both	0.2500	0.000	0.2500	0.500	0.500	0.577
Video Source Reliability based on the JAMA benchmark criteria (0–4 score)	2.8553	3.000	0.828	2.250	2.500	0.957
Video Information and Quality Index (VIQI)	12.1974	12.000	3.468	10.250	8.500	3.862
Video Content (1–23 score)	9.9211	10.000	2.902	7.000	7.000	3.367
Video Content on HPV-related lesions:						
Oral lesions (benign)	0.0263	0.000	0.161	0.000	0.000	0.000
Mucosal lesions (benign)	0.2105	0.000	0.410	0.000	0.000	0.000
Skin lesions (benign)	0.2500	0.000	0.436	0.000	0.000	0.000
Genital cancer in females	0.9211	1.000	0.271	0.500	0.500	0.577
Genital cancer in males	0.6316	1.000	0.486	0.500	0.500	0.577
Oral squamous cell carcinoma (OSCC)	0.4868	0.000	0.503	0.000	0.000	0.000
Video Content on HPV vaccine:						
Age	0.8816	1.000	0.325	0.750	1.000	0.500
Gender	0.4868	0.000	0.503	0.250	0.000	0.500
Vaccine types	0.3947	0.000	0.492	0.250	0.000	0.500
Vaccine safety	0.7763	1.000	0.419	1.000	1.000	0.000
Fake news	0.0000	0.000	0.000	0.000	0.000	0.000
Video Content on Epidemiology:						
Vaccination course	0.5263	1.000	0.503	0.500	0.500	0.577
Projections of genital cancer in females	0.3947	0.000	0.492	0.250	0.000	0.500
Projections of genital cancer in males	0.1447	0.000	0.354	0.250	0.000	0.500
Projections of oral squamous cell carcinoma (OSCC)	0.0526	0.000	0.225	0.000	0.000	0.000
Video Educational value	2.3289	2.000	0.700	1.000	1.000	0.000

Abbreviations: ^1^ SD: standard deviation.

**Table 7:** Comparison of variables between HPV vaccination-encouraging and -discouraging YouTube videos on HPV infection, mucocutaneous and oral lesions and vaccination.

YouTube Videos Encouraging vs. Discouraging Vaccination	Mann–Whitney U Test	*p*-Value
Length of video	100	0.256
Number of views	35	0.010
Time elapsed since upload (days)	55	0.033
Video Power Index (VPI)	41.5	0.014
Target Audience:		
Laypersons	90.0	0.108
Professionals	126.0	0.378
Both	112.0	0.245
Video Information and Quality Index (VIQI) (0–20 score)	70.5	0.072
Video Content (1–23 score)	63	0.049
Video Content on HPV-related lesions:		
Oral lesions (benign)	148.0	0.775
Mucosal lesions (benign)	120.0	0.316
Skin lesions (benign)	122.0	0.335
Genital cancer in females	86.0	0.003 *
Genital cancer in males	94.0	0.133
Oral squamous cell carcinoma (OSCC)	72.0	0.043
Video Content on HPV vaccine:		
Age	92.0	0.022
Gender	104.0	0.178
Vaccine types	78.0	0.038 *
Vaccine safety	136.0	0.580
Video Content on Epidemiology:		
Vaccination course	66.0	0.029
Projections of genital cancer in females	124.0	0.478
Projections of genital cancer in males	126.0	0.378
Projections of oral squamous cell carcinoma (OSCC)	142.0	0.617
Video Educational value	116	0.415
Video Source Reliability based on the JAMA benchmark criteria (0–4 score)	17	<0.001

* statistically significant.

## Data Availability

Data supporting reported results can be found in Scopus, Web of Science, and MEDLINE/PubMed databases, and on YouTube.

## Supplementary Materials

The following supporting information can be downloaded at: https://www.mdpi.com/article/10.3390/ijerph20115972/s1, Table S1: Data extracted and computed from the videos included.
