# Supplementary material for: HPV-Related Oral Lesions: YouTube Videos Suitability for Preventive Interventions including Mass-Reach Health Communication and Promotion of HPV Vaccination
Source: Int J Environ Res Public Health. 2023 May 27;20(11):5972. doi: 10.3390/ijerph20115972 (PMC10252756; doi:10.3390/ijerph20115972)
Supplement: Supplementary file 1 [file ijerph-20-05972-s001.zip › ijerph-2264891-supplementary.pdf]

Table S1. Data extracted and computed from the videos included.

Characteristics	Source	Popularity	Information and Quality	Content: HPV infection and related lesions	Content: HPV vaccination	Content: Epidemiology, Total-Content score and GQS
Link:<br>https://www.youtube.com/watch?app=desktop&v=1jEtDE7_wWc<br><br>Lenght (min): 4.44<br><br>Time elapsed since upload (days): 1538<br><br>Target audience: Both	Source: Other<br><br>**Video Source Reliability (JAMA): 3**	Views (n.204861)<br>Likes (n.995)<br>Dislikes (n.2796)<br>Comments (n.4016)<br>Subscriptions (n.330000)<br><br>Like Ratio: 1.85<br>View Ratio: 246.419<br><br>**VPI: 4.55**	Information flow (2)<br>Information accuracy (2)<br>Quality (1)<br>Sensitivity (3)<br><br>**VIQI: 8**	Route of transmission (0)<br>HPV risk factors (0)<br>HPV screening (0)<br>Genotypes (0)<br>Oncogenic role (0)<br><br>Skin lesions (benign) (0)<br>Mucosal lesions (benign) (0)<br>Oral lesions (benign) (0)<br><br>Genital cancer Fx (0)<br>Genital cancer Mx (0)<br>OSCC (0)<br><br>**Content on HPV infection and related lesions Score: 0**	Age (0)<br>Gender (1)<br>Type of vaccines (0)<br>HPV vaccine safety (1)<br><br>HPV vaccination advice:<br>Encouraging (1)<br>Discouraging (0)<br>Neutral (0)<br><br>Fake news (0)<br><br>**Content on HPV vaccination Score: 3**	Vaccination course (0)<br>Projections of genital cancer Fx (0)<br>Projections of genital cancer Mx (0)<br>Projection of OSCC (0)<br><br>**Content on Epidemiology Score: 0**<br><br>**VIDEO TOTAL-CONTENT SCORE: 3**<br><br>**GQS: 1**<br>**Video Educational Value:** very low/low
Link:<br>https://www.youtube.com/watch?app=desktop&v=wQSTUIw8_1U<br><br>Lenght (min): 8.46<br><br>Time elapsed since upload (days): 3748<br><br>Target audience: Laypersons	Source: Other healthcare providers (General Practioner)<br><br>**Video Source Reliability (JAMA): 2**	Views (n.196327)<br>Likes (n.1529)<br>Dislikes (n.198)<br>Comments (n.1075)<br>Subscriptions (n.167000)<br><br>Like Ratio: 0.87<br>View Ratio: 5238.18<br><br>**VPI: 45.57**	Information flow (4)<br>Information accuracy (3)<br>Quality (5)<br>Sensitivity (4)<br><br>**VIQI: 16**	Route of transmission (1)<br>HPV risk factors (1)<br>HPV screening (1)<br>Genotypes (0)<br>Oncogenic role (1)<br><br>Skin lesions (benign) (0)<br>Mucosal lesions (benign) (0)<br>Oral lesions (benign) (0)<br><br>Genital cancer Fx (1)<br>Genital cancer Mx (1)<br>OSCC (0)<br><br>**Content on HPV infection and related lesions Score: 6**	Age (1)<br>Gender (0)<br>Type of vaccines (1)<br>HPV vaccine safety (1)<br><br>HPV vaccination advice:<br>Encouraging (1)<br>Discouraging (0)<br>Neutral (0)<br><br>Fake news (0)<br><br>**Content on HPV vaccination Score: 4**	Vaccination course (0)<br>Projections of genital cancer Fx (1)<br>Projections of genital cancer Mx (0)<br>Projection of OSCC (0)<br><br>**Content on Epidemiology Score: 1**<br><br>**VIDEO TOTAL-CONTENT SCORE: 11**<br><br>**GQS: 4**<br>**Video Educational Value:** medium/good/excellent
Link:<br>https://www.youtube.com/watch?app=desktop&v=lCpVD8fsSjI	Source: Other	Views (n.11690)<br>Likes (n.98)<br>Dislikes (n.17)	Information flow (3)	Route of transmission (0)<br>HPV risk factors (0)<br>HPV screening (0)	Age (1)<br>Gender (0)<br>Type of vaccines (0)	Vaccination course (1)<br>Projections of genital cancer Fx (0)

Lenght (min): 4.28<br><br>Time elapsed since upload (days): 1547<br><br>Target audience: Both	**Video Source Reliability (JAMA): 3**	Comments (n.0)<br>Subscriptions (n.2970)<br><br>Like Ratio: 0.98<br>View Ratio: 755.65<br><br>**VPI: 7.40**	Information accuracy (3)<br>Quality (1)<br>Sensitivity (2)<br><br>**VIQI: 9**	Genotypes (0)<br>Oncogenic role (1)<br><br>Skin lesions (benign) (0)<br>Mucosal lesions (benign) (0)<br>Oral lesions (benign) (0)<br><br>Genital cancer Fx (1)<br>Genital cancer Mx (0)<br>OSCC (0)<br><br>**Content on HPV infection and related lesions Score: 2**	HPV vaccine safety (1)<br><br>HPV vaccination advice:<br>Encouraging (1)<br>Discouraging (0)<br>Neutral (0)<br><br>Fake news (0)<br><br>**Content on HPV vaccination Score: 3**	Projections of genital cancer Mx (0)<br>Projection of OSCC(0)<br><br>**Content on Epidemiology Score: 1**<br><br>**VIDEO TOTAL-CONTENT SCORE: 6**<br><br>**GQS: 2**<br>**Video Educational Value:** very low/low
Link: https://www.youtube.com/watch?app=desktop&v=qFByNSwSFXQ<br><br>Lenght (min): 8.52<br><br>Time elapsed since upload (days): 30<br><br>Target audience: Laypersons	Source: Other<br><br>**Video Source Reliability (JAMA): 1**	Views (n.149)<br>Likes (n.0)<br>Dislikes (n.0)<br>Comments (n.0)<br>Subscriptions (n.2080)<br><br>Like Ratio: 0<br>View Ratio: 496.66<br><br>**VPI: 0**	Information flow (2)<br>Information accuracy (2)<br>Quality (1)<br>Sensitivity (3)<br><br>**VIQI: 8**	Route of transmission (1)<br>HPV risk factors (0)<br>HPV screening (0)<br>Genotypes (0)<br>Oncogenic role (1)<br><br>Skin lesions (benign) (0)<br>Mucosal lesions (benign) (0)<br>Oral lesions (benign) (0)<br><br>Genital cancer Fx (0)<br>Genital cancer Mx (1)<br>OSCC (0)<br><br>**Content on HPV infection and related lesions Score: 3**	Age (1)<br>Gender (0)<br>Type of vaccines (0)<br>HPV vaccine safety (1)<br><br>HPV vaccination advice:<br>Encouraging (1)<br>Discouraging (0)<br>Neutral (0)<br><br>Fake news (0)<br><br>**Content on HPV vaccination Score: 3**	Vaccination course (1)<br>Projections of genital cancer Fx (0)<br>Projections of genital cancer Mx (1)<br>Projection of OSCC (0)<br><br>**Content on Epidemiology Score: 2**<br><br>**VIDEO TOTAL-CONTENT SCORE: 8**<br><br>**GQS: 2**<br>**Video Educational Value:** very low/low
Link: https://www.youtube.com/watch?v=J3Q-1oKpa-c<br><br>Lenght (min): 4.46<br><br>Time elapsed since upload (days): 847<br><br>Target audience: Laypersons	Source: Other<br><br>**Video Source Reliability (JAMA): 3**	Views (n.12147)<br>Likes (n.17)<br>Dislikes (n.2)<br>Comments (n.1)<br>Subscriptions (n.1090)<br><br>Like Ratio: 0.16<br>View Ratio: 1424.12<br><br>**VPI: 2.23**	Information flow (3)<br>Information accuracy (2)<br>Quality (2)<br>Sensitivity (2)<br><br>**VIQI: 9**	Route of transmission (0)<br>HPV risk factors (0)<br>HPV screening (0)<br>Genotypes (0)<br>Oncogenic role (1)<br><br>Skin lesions (benign) (0)<br>Mucosal lesions (benign) (0)<br>Oral lesions (benign) (0)	Age (1)<br>Gender (0)<br>Type of vaccines (0)<br>HPV vaccine safety (1)<br><br>HPV vaccination advice:<br>Encouraging (1)<br>Discouraging (0)<br>Neutral (0)<br><br>Fake news (0)	Vaccination course (0)<br>Projections of genital cancer Fx (1)<br>Projections of genital cancer Mx (0)<br>Projection of OSCC (0)<br><br>**Content on Epidemiology Score: 1**

				Genital cancer Fx (1)<br>Genital cancer Mx (0)<br>OSCC (0)<br><br>**Content on HPV infection and related lesions Score: 2**	**Content on HPV vaccination Score: 3**	**VIDEO TOTAL-CONTENT SCORE: 6**<br><br>**GQS: 1**<br>**Video Educational Value:** very low/low
Link: https://www.youtube.com/watch?v=I1LrOWwIgCg<br><br>Lenght (min): 4.16<br><br>Time elapsed since upload (days): 1869<br><br>Target audience: Laypersons	Source: Other<br><br>**Video Source Reliability (JAMA): 4**	Views (n.128272)<br>Likes (n.576)<br>Dislikes (n.36)<br>Comments (n.43)<br>Subscriptions (n.224000)<br><br>Like Ratio: 0.48<br>View Ratio: 6863.13<br><br>**VPI: 32.94**	Information flow (4)<br>Information accuracy (5)<br>Quality (4)<br>Sensitivity (5)<br><br>**VIQI: 18**	Route of transmission (0)<br>HPV risk factors (0)<br>HPV screening (1)<br>Genotypes (1)<br>Oncogenic role (1)<br><br>Skin lesions (benign) (1)<br>Mucosal lesions (benign) (1)<br>Oral lesions (benign) (0)<br><br>Genital cancer Fx (1)<br>Genital cancer Mx (1)<br>OSCC (1)<br><br>**Content on HPV infection and related lesions Score: 8**	Age (1)<br>Gender (1)<br>Type of vaccines (0)<br>HPV vaccine safety (0)<br><br>HPV vaccination advice:<br>Encouraging (0)<br>Discouraging (0)<br>Neutral (1)<br><br>Fake news (0)<br><br>**Content on HPV vaccination Score: 3**	Vaccination course (1)<br>Projections of genital cancer Fx (1)<br>Projections of genital cancer Mx (1)<br>Projection of OSCC (0)<br><br>**Content on Epidemiology Score: 3**<br><br>**VIDEO TOTAL-CONTENT SCORE: 14**<br><br>**GQS: 4**<br>**Video Educational Value:** medium/good/excellent
Link: https://www.youtube.com/watch?v=X17nhkmk3pc<br><br>Lenght (min): 8.12<br><br>Time elapsed since upload (days): 91<br><br>Target audience: Professional	Source: Other<br><br>**Video Source Reliability (JAMA): 3**	Views (n.158)<br>Likes (n.6)<br>Dislikes (n.0)<br>Comments (n.0)<br>Subscriptions (n.10100)<br><br>Like Ratio: 3.80<br>View Ratio: 173.63<br><br>**VPI: 6.60**	Information flow (3)<br>Information accuracy (3)<br>Quality (3)<br>Sensitivity (5)<br><br>**VIQI: 14**	Route of transmission (1)<br>HPV risk factors (0)<br>HPV screening (1)<br>Genotypes (0)<br>Oncogenic role (1)<br><br>Skin lesions (benign) (0)<br>Mucosal lesions (benign) (0)<br>Oral lesions (benign) (0)<br><br>Genital cancer Fx (1)<br>Genital cancer Mx (0)<br>OSCC (0)<br><br>**Content on HPV infection and related lesions Score: 4**	Age (1)<br>Gender (1)<br>Type of vaccines (0)<br>HPV vaccine safety (1)<br><br>HPV vaccination advice:<br>Encouraging (1)<br>Discouraging (0)<br>Neutral (0)<br><br>Fake news (0)<br><br>**Content on HPV vaccination Score: 4**	Vaccination course (0)<br>Projections of genital cancer Fx (0)<br>Projections of genital cancer Mx (0)<br>Projection of OSCC (0)<br><br>**Content on Epidemiology Score: 1**<br><br>**VIDEO TOTAL-CONTENT SCORE: 8**<br><br>**GQS: 2**<br>**Video Educational Value:** very low/low

Link: https://www.youtube.com/watch?v=i5UkEb8GxN4<br><br>Lenght (min): 4.34<br><br>Time elapsed since upload (days): 159<br><br>Target audience: Both	Source: Other<br><br>**Video Source Reliability (JAMA): 3**	Views (n.737)<br>Likes (n.12)<br>Dislikes (n.0)<br>Comments (n.26)<br>Subscriptions (n.341000)<br><br>Like Ratio: 1.63<br>View Ratio: 453.52<br><br>**VPI: 7.55**	Information flow (4)<br>Information accuracy (4)<br>Quality (3)<br>Sensitivity (5)<br><br>**VIQI: 16**	Route of transmission (0)<br>HPV risk factors (0)<br>HPV screening (0)<br>Genotypes (0)<br>Oncogenic role (1)<br><br>Skin lesions (benign) (0)<br>Mucosal lesions (benign) (0)<br>Oral lesions (benign) (0)<br><br>Genital cancer Fx (1)<br>Genital cancer Mx (1)<br>OSCC (1)<br><br>**Content on HPV infection and related lesions Score: 4**	Age (1)<br>Gender (1)<br>Type of vaccines (0)<br>HPV vaccine safety (1)<br><br>HPV vaccination advice:<br>Encouraging (1)<br>Discouraging (0)<br>Neutral (0)<br><br>Fake news (0)<br><br>**Content on HPV vaccination Score: 4**	Vaccination course (0)<br>Projections of genital cancer Fx (0)<br>Projections of genital cancer Mx (0)<br>Projection of OSCC (0)<br><br>**Content on Epidemiology Score: 0**<br><br>**VIDEO TOTAL-CONTENT SCORE: 8**<br><br>**GQS: 3**<br>**Video Educational Value:**<br>medium/good/excellent
Link: https://www.youtube.com/watch?v=UvqFRsblx74<br><br>Lenght (min): 15.34<br><br>Time elapsed since upload (days): 826<br><br>Target audience: Laypersons	Source: Other healthcare provider (Obstetrician-gynecologist)<br><br>**Video Source Reliability (JAMA): 5**	Views (n.224403)<br>Likes (n.13002)<br>Dislikes (n.190)<br>Comments (n.3684)<br>Subscriptions (n.1240000)<br><br>Like Ratio: 5.88<br>View Ratio: 27167.43<br><br>**VPI: 1597.44**	Information flow (5)<br>Information accuracy (5)<br>Quality (3)<br>Sensitivity (5)<br><br>**VIQI: 18**	Route of transmission (0)<br>HPV risk factors (0)<br>HPV screening (0)<br>Genotypes (1)<br>Oncogenic role (1)<br><br>Skin lesions (benign) (0)<br>Mucosal lesions (benign) (1)<br>Oral lesions (benign) (0)<br><br>Genital cancer Fx (1)<br>Genital cancer Mx (1)<br>OSCC (1)<br><br>**Content on HPV infection and related lesions Score: 6**	Age (1)<br>Gender (1)<br>Type of vaccines (1)<br>HPV vaccine safety (1)<br><br>HPV vaccination advice:<br>Encouraging (1)<br>Discouraging (0)<br>Neutral (0)<br><br>Fake news (0)<br><br>**Content on HPV vaccination Score: 5**	Vaccination course (1)<br>Projections of genital cancer Fx (1)<br>Projections of genital cancer Mx (0)<br>Projection of OSCC (0)<br><br>**Content on Epidemiology Score: 2**<br><br>**VIDEO TOTAL-CONTENT SCORE: 13**<br><br>**GQS: 4**<br>**Video Educational Value:**<br>medium/good/excellent
Link: https://www.youtube.com/watch?v=ZkrRC3pmFps<br><br>Lenght (min): 7.26<br><br>Time elapsed since upload (days): 442<br><br>Target audience: Professional	Source: Other<br><br>**Video Source Reliability (JAMA): 3**	Views (n.125)<br>Likes (n.0)<br>Dislikes (n.0)<br>Comments (disabled)<br>Subscriptions (n.411)	Information flow (3)<br>Information accuracy (3)<br>Quality (3)<br>Sensitivity (3)<br><br>**VIQI: 12**	Route of transmission (0)<br>HPV risk factors (0)<br>HPV screening (0)<br>Genotypes (0)<br>Oncogenic role (0)<br><br>Skin lesions (benign) (0)	Age (1)<br>Gender (1)<br>Type of vaccines (0)<br>HPV vaccine safety (1)<br><br>HPV vaccination advice:<br>Encouraging (1)<br>Discouraging (0)	Vaccination course (1)<br>Projections of genital cancer Fx (0)<br>Projections of genital cancer Mx (0)<br>Projection of OSCC (0)

		Like Ratio: 0<br>View Ratio: 92.99<br><br>**VPI: 0**		Mucosal lesions (benign) (0)<br>Oral lesions (benign) (0)<br><br>Genital cancer Fx (0)<br>Genital cancer Mx (0)<br>OSCC (0)<br><br>**Content on HPV infection and related lesions Score: 0**	Neutral (0)<br><br>Fake news (0)<br><br>**Content on HPV vaccination Score: 4**	**Content on Epidemiology Score: 1**<br><br>**VIDEO TOTAL-CONTENT SCORE: 5**<br><br>**GQS: 1**<br>**Video Educational Value:** very low/low
Link: https://www.youtube.com/watch?v=5v6bcIguJBg<br><br>Lenght (min): 6.15<br><br>Time elapsed since upload (days): 669<br><br>Target audience: Professional	Source: Other<br><br>**Video Source Reliability (JAMA): 4**	Views (n.78)<br>Likes (n.0)<br>Dislikes (n.0)<br>Comments (disabled)<br>Subscriptions (n.411)<br><br>Like Ratio: 0<br>View Ratio: 13.95<br><br>**VPI: 0**	Information flow (3)<br>Information accuracy (3)<br>Quality (3)<br>Sensitivity (3)<br><br>**VIQI: 12**	Route of transmission (0)<br>HPV risk factors (0)<br>HPV screening (0)<br>Genotypes (0)<br>Oncogenic role (0)<br><br>Skin lesions (benign) (0)<br>Mucosal lesions (benign) (0)<br>Oral lesions (benign) (0)<br><br>Genital cancer Fx (0)<br>Genital cancer Mx (0)<br>OSCC (0)<br><br>**Content on HPV infection and related lesions Score: 0**	Age (1)<br>Gender (0)<br>Type of vaccines (0)<br>HPV vaccine safety (0)<br><br>HPV vaccination advice:<br>Encouraging (1)<br>Discouraging (0)<br>Neutral (0)<br><br>Fake news (0)<br><br>**Content on HPV vaccination Score: 2**	Vaccination course (1)<br>Projections of genital cancer Fx (0)<br>Projections of genital cancer Mx (0)<br>Projection of OSCC (0)<br><br>**Content on Epidemiology Score: 1**<br><br>**VIDEO TOTAL-CONTENT SCORE: 3**<br><br>**GQS: 1**<br>**Video Educational Value:** very low/low
Link: https://www.youtube.com/watch?v=ayVB27cDgNQ<br><br>Lenght (min): 4.31<br><br>Time elapsed since upload (days): 697<br><br>Target audience: Laypersons	Source: Other<br><br>**Video Source Reliability (JAMA): 1**	Views (n.399)<br>Likes (n.2)<br>Dislikes (n.6)<br>Comments (n.5)<br>Subscriptions (n.321000)<br><br>Like Ratio: 2.51<br>View Ratio: 57.25<br><br>**VPI: 1.43**	Information flow (4)<br>Information accuracy (2)<br>Quality (3)<br>Sensitivity (4)<br><br>**VIQI: 13**	Route of transmission (0)<br>HPV risk factors (0)<br>HPV screening (0)<br>Genotypes (0)<br>Oncogenic role (1)<br><br>Skin lesions (benign) (0)<br>Mucosal lesions (benign) (0)<br>Oral lesions (benign) (0)<br><br>Genital cancer Fx (1)<br>Genital cancer Mx (1)<br>OSCC (1)	Age (1)<br>Gender (1)<br>Type of vaccines (0)<br>HPV vaccine safety (1)<br><br>HPV vaccination advice:<br>Encouraging (1)<br>Discouraging (0)<br>Neutral (0)<br><br>Fake news (0)<br><br>**Content on HPV vaccination Score: 4**	Vaccination course (0)<br>Projections of genital cancer Fx (0)<br>Projections of genital cancer Mx (0)<br>Projection of OSCC (0)<br><br>**Content on Epidemiology Score: 0**<br><br>**VIDEO TOTAL-CONTENT SCORE: 8**<br><br>**GQS: 3**

				**Content on HPV infection and related lesions Score: 4**		**Video Educational Value:** medium/good/excellent
Link: https://www.youtube.com/watch?v=aTOPAIr62n8<br><br>Lenght (min): 5.40<br><br>Time elapsed since upload (days): 4885<br><br>Target audience: Laypersons	Source: Other<br><br>**Video Source Reliability (JAMA): 3**	Views (n.37)<br>Likes (n.0)<br>Dislikes (n.0)<br>Comments (n.0)<br>Subscriptions (n.14600)<br><br>Like Ratio: 0<br>View Ratio: 0.76<br><br>**VPI: 0**	Information flow (2)<br>Information accuracy (2)<br>Quality (2)<br>Sensitivity (3)<br><br>**VIQI: 9**	Route of transmission (1)<br>HPV risk factors (1)<br>HPV screening (0)<br>Genotypes (0)<br>Oncogenic role (1)<br><br>Skin lesions (benign) (0)<br>Mucosal lesions (benign) (0)<br>Oral lesions (benign) (0)<br><br>Genital cancer Fx (1)<br>Genital cancer Mx (0)<br>OSCC (0)<br><br>**Content on HPV infection and related lesions Score: 4**	Age (1)<br>Gender (0)<br>Type of vaccines (1)<br>HPV vaccine safety (1)<br><br>HPV vaccination advice:<br>Encouraging (1)<br>Discouraging (0)<br>Neutral (0)<br><br>Fake news (0)<br><br>**Content on HPV vaccination Score: 4**	Vaccination course (1)<br>Projections of genital cancer Fx (1)<br>Projections of genital cancer Mx (0)<br>Projection of OSCC (0)<br><br>**Content on Epidemiology Score: 2**<br><br>**VIDEO TOTAL-CONTENT SCORE: 10**<br><br>**GQS: 2**<br>**Video Educational Value:** very low/low
Link:<br>https://www.youtube.com/watch?app=desktop&v=OuwcOyMjAzc<br><br>Lenght (min): 5.39<br><br>Time elapsed since upload (days): 1804<br><br>Target audience: Laypersons	Source: Other<br><br>**Video Source Reliability (JAMA): 3**	Views (n.28544)<br>Likes (disabled)<br>Dislikes (disabled)<br>Comments (disabled)<br>Subscriptions (26700)<br><br>Like Ratio: Not evaluable<br>View Ratio: 1582.26<br><br>**VPI: Not evaluable**	Information flow (3)<br>Information accuracy (3)<br>Quality (3)<br>Sensitivity (3)<br><br>**VIQI: 12**	Route of transmission (0)<br>HPV risk factors (0)<br>HPV screening (1)<br>Genotypes (0)<br>Oncogenic role (1)<br><br>Skin lesions (benign) (0)<br>Mucosal lesions (benign) (0)<br>Oral lesions (benign) (0)<br><br>Genital cancer Fx (1)<br>Genital cancer Mx (0)<br>OSCC (0)<br><br>**Content on HPV infection and related lesions Score: 3**	Age (1)<br>Gender (0)<br>Type of vaccines (1)<br>HPV vaccine safety (1)<br><br>HPV vaccination advice:<br>Encouraging (1)<br>Discouraging (0)<br>Neutral (0)<br><br>Fake news (0)<br><br>**Content on HPV vaccination Score: 4**	Vaccination course (1)<br>Projections of genital cancer Fx (1)<br>Projections of genital cancer Mx (0)<br>Projection of OSCC (0)<br><br>**Content on Epidemiology Score: 2**<br><br>**VIDEO TOTAL-CONTENT SCORE: 9**<br><br>**GQS: 3**<br>**Video Educational Value:** medium/good/excellent
Link:<br>https://www.youtube.com/watch?app=desktop&v=zyAKzbp_RSk<br><br>Lenght (min): 5.10	Source: Other<br><br>**Video Source Reliability (JAMA): 3**	Views (n.38692)<br>Likes (disabled)<br>Dislikes (disabled)<br>Comments (disabled)	Information flow (3)<br>Information accuracy (2)<br>Quality (3)	Route of transmission (1)<br>HPV risk factors (0)<br>HPV screening (0)<br>Genotypes (0)<br>Oncogenic role (1)	Age (1)<br>Gender (0)<br>Type of vaccines (0)<br>HPV vaccine safety (1)	Vaccination course (1)<br>Projections of genital cancer Fx (0)<br>Projections of genital cancer Mx (0)

Time elapsed since upload (days): 1120<br><br>Target audience: Laypersons		Subscriptions (16600)<br><br>Like Ratio: Not evaluable<br>View Ratio: 3457.27<br><br>**VPI: Not evaluable**	Sensitivity (3)<br><br>**VIQI: 11**	Skin lesions (benign) (0)<br>Mucosal lesions (benign) (0)<br>Oral lesions (benign) (0)<br><br>Genital cancer Fx (1)<br>Genital cancer Mx (0)<br>OSCC (0)<br><br>**Content on HPV infection and related lesions Score: 3**	HPV vaccination advice:<br>Encouraging (1)<br>Discouraging (0)<br>Neutral (0)<br><br>Fake news (0)<br><br>**Content on HPV vaccination Score: 3**	Projection of OSCC (0)<br><br>**Content on Epidemiology Score: 1**<br><br>**VIDEO TOTAL-CONTENT SCORE: 7**<br><br>**GQS: 3**<br>**Video Educational Value:** medium/good/excellent
Link: https://www.youtube.com/watch?v=63zhnOpko5E<br><br>Lenght (min): 5.23<br><br>Time elapsed since upload (days): 40<br><br>Target audience: Laypersons	Source: Other healthcare provider (General Practitioner)<br><br>**Video Source Reliability (JAMA): 4**	Views (n.207)<br>Likes (n.12)<br>Dislikes (n.0)<br>Comments (n.1)<br>Subscriptions (n.39200)<br><br>Like Ratio: 5.80<br>View Ratio: 517.50<br><br>**VPI: 30.01**	Information flow (4)<br>Information accuracy (4)<br>Quality (5)<br>Sensitivity (4)<br><br>**VIQI: 17**	Route of transmission (1)<br>HPV risk factors (1)<br>HPV screening (1)<br>Genotypes (1)<br>Oncogenic role (1)<br><br>Skin lesions (benign) (1)<br>Mucosal lesions (benign) (1)<br>Oral lesions (benign) (1)<br><br>Genital cancer Fx (1)<br>Genital cancer Mx (1)<br>OSCC (1)<br><br>**Content on HPV infection and related lesions Score: 11**	Age (1)<br>Gender (1)<br>Type of vaccines (1)<br>HPV vaccine safety (1)<br><br>HPV vaccination advice:<br>Encouraging (0)<br>Discouraging (0)<br>Neutral (1)<br><br>Fake news (0)<br><br>**Content on HPV vaccination Score: 5**	Vaccination course (0)<br>Projections of genital cancer Fx (0)<br>Projections of genital cancer Mx (0)<br>Projection of OSCC (0)<br><br>**Content on Epidemiology Score: 0**<br><br>**VIDEO TOTAL-CONTENT SCORE: 16**<br><br>**GQS: 4**<br>**Video Educational Value:** medium/good/excellent
Link: https://www.youtube.com/watch?v=DExJGe1uz7g<br><br>Lenght (min): 11.56<br><br>Time elapsed since upload (days): 2280<br><br>Target audience: Professional	Source: Hospital/university<br><br>**Video Source Reliability (JAMA): 3**	Views (n.62)<br>Likes (n.0)<br>Dislikes (n.0)<br>Comments (disabled)<br>Subscriptions (616)<br><br>Like Ratio: 0<br>View Ratio: 2.72<br><br>**VPI: 0**	Information flow (4)<br>Information accuracy (5)<br>Quality (3)<br>Sensitivity (4)<br><br>**VIQI: 16**	Route of transmission (1)<br>HPV risk factors (1)<br>HPV screening (1)<br>Genotypes (1)<br>Oncogenic role (1)<br><br>Skin lesions (benign) (1)<br>Mucosal lesions (benign) (0)<br>Oral lesions (benign) (0)<br><br>Genital cancer Fx (1)<br>Genital cancer Mx (1)	Age (0)<br>Gender (0)<br>Type of vaccines (0)<br>HPV vaccine safety (0)<br><br>HPV vaccination advice:<br>Encouraging (1)<br>Discouraging (0)<br>Neutral (0)<br><br>Fake news (0)	Vaccination course (0)<br>Projections of genital cancer Fx (0)<br>Projections of genital cancer Mx (0)<br>Projection of OSCC (0)<br><br>**Content on Epidemiology Score: 0**<br><br>**VIDEO TOTAL-CONTENT SCORE: 10**

				OSCC (1)<br><br>**Content on HPV infection and related lesions Score: 9**	**Content on HPV vaccination Score: 1**	**GQS: 2**<br>**Video Educational Value:** very low/low
Link: https://www.youtube.com/watch?v=th8GXxQpe6U<br><br>Lenght (min): 9.27<br><br>Time elapsed since upload (days): 4142<br><br>Target audience: Professional	Source: Other<br><br>**Video Source Reliability (JAMA): 3**	Views (n.15405)<br>Likes (n.96)<br>Dislikes (n.20)<br>Comments (n.48)<br>Subscriptions (n.6140)<br><br>Like Ratio: 0.75<br>View Ratio: 371.92<br><br>**VPI: 2.79**	Information flow (3)<br>Information accuracy (3)<br>Quality (2)<br>Sensitivity (4)<br><br>**VIQI: 12**	Route of transmission (0)<br>HPV risk factors (0)<br>HPV screening (1)<br>Genotypes (1)<br>Oncogenic role (1)<br><br>Skin lesions (benign) (1)<br>Mucosal lesions (benign) (1)<br>Oral lesions (benign) (0)<br><br>Genital cancer Fx (1)<br>Genital cancer Mx (1)<br>OSCC (1)<br><br>**Content on HPV infection and related lesions Score: 8**	Age (1)<br>Gender (1)<br>Type of vaccines (1)<br>HPV vaccine safety (0)<br><br>HPV vaccination advice:<br>Encouraging (0)<br>Discouraging (0)<br>Neutral (1)<br><br>Fake news (0)<br><br>**Content on HPV vaccination Score: 4**	Vaccination course (0)<br>Projections of genital cancer Fx (1)<br>Projections of genital cancer Mx (0)<br>Projection of OSCC (0)<br><br>**Content on Epidemiology Score: 1**<br><br>**VIDEO TOTAL-CONTENT SCORE: 13**<br><br>**GQS: 2**<br>**Video Educational Value:** very low/low
Link: https://www.youtube.com/watch?app=desktop&v=sNjoCe_dHj8<br><br>Lenght (min): 4.45<br><br>Time elapsed since upload (days): 185<br><br>Target audience: Laypersons	Source: Other<br><br>**Video Source Reliability (JAMA): 3**	Views (n.279)<br>Likes (n.10)<br>Dislikes (n.0)<br>Comments (n.10)<br>Subscriptions (n.42500)<br><br>Like Ratio: 3.58<br>View Ratio: 150.81<br><br>**VPI: 5.39**	Information flow (2)<br>Information accuracy (2)<br>Quality (1)<br>Sensitivity (3)<br><br>**VIQI: 8**	Route of transmission (1)<br>HPV risk factors (0)<br>HPV screening (0)<br>Genotypes (0)<br>Oncogenic role (1)<br><br>Skin lesions (benign) (0)<br>Mucosal lesions (benign) (0)<br>Oral lesions (benign) (0)<br><br>Genital cancer Fx (1)<br>Genital cancer Mx (0)<br>OSCC (0)<br><br>**Content on HPV infection and related lesions Score: 3**	Age (1)<br>Gender (0)<br>Type of vaccines (0)<br>HPV vaccine safety (1)<br><br>HPV vaccination advice:<br>Encouraging (1)<br>Discouraging (0)<br>Neutral (0)<br><br>Fake news (0)<br><br>**Content on HPV vaccination Score: 3**	Vaccination course (1)<br>Projections of genital cancer Fx (1)<br>Projections of genital cancer Mx (0)<br>Projection of OSCC (0)<br><br>**Content on Epidemiology Score: 2**<br><br>**VIDEO TOTAL-CONTENT SCORE: 8**<br><br>**GQS: 2**<br>**Video Educational Value:** very low/low
Link: https://www.youtube.com/watch?v=BZZnVCrSeJY<br><br>Lenght (min): 7.42	Source: Hospital/university	Views (n.10159)<br>Likes (n.49)<br>Dislikes (n.5)	Information flow (5)	Route of transmission (0)<br>HPV risk factors (0)<br>HPV screening (1)	Age (0)<br>Gender (1)<br>Type of vaccines (0)	Vaccination course (0)<br>Projections of genital cancer Fx (0)

Time elapsed since upload (days): 2268<br><br>Target audience: Laypersons	**Video Source Reliability (JAMA): 2**	Comments (n.111)<br>Subscriptions (n.38700)<br><br>Like Ratio: 0.53<br>View Ratio: 447.93<br><br>**VPI: 2.37**	Information accuracy (2)<br>Quality (2)<br>Sensitivity (4)<br><br>**VIQI: 13**	Genotypes (0)<br>Oncogenic role (1)<br><br>Skin lesions (benign) (0)<br>Mucosal lesions (benign) (0)<br>Oral lesions (benign) (0)<br><br>Genital cancer Fx (1)<br>Genital cancer Mx (1)<br>OSCC (1)<br><br>**Content on HPV infection and related lesions Score: 5**	HPV vaccine safety (0)<br><br>HPV vaccination advice:<br>Encouraging (1)<br>Discouraging (0)<br>Neutral (0)<br><br>Fake news (0)<br><br>**Content on HPV vaccination Score: 2**	Projections of genital cancer Mx (0)<br>Projection of OSCC (0)<br><br>**Content on Epidemiology Score: 0**<br><br>**VIDEO TOTAL-CONTENT SCORE: 7**<br><br>**GQS: 1**<br>**Video Educational Value:** very low/low
Link: https://www.youtube.com/watch?v=ZzF1260OwjQ<br><br>Lenght (min): 11.35<br><br>Time elapsed since upload (days): 351<br><br>Target audience: Laypersons	Source: Hospital/university<br><br>**Video Source Reliability (JAMA): 3**	Views (n.112)<br>Likes (n.2)<br>Dislikes (n.0)<br>Comments (n.4)<br>Subscriptions (n.10100)<br><br>Like Ratio: 1.79<br>View Ratio: 31.91<br><br>**VPI: 0.57**	Information flow (5)<br>Information accuracy (5)<br>Quality (2)<br>Sensitivity (5)<br><br>**VIQI: 17**	Route of transmission (1)<br>HPV risk factors (1)<br>HPV screening (1)<br>Genotypes (1)<br>Oncogenic role (1)<br><br>Skin lesions (benign) (0)<br>Mucosal lesions (benign) (0)<br>Oral lesions (benign) (0)<br><br>Genital cancer Fx (1)<br>Genital cancer Mx (1)<br>OSCC (1)<br><br>**Content on HPV infection and related lesions Score: 8**	Age (1)<br>Gender (1)<br>Type of vaccines (1)<br>HPV vaccine safety (1)<br><br>HPV vaccination advice:<br>Encouraging (1)<br>Discouraging (0)<br>Neutral (0)<br><br>Fake news (0)<br><br>**Content on HPV vaccination Score: 5**	Vaccination course (0)<br>Projections of genital cancer Fx (0)<br>Projections of genital cancer Mx (0)<br>Projection of OSCC (0)<br><br>**Content on Epidemiology Score: 0**<br><br>**VIDEO TOTAL-CONTENT SCORE: 13**<br><br>**GQS: 4**<br>**Video Educational Value:** medium/good/excellent
Link: https://www.youtube.com/watch?v=R3OWs8rNtS8<br><br>Lenght (min): 14.56<br><br>Time elapsed since upload (days): 200<br><br>Target audience: Both	Source: Other<br><br>**Video Source Reliability (JAMA): 3**	Views (n.973)<br>Likes (n.16)<br>Dislikes (n.0)<br>Comments (n.2)<br>Subscriptions (n.6060000)<br><br>Like Ratio: 1.64<br>View Ratio: 486.50<br><br>**VPI: 7.98**	Information flow (5)<br>Information accuracy (4)<br>Quality (1)<br>Sensitivity (5)<br><br>**VIQI: 15**	Route of transmission (1)<br>HPV risk factors (1)<br>HPV screening (1)<br>Genotypes (1)<br>Oncogenic role (1)<br><br>Skin lesions (benign) (1)<br>Mucosal lesions (benign) (0)<br>Oral lesions (benign) (0)	Age (1)<br>Gender (1)<br>Type of vaccines (1)<br>HPV vaccine safety (1)<br><br>HPV vaccination advice:<br>Encouraging (0)<br>Discouraging (0)<br>Neutral (1)<br><br>Fake news (0)	Vaccination course (1)<br>Projections of genital cancer Fx (0)<br>Projections of genital cancer Mx (0)<br>Projection of OSCC (0)<br><br>**Content on Epidemiology Score: 1**

				Genital cancer Fx (1)<br>Genital cancer Mx (1)<br>OSCC (1)<br><br>**Content on HPV infection and related lesions Score: 9**	**Content on HPV vaccination Score: 4**	**VIDEO TOTAL-CONTENT SCORE: 15**<br><br>**GQS: 5**<br>**Video Educational Value:**<br>medium/good/excellent
Link: https://www.youtube.com/watch?app=desktop&v=DsYrpfC7HBo<br><br>Lenght (min): 13.13<br><br>Time elapsed since upload (days): 118<br><br>Target audience: Both	Source: Hospital/university<br><br>**Video Source Reliability (JAMA): 3**	Views (n.350)<br>Likes (n.17)<br>Dislikes (n.0)<br>Comments (n.91)<br>Subscriptions (n.41400)<br><br>Like Ratio: 4.85<br>View Ratio: 296.61<br><br>**VPI: 14.38**	Information flow (4)<br>Information accuracy (4)<br>Quality (5)<br>Sensitivity (3)<br><br>**VIQI: 16**	Route of transmission (1)<br>HPV risk factors (1)<br>HPV screening (1)<br>Genotypes (1)<br>Oncogenic role (1)<br><br>Skin lesions (benign) (0)<br>Mucosal lesions (benign) (0)<br>Oral lesions (benign) (0)<br><br>Genital cancer Fx (1)<br>Genital cancer Mx (1)<br>OSCC (1)<br><br>**Content on HPV infection and related lesions Score: 8**	Age (1)<br>Gender (0)<br>Type of vaccines (0)<br>HPV vaccine safety (1)<br><br>HPV vaccination advice:<br>Encouraging (1)<br>Discouraging (0)<br>Neutral (0)<br><br>Fake news (0)<br><br>**Content on HPV vaccination Score: 3**	Vaccination course (1)<br>Projections of genital cancer Fx (1)<br>Projections of genital cancer Mx (1)<br>Projection of OSCC (1)<br><br>**Content on Epidemiology Score: 4**<br><br>**VIDEO TOTAL-CONTENT SCORE: 15**<br><br>**GQS: 4**<br>**Video Educational Value:**<br>medium/good/excellent
Link: https://www.youtube.com/watch?v=XI2Jp9x6zWc<br><br>Lenght (min): 7.02<br><br>Time elapsed since upload (days): 623<br><br>Target audience: Laypersons	Source: Other<br><br>**Video Source Reliability (JAMA): 3**	Views (n.90)<br>Likes (n.0)<br>Dislikes (n.0)<br>Comments (n.0)<br>Subscriptions (n.22100)<br><br>Like Ratio: 0<br>View Ratio: 14.45<br><br>**VPI: 0**	Information flow (5)<br>Information accuracy (4)<br>Quality (3)<br>Sensitivity (4)<br><br>**VIQI: 16**	Route of transmission (0)<br>HPV risk factors (0)<br>HPV screening (0)<br>Genotypes (1)<br>Oncogenic role (1)<br><br>Skin lesions (benign) (0)<br>Mucosal lesions (benign) (0)<br>Oral lesions (benign) (0)<br><br>Genital cancer Fx (1)<br>Genital cancer Mx (1)<br>OSCC (1)<br><br>**Content on HPV infection and related lesions Score: 5**	Age (1)<br>Gender (1)<br>Type of vaccines (1)<br>HPV vaccine safety (1)<br><br>HPV vaccination advice:<br>Encouraging (1)<br>Discouraging (0)<br>Neutral (0)<br><br>Fake news (0)<br><br>**Content on HPV vaccination Score: 5**	Vaccination course (0)<br>Projections of genital cancer Fx (0)<br>Projections of genital cancer Mx (0)<br>Projection of OSCC (0)<br><br>**Content on Epidemiology Score: 0**<br><br>**VIDEO TOTAL-CONTENT SCORE: 10**<br><br>**GQS: 4**<br>**Video Educational Value:**<br>medium/good/excellent

Link: https://www.youtube.com/watch?v=6E9-pzjam6Y<br><br>Lenght (min): 12.17<br><br>Time elapsed since upload (days): 2280<br><br>Target audience: Professional	Source: Hospital/university<br><br>**Video Source Reliability (JAMA): 4**	Views (n.77)<br>Likes (n.0)<br>Dislikes (n.0)<br>Comments (disabled)<br>Subscriptions (616)<br><br>Like Ratio: 0<br>View Ratio: 3.38<br><br>**VPI: 0**	Information flow (4)<br>Information accuracy (4)<br>Quality (3)<br>Sensitivity (4)<br><br>**VIQI: 15**	Route of transmission (1)<br>HPV risk factors (0)<br>HPV screening (0)<br>Genotypes (0)<br>Oncogenic role (1)<br><br>Skin lesions (benign) (1)<br>Mucosal lesions (benign) (1)<br>Oral lesions (benign) (0)<br><br>Genital cancer Fx (1)<br>Genital cancer Mx (1)<br>OSCC (1)<br><br>**Content on HPV infection and related lesions Score: 7**	Age (1)<br>Gender (1)<br>Type of vaccines (1)<br>HPV vaccine safety (0)<br><br>HPV vaccination advice:<br>Encouraging (1)<br>Discouraging (0)<br>Neutral (0)<br><br>Fake news (0)<br><br>**Content on HPV vaccination Score: 4**	Vaccination course (1)<br>Projections of genital cancer Fx (1)<br>Projections of genital cancer Mx (0)<br>Projection of OSCC (0)<br><br>**Content on Epidemiology Score: 2**<br><br>**VIDEO TOTAL-CONTENT SCORE: 13**<br><br>**GQS: 3**<br>**Video Educational Value:** medium/good/excellent
Link: https://www.youtube.com/watch?v=qs7sTx5lPo4<br><br>Lenght (min): 5.49<br><br>Time elapsed since upload (days): 567<br><br>Target audience: Professional	Source: Other<br><br>**Video Source Reliability (JAMA): 3**	Views (n.24)<br>Likes (n.0)<br>Dislikes (n.0)<br>Comments (disabled)<br>Subscriptions (411)<br><br>Like Ratio: 0<br>View Ratio: 4.23<br><br>**VPI: 0**	Information flow (2)<br>Information accuracy (5)<br>Quality (4)<br>Sensitivity (4)<br><br>**VIQI: 15**	Route of transmission (0)<br>HPV risk factors (0)<br>HPV screening (0)<br>Genotypes (0)<br>Oncogenic role (0)<br><br>Skin lesions (benign) (0)<br>Mucosal lesions (benign) (0)<br>Oral lesions (benign) (0)<br><br>Genital cancer Fx (0)<br>Genital cancer Mx (0)<br>OSCC (0)<br><br>**Content on HPV infection and related lesions Score: 0**	Age (0)<br>Gender (1)<br>Type of vaccines (0)<br>HPV vaccine safety (1)<br><br>HPV vaccination advice:<br>Encouraging (0)<br>Discouraging (0)<br>Neutral (1)<br><br>Fake news (0)<br><br>**Content on HPV vaccination Score: 3**	Vaccination course (1)<br>Projections of genital cancer Fx (0)<br>Projections of genital cancer Mx (0)<br>Projection of OSCC (0)<br><br>**Content on Epidemiology Score: 1**<br><br>**VIDEO TOTAL-CONTENT SCORE: 4**<br><br>**GQS: 1**<br>**Video Educational Value:** very low/low
Link: https://www.youtube.com/watch?v=E5CqSM8GnFc<br><br>Lenght (min): 7.33<br><br>Time elapsed since upload (days): 5<br><br>Target audience: Laypersons	Source: Other<br><br>**Video Source Reliability (JAMA): 4**	Views (n.9)<br>Likes (n.0)<br>Dislikes (n.0)<br>Comments (n.0)<br>Subscriptions (n.66)<br><br>Like Ratio: 0<br>View Ratio: 180	Information flow (4)<br>Information accuracy (4)<br>Quality (4)<br>Sensitivity (4)<br><br>**VIQI: 16**	Route of transmission (1)<br>HPV risk factors (1)<br>HPV screening (0)<br>Genotypes (1)<br>Oncogenic role (1)<br><br>Skin lesions (benign) (0)	Age (1)<br>Gender (1)<br>Type of vaccines (1)<br>HPV vaccine safety (1)<br><br>HPV vaccination advice:<br>Encouraging (1)<br>Discouraging (0)	Vaccination course (0)<br>Projections of genital cancer Fx (0)<br>Projections of genital cancer Mx (0)<br>Projection of OSCC (0)

		**VPI: 0**		Mucosal lesions (benign) (0)<br>Oral lesions (benign) (0)<br><br>Genital cancer Fx (1)<br>Genital cancer Mx (1)<br>OSCC (1)<br><br>**Content on HPV infection and related lesions Score: 7**	Neutral (0)<br><br>Fake news (0)<br><br>**Content on HPV vaccination Score: 5**	**Content on Epidemiology Score: 0**<br><br>**VIDEO TOTAL-CONTENT SCORE: 12**<br><br>**GQS: 4**<br>**Video Educational Value:** medium/good/excellent
Link: https://www.youtube.com/watch?v=Dt9MG5SwjYg<br><br>Lenght (min): 11.11<br><br>Time elapsed since upload (days): 340<br><br>Target audience: Laypersons	Source: Other healthcare provider (Obstetrician-gynecologist)<br><br>**Video Source Reliability (JAMA): 3**	Views (n.20940)<br>Likes (n.950)<br>Dislikes (n.12)<br>Comments (n.173)<br>Subscriptions (n.666000)<br><br>Like Ratio: 4.59<br>View Ratio: 6158.82<br><br>**VPI: 282.69**	Information flow (5)<br>Information accuracy (5)<br>Quality (5)<br>Sensitivity (4)<br><br>**VIQI: 19**	Route of transmission (1)<br>HPV risk factors (0)<br>HPV screening (1)<br>Genotypes (1)<br>Oncogenic role (1)<br><br>Skin lesions (benign) (0)<br>Mucosal lesions (benign) (1)<br>Oral lesions (benign) (0)<br><br>Genital cancer Fx (1)<br>Genital cancer Mx (1)<br>OSCC (1)<br><br>**Content on HPV infection and related lesions Score: 8**	Age (1)<br>Gender (1)<br>Type of vaccines (1)<br>HPV vaccine safety (1)<br><br>HPV vaccination advice:<br>Encouraging (1)<br>Discouraging (0)<br>Neutral (0)<br><br>Fake news (0)<br><br>**Content on HPV vaccination Score: 5**	Vaccination course (0)<br>Projections of genital cancer Fx (0)<br>Projections of genital cancer Mx (0)<br>Projection of OSCC (0)<br><br>**Content on Epidemiology Score: 0**<br><br>**VIDEO TOTAL-CONTENT SCORE: 13**<br><br>**GQS: 4**<br>**Video Educational Value:** medium/good/excellent
Link: https://www.youtube.com/watch?v=b-FU3RatRKQ<br><br>Lenght (min): 4.49<br><br>Time elapsed since upload (days): 823<br><br>Target audience: Layprsons	Source: Other<br><br>**Video Source Reliability (JAMA): 2**	Views (n.609)<br>Likes (n.2)<br>Dislikes (n.0)<br>Comments (n.10)<br>Subscriptions (n.3340)<br><br>Like Ratio: 0.33<br>View Ratio: 74.00<br><br>**VPI: 24.42**	Information flow (3)<br>Information accuracy (2)<br>Quality (1)<br>Sensitivity (2)<br><br>**VIQI: 8**	Route of transmission (0)<br>HPV risk factors (0)<br>HPV screening (0)<br>Genotypes (0)<br>Oncogenic role (1)<br><br>Skin lesions (benign) (0)<br>Mucosal lesions (benign) (0)<br>Oral lesions (benign) (0)<br><br>Genital cancer Fx (1)<br>Genital cancer Mx (0)<br>OSCC (0)	Age (1)<br>Gender (0)<br>Type of vaccines (1)<br>HPV vaccine safety (1)<br><br>HPV vaccination advice:<br>Encouraging (0)<br>Discouraging (0)<br>Neutral (1)<br><br>Fake news (0)<br><br>**Content on HPV vaccination Score: 4**	Vaccination course (0)<br>Projections of genital cancer Fx (0)<br>Projections of genital cancer Mx (0)<br>Projection of OSCC (0)<br><br>**Content on Epidemiology Score: 0**<br><br>**VIDEO TOTAL-CONTENT SCORE: 6**<br><br>**GQS: 2**

				**Content on HPV infection and related lesions Score: 2**		**Video Educational Value:** very low/low
Link: https://www.youtube.com/watch?v=2rkMRRTiNxM<br><br>Lenght (min): 4.33<br><br>Time elapsed since upload (days): 1548<br><br>Target audience: Laypersons	Source: Other<br><br>**Video Source Reliability (JAMA): 2**	Views (n.96)<br>Likes (n.2)<br>Dislikes (n.0)<br>Comments (n.3)<br>Subscriptions (n.147000)<br><br>Like Ratio: 2.08<br>View Ratio: 6.20<br><br>**VPI: 0.13**	Information flow (4)<br>Information accuracy (3)<br>Quality (3)<br>Sensitivity (4)<br><br>**VIQI: 14**	Route of transmission (1)<br>HPV risk factors (1)<br>HPV screening (1)<br>Genotypes (1)<br>Oncogenic role (1)<br><br>Skin lesions (benign) (0)<br>Mucosal lesions (benign) (0)<br>Oral lesions (benign) (0)<br><br>Genital cancer Fx (1)<br>Genital cancer Mx (1)<br>OSCC (1)<br><br>**Content on HPV infection and related lesions Score: 8**	Age (1)<br>Gender (1)<br>Type of vaccines (0)<br>HPV vaccine safety (1)<br><br>HPV vaccination advice:<br>Encouraging (1)<br>Discouraging (0)<br>Neutral (0)<br><br>Fake news (0)<br><br>**Content on HPV vaccination Score: 4**	Vaccination course (0)<br>Projections of genital cancer Fx (0)<br>Projections of genital cancer Mx (0)<br>Projection of OSCC (0)<br><br>**Content on Epidemiology Score: 0**<br><br>**VIDEO TOTAL-CONTENT SCORE: 12**<br><br>**GQS: 3**<br>**Video Educational Value:** medium/good/excellent
Link: https://www.youtube.com/watch?v=wkaTMob863w<br><br>Lenght (min): 10.18<br><br>Time elapsed since upload (days): 42<br><br>Target audience: Professional	Source: Other<br><br>**Video Source Reliability (JAMA): 3**	Views (n.18)<br>Likes (n.1)<br>Dislikes (n.0)<br>Comments (disabled)<br>Subscriptions (411)<br><br>Like Ratio: 5.56<br>View Ratio: 42.86<br><br>**VPI: 2.38**	Information flow (2)<br>Information accuracy (3)<br>Quality (2)<br>Sensitivity (4)<br><br>**VIQI: 11**	Route of transmission (0)<br>HPV risk factors (0)<br>HPV screening (1)<br>Genotypes (1)<br>Oncogenic role (1)<br><br>Skin lesions (benign) (0)<br>Mucosal lesions (benign) (0)<br>Oral lesions (benign) (0)<br><br>Genital cancer Fx (1)<br>Genital cancer Mx (1)<br>OSCC (0)<br><br>**Content on HPV infection and related lesions Score: 5**	Age (1)<br>Gender (1)<br>Type of vaccines (1)<br>HPV vaccine safety (1)<br><br>HPV vaccination advice:<br>Encouraging (0)<br>Discouraging (0)<br>Neutral (1)<br><br>Fake news (0)<br><br>**Content on HPV vaccination Score: 5**	Vaccination course (0)<br>Projections of genital cancer Fx (0)<br>Projections of genital cancer Mx (0)<br>Projection of OSCC (0)<br><br>**Content on Epidemiology Score: 0**<br><br>**VIDEO TOTAL-CONTENT SCORE: 10**<br><br>**GQS: 3**<br>**Video Educational Value:** medium/good/excellent
Link: https://www.youtube.com/watch?v=jVZLh7Ufu08<br><br>Lenght (min):6.33<br><br>Time elapsed since upload (days): 2650	Source: Other<br><br>**Video Source Reliability (JAMA): 3**	Views (n.2643)<br>Likes (n.12)<br>Dislikes (n.2)<br>Comments (disabled)	Information flow (3)<br>Information accuracy (3)<br>Quality (2)	Route of transmission (0)<br>HPV risk factors (0)<br>HPV screening (0)<br>Genotypes (0)<br>Oncogenic role (1)	Age (1)<br>Gender (1)<br>Type of vaccines (0)<br>HPV vaccine safety (1)	Vaccination course (0)<br>Projections of genital cancer Fx (0)<br>Projections of genital cancer Mx (0)

Target audience: Laypersons		Subscriptions (n.7230)<br><br>Like Ratio: 0.53<br>View Ratio: 99.73<br><br>**VPI: 0.53**	Sensitivity (3)<br><br>**VIQI: 11**	Skin lesions (benign) (1)<br>Mucosal lesions (benign) (1)<br>Oral lesions (benign) (0)<br><br>Genital cancer Fx (1)<br>Genital cancer Mx (1)<br>OSCC (0)<br><br>**Content on HPV infection and related lesions Score: 5**	HPV vaccination advice:<br>Encouraging (1)<br>Discouraging (0)<br>Neutral (0)<br><br>Fake news (0)<br><br>**Content on HPV vaccination Score: 4**	Projection of OSCC (0)<br><br>**Content on Epidemiology Score: 0**<br><br>**VIDEO TOTAL-CONTENT SCORE: 9**<br><br>**GQS: 2**<br>**Video Educational Value:** very low/low
Link: https://www.youtube.com/watch?v=Zqc-WbOOfbk<br><br>Lenght (min): 5.30<br><br>Time elapsed since upload (days): 1792<br><br>Target audience: Layperons	Source: Other<br><br>**Video Source Reliability (JAMA): 1**	Views (n.311)<br>Likes (n.0)<br>Dislikes (n.0)<br>Comments (disabled)<br>Subscriptions (20700)<br><br>Like Ratio: 0<br>View Ratio: 17.35<br><br>**VPI: 0**	Information flow (4)<br>Information accuracy (1)<br>Quality (4)<br>Sensitivity (2)<br><br>**VIQI: 11**	Route of transmission (0)<br>HPV risk factors (1)<br>HPV screening (0)<br>Genotypes (0)<br>Oncogenic role (0)<br><br>Skin lesions (benign) (0)<br>Mucosal lesions (benign) (0)<br>Oral lesions (benign) (0)<br><br>Genital cancer Fx (1)<br>Genital cancer Mx (0)<br>OSCC (0)<br><br>**Content on HPV infection and related lesions Score: 2**	Age (1)<br>Gender (1)<br>Type of vaccines (0)<br>HPV vaccine safety (0)<br><br>HPV vaccination advice:<br>Encouraging (0)<br>Discouraging (0)<br>Neutral (1)<br><br>Fake news (0)<br><br>**Content on HPV vaccination Score: 3**	Vaccination course (1)<br>Projections of genital cancer Fx (0)<br>Projections of genital cancer Mx (0)<br>Projection of OSCC (0)<br><br>**Content on Epidemiology Score: 1**<br><br>**VIDEO TOTAL-CONTENT SCORE: 6**<br><br>**GQS: 1**<br>**Video Educational Value:** very low/low
Link: https://www.youtube.com/watch?v=hjMpju3XlOM<br><br>Lenght (min): 7.18<br><br>Time elapsed since upload (days): 481<br><br>Target audience: Laypersons	Source: Other<br><br>**Video Source Reliability (JAMA): 4**	Views (n.318)<br>Likes (n.2)<br>Dislikes (n.5)<br>Comments (n.11)<br>Subscriptions (n.17400)<br><br>Like Ratio: 2.20<br>View Ratio: 66.11<br><br>**VPI: 1.45**	Information flow (4)<br>Information accuracy (4)<br>Quality (3)<br>Sensitivity (4)<br><br>**VIQI: 15**	Route of transmission (1)<br>HPV risk factors (0)<br>HPV screening (1)<br>Genotypes (1)<br>Oncogenic role (1)<br><br>Skin lesions (benign) (0)<br>Mucosal lesions (benign) (1)<br>Oral lesions (benign) (0)<br><br>Genital cancer Fx (1)<br>Genital cancer Mx (1)	Age (1)<br>Gender (1)<br>Type of vaccines (0)<br>HPV vaccine safety (0)<br><br>HPV vaccination advice:<br>Encouraging (1)<br>Discouraging (0)<br>Neutral (0)<br><br>Fake news (0)	Vaccination course (1)<br>Projections of genital cancer Fx (1)<br>Projections of genital cancer Mx (0)<br>Projection of OSCC (0)<br><br>**Content on Epidemiology Score: 2**<br><br>**VIDEO TOTAL-CONTENT SCORE: 13**

				OSCC (1)<br><br>**Content on HPV infection and related lesions Score: 8**	**Content on HPV vaccination Score: 3**	**GQS: 3**<br>**Video Educational Value:** medium/good/excellent
Link: https://www.youtube.com/watch?v=VTjgQ_8c_zs<br><br>Lenght (min): 4.16<br><br>Time elapsed since upload (days): 2712<br><br>Target audience: Professional	Source: Other<br><br>**Video Source Reliability (JAMA): 3**	Views (n.14817)<br>Likes (n.541)<br>Dislikes (n.5)<br>Comments (n.52)<br>Subscriptions (n.441000)<br><br>Like Ratio: 3.68<br>View Ratio: 546.35<br><br>**VPI: 20.11**	Information flow (4)<br>Information accuracy (5)<br>Quality (4)<br>Sensitivity (4)<br><br>**VIQI: 17**	Route of transmission (0)<br>HPV risk factors (0)<br>HPV screening (0)<br>Genotypes (0)<br>Oncogenic role (0)<br><br>Skin lesions (benign) (0)<br>Mucosal lesions (benign) (0)<br>Oral lesions (benign) (0)<br><br>Genital cancer Fx (0)<br>Genital cancer Mx (0)<br>OSCC (0)<br><br>**Content on HPV infection and related lesions Score: 0**	Age (1)<br>Gender (1)<br>Type of vaccines (0)<br>HPV vaccine safety (1)<br><br>HPV vaccination advice:<br>Encouraging (0)<br>Discouraging (0)<br>Neutral (1)<br><br>Fake news (0)<br><br>**Content on HPV vaccination Score: 4**	Vaccination course (1)<br>Projections of genital cancer Fx (0)<br>Projections of genital cancer Mx (0)<br>Projection of OSCC (0)<br><br>**Content on Epidemiology Score: 1**<br><br>**VIDEO TOTAL-CONTENT SCORE: 5**<br><br>**GQS: 2**<br>**Video Educational Value:** very low/low
Link: https://www.youtube.com/watch?v=4UYyeRFUP1I<br><br>Lenght (min): 5.39<br><br>Time elapsed since upload (days): 1699<br><br>Target audience: Professional	Source: Hospital/university<br><br>**Video Source Reliability (JAMA): 4**	Views (n.3096)<br>Likes (n.17)<br>Dislikes (n.38)<br>Comments (n.25)<br>Subscriptions (n.1930)<br><br>Like Ratio: 1.78<br>View Ratio: 182.22<br><br>**VPI: 3.24**	Information flow (3)<br>Information accuracy (2)<br>Quality (2)<br>Sensitivity (3)<br><br>**VIQI: 10**	Route of transmission (1)<br>HPV risk factors (0)<br>HPV screening (0)<br>Genotypes (1)<br>Oncogenic role (1)<br><br>Skin lesions (benign) (0)<br>Mucosal lesions (benign) (1)<br>Oral lesions (benign) (0)<br><br>Genital cancer Fx (1)<br>Genital cancer Mx (1)<br>OSCC (1)<br><br>**Content on HPV infection and related lesions Score: 7**	Age (0)<br>Gender (0)<br>Type of vaccines (0)<br>HPV vaccine safety (1)<br><br>HPV vaccination advice:<br>Encouraging (1)<br>Discouraging (0)<br>Neutral (0)<br><br>Fake news (0)<br><br>**Content on HPV vaccination Score: 2**	Vaccination course (1)<br>Projections of genital cancer Fx (1)<br>Projections of genital cancer Mx (1)<br>Projection of OSCC (1)<br><br>**Content on Epidemiology Score: 4**<br><br>**VIDEO TOTAL-CONTENT SCORE: 13**<br><br>**GQS: 3**<br>**Video Educational Value:** medium/good/excellent
Link: https://www.youtube.com/watch?v=nh2p7XJWCik<br><br>Lenght (min): 4.39	Source: Other	Views (n.630)<br>Likes (n.0)<br>Dislikes (n.0)	Information flow (4)	Route of transmission (1)<br>HPV risk factors (1)<br>HPV screening (1)	Age (1)<br>Gender (1)<br>Type of vaccines (0)	Vaccination course (0)<br>Projections of genital cancer Fx (0)

Time elapsed since upload (days): 1453<br><br>Target audience: Laypersons	**Video Source Reliability (JAMA): 2**	Comments (disabled)<br>Subscriptions (15800)<br><br>Like Ratio: 0<br>View Ratio: 43.36<br><br>**VPI: 0**	Information accuracy (4)<br>Quality (1)<br>Sensitivity (4)<br><br>**VIQI: 13**	Genotypes (1)<br>Oncogenic role (1)<br><br>Skin lesions (benign) (0)<br>Mucosal lesions (benign) (1)<br>Oral lesions (benign) (0)<br><br>Genital cancer Fx (1)<br>Genital cancer Mx (1)<br>OSCC (0)<br><br>**Content on HPV infection and related lesions Score: 8**	HPV vaccine safety (1)<br><br>HPV vaccination advice:<br>Encouraging (0)<br>Discouraging (0)<br>Neutral (1)<br><br>Fake news (0)<br><br>**Content on HPV vaccination Score: 4**	Projections of genital cancer Mx (0)<br>Projection of OSCC (0)<br><br>**Content on Epidemiology Score: 0**<br><br>**VIDEO TOTAL-CONTENT SCORE: 12**<br><br>**GQS: 3**<br>**Video Educational Value:** medium/good/excellent
Link: https://www.youtube.com/watch?v=rC6rNcSacgQ<br><br>Lenght (min): 15.08<br><br>Time elapsed since upload (days): 7<br><br>Target audience: Professional	Source: Other<br><br>**Video Source Reliability (JAMA): 3**	Views (n.7)<br>Likes (n.0)<br>Dislikes (n.0)<br>Comments (disabled)<br>Subscriptions (411)<br><br>Like Ratio: 0<br>View Ratio: 100<br><br>**VPI: 0**	Information flow (5)<br>Information accuracy (5)<br>Quality (5)<br>Sensitivity (5)<br><br>**VIQI: 20**	Route of transmission (1)<br>HPV risk factors (1)<br>HPV screening (0)<br>Genotypes (1)<br>Oncogenic role (1)<br><br>Skin lesions (benign) (0)<br>Mucosal lesions (benign) (0)<br>Oral lesions (benign) (0)<br><br>Genital cancer Fx (1)<br>Genital cancer Mx (1)<br>OSCC (0)<br><br>**Content on HPV infection and related lesions Score: 6**	Age (1)<br>Gender (1)<br>Type of vaccines (1)<br>HPV vaccine safety (0)<br><br>HPV vaccination advice:<br>Encouraging (0)<br>Discouraging (0)<br>Neutral (1)<br><br>Fake news (0)<br><br>**Content on HPV vaccination Score: 4**	Vaccination course (1)<br>Projections of genital cancer Fx (1)<br>Projections of genital cancer Mx (0)<br>Projection of OSCC (0)<br><br>**Content on Epidemiology Score: 2**<br><br>**VIDEO TOTAL-CONTENT SCORE: 12**<br><br>**GQS: 4**<br>**Video Educational Value:** medium/good/excellent
Link: https://www.youtube.com/watch?app=desktop&v=GEczA7pY71o<br><br>Lenght (min): 6.05<br><br>Time elapsed since upload (days): 203<br><br>Target audience: Both	Source: Hospital/university<br><br>**Video Source Reliability (JAMA): 3**	Views (n.623)<br>Likes (disabled)<br>Dislikes (disabled)<br>Comments (disabled)<br>Subscriptions (19000)<br><br>Like Ratio: Not evaluable<br>View Ratio: 306.90	Information flow (3)<br>Information accuracy (2)<br>Quality (2)<br>Sensitivity (3)<br><br>**VIQI: 10**	Route of transmission (1)<br>HPV risk factors (0)<br>HPV screening (1)<br>Genotypes (0)<br>Oncogenic role (1)<br><br>Skin lesions (benign) (0)<br>Mucosal lesions (benign) (0)<br>Oral lesions (benign) (0)	Age (1)<br>Gender (0)<br>Type of vaccines (1)<br>HPV vaccine safety (1)<br><br>HPV vaccination advice:<br>Encouraging (1)<br>Discouraging (0)<br>Neutral (0)<br><br>Fake news (0)	Vaccination course (0)<br>Projections of genital cancer Fx (0)<br>Projections of genital cancer Mx (0)<br>Projection of OSCC (0)<br><br>**Content on Epidemiology Score: 0**

		**VPI: Not evaluable**		Genital cancer Fx (1)<br>Genital cancer Mx (1)<br>OSCC (0)<br><br>**Content on HPV infection and related lesions Score: 5**	**Content on HPV vaccination Score: 4**	**VIDEO TOTAL-CONTENT SCORE: 9**<br><br>**GQS: 2**<br>**Video Educational Value:** very low/low
Link: https://www.youtube.com/watch?v=DMJ7EvP3Vi4<br><br>Lenght (min): 7.56<br><br>Time elapsed since upload (days): 1398<br><br>Target audience: Professional	Source: Other<br><br>**Video Source Reliability (JAMA): 3**	Views (n.307)<br>Likes (n.3)<br>Dislikes (n.0)<br>Comments (n.1)<br>Subscriptions (n.2000)<br><br>Like Ratio: 0.98<br>View Ratio: 21.96<br><br>**VPI: 0.21**	Information flow (3)<br>Information accuracy (2)<br>Quality (1)<br>Sensitivity (4)<br><br>**VIQI: 10**	Route of transmission (0)<br>HPV risk factors (0)<br>HPV screening (0)<br>Genotypes (0)<br>Oncogenic role (1)<br><br>Skin lesions (benign) (0)<br>Mucosal lesions (benign) (0)<br>Oral lesions (benign) (0)<br><br>Genital cancer Fx (1)<br>Genital cancer Mx (1)<br>OSCC (0)<br><br>**Content on HPV infection and related lesions Score: 3**	Age (1)<br>Gender (1)<br>Type of vaccines (0)<br>HPV vaccine safety (1)<br><br>HPV vaccination advice:<br>Encouraging (1)<br>Discouraging (0)<br>Neutral (0)<br><br>Fake news (0)<br><br>**Content on HPV vaccination Score: 4**	Vaccination course (0)<br>Projections of genital cancer Fx (0)<br>Projections of genital cancer Mx (0)<br>Projection of OSCC (0)<br><br>**Content on Epidemiology Score: 0**<br><br>**VIDEO TOTAL-CONTENT SCORE: 7**<br><br>**GQS: 2**<br>**Video Educational Value:** very low/low
Link:<br>https://www.youtube.com/watch?app=desktop&v=3VmfMvtIt1g<br><br>Lenght (min): 5.14<br><br>Time elapsed since upload (days): 96<br><br>Target audience: Laypersons	Source: Other<br><br>**Video Source Reliability (JAMA): 3**	Views (n.16341)<br>Likes (n.419)<br>Dislikes (n.9)<br>Comments (n.119)<br>Subscriptions (n.350000)<br><br>Like Ratio: 2.61<br>View Ratio: 17021.87<br><br>**VPI: 444.27**	Information flow (3)<br>Information accuracy (3)<br>Quality (2)<br>Sensitivity (3)<br><br>**VIQI: 11**	Route of transmission (1)<br>HPV risk factors (1)<br>HPV screening (1)<br>Genotypes (0)<br>Oncogenic role (1)<br><br>Skin lesions (benign) (0)<br>Mucosal lesions (benign) (0)<br>Oral lesions (benign) (0)<br><br>Genital cancer Fx (1)<br>Genital cancer Mx (1)<br>OSCC (1)<br><br>**Content on HPV infection and related lesions Score: 7**	Age (1)<br>Gender (0)<br>Type of vaccines (1)<br>HPV vaccine safety (1)<br><br>HPV vaccination advice:<br>Encouraging (1)<br>Discouraging (0)<br>Neutral (0)<br><br>Fake news (0)<br><br>**Content on HPV vaccination Score: 4**	Vaccination course (1)<br>Projections of genital cancer Fx (0)<br>Projections of genital cancer Mx (0)<br>Projection of OSCC (0)<br><br>**Content on Epidemiology Score: 1**<br><br>**VIDEO TOTAL-CONTENT SCORE: 12**<br><br>**GQS: 3**<br>**Video Educational Value:**<br>medium/good/excellent

Link: https://www.youtube.com/watch?v=VXhMQDslQ_Y<br><br>Lenght (min): 4.23<br><br>Time elapsed since upload (days): 1048<br><br>Target audience: Both	Source: Other<br><br>**Video Source Reliability (JAMA): 2**	Views (n.318)<br>Likes (n.6)<br>Dislikes (n.0)<br>Comments (n.11)<br>Subscriptions (n.321000)<br><br>Like Ratio: 1.89<br>View Ratio: 30.34<br><br>**VPI: 0.57**	Information flow (3)<br>Information accuracy (2)<br>Quality (1)<br>Sensitivity (4)<br><br>**VIQI: 10**	Route of transmission (1)<br>HPV risk factors (0)<br>HPV screening (0)<br>Genotypes (1)<br>Oncogenic role (1)<br><br>Skin lesions (benign) (0)<br>Mucosal lesions (benign) (0)<br>Oral lesions (benign) (0)<br><br>Genital cancer Fx (1)<br>Genital cancer Mx (1)<br>OSCC (1)<br><br>**Content on HPV infection and related lesions Score: 6**	Age (1)<br>Gender (1)<br>Type of vaccines (1)<br>HPV vaccine safety (0)<br><br>HPV vaccination advice:<br>Encouraging (1)<br>Discouraging (0)<br>Neutral (0)<br><br>Fake news (0)<br><br>**Content on HPV vaccination Score: 4**	Vaccination course (0)<br>Projections of genital cancer Fx (0)<br>Projections of genital cancer Mx (0)<br>Projection of OSCC (0)<br><br>**Content on Epidemiology Score: 0**<br><br>**VIDEO TOTAL-CONTENT SCORE: 10**<br><br>**GQS: 2**<br>**Video Educational Value:** very low/low
Link:<br>https://www.youtube.com/watch?app=desktop&v=ducC8kRJDyw<br><br>Lenght (min): 7.47<br><br>Time elapsed since upload (days): 2590<br><br>Target audience: Laypersons	Source: Other healthcare provider (Pediatrician)<br><br>**Video Source Reliability (JAMA): 3**	Views (n.30641)<br>Likes (n.118)<br>Dislikes (n.25)<br>Comments (n.299)<br>Subscriptions (n.441000)<br><br>Like Ratio: 44.13<br>View Ratio: 1183.05<br><br>**VPI: 522.08**	Information flow (3)<br>Information accuracy (3)<br>Quality (3)<br>Sensitivity (3)<br><br>**VIQI: 12**	Route of transmission (1)<br>HPV risk factors (1)<br>HPV screening (0)<br>Genotypes (0)<br>Oncogenic role (1)<br><br>Skin lesions (benign) (1)<br>Mucosal lesions (benign) (0)<br>Oral lesions (benign) (0)<br><br>Genital cancer Fx (1)<br>Genital cancer Mx (1)<br>OSCC (0)<br><br>**Content on HPV infection and related lesions Score: 6**	Age (1)<br>Gender (0)<br>Type of vaccines (0)<br>HPV vaccine safety (1)<br><br>HPV vaccination advice:<br>Encouraging (1)<br>Discouraging (0)<br>Neutral (0)<br><br>Fake news (0)<br><br>**Content on HPV vaccination Score: 3**	Vaccination course (1)<br>Projections of genital cancer Fx (1)<br>Projections of genital cancer Mx (1)<br>Projection of OSCC (0)<br><br>**Content on Epidemiology Score: 3**<br><br>**VIDEO TOTAL-CONTENT SCORE: 12**<br><br>**GQS: 3**<br>**Video Educational Value:** medium/good/excellent
Link:<br>https://www.youtube.com/watch?app=desktop&v=AegRrnwGCB4<br><br>Lenght (min): 9.56<br><br>Time elapsed since upload (days): 224<br><br>Target audience: Laypersons	Source: Other<br><br>**Video Source Reliability (JAMA): 3**	Views (n.809)<br>Likes (n.19)<br>Dislikes (n.0)<br>Comments (n.27)<br>Subscriptions (n.37300000)<br><br>Like Ratio: 2.34	Information flow (3)<br>Information accuracy (3)<br>Quality (3)<br>Sensitivity (3)<br><br>**VIQI: 12**	Route of transmission (1)<br>HPV risk factors (1)<br>HPV screening (1)<br>Genotypes (0)<br>Oncogenic role (1)<br><br>Skin lesions (benign) (0)	Age (1)<br>Gender (0)<br>Type of vaccines (1)<br>HPV vaccine safety (1)<br><br>HPV vaccination advice:<br>Encouraging (1)<br>Discouraging (0)	Vaccination course (1)<br>Projections of genital cancer Fx (1)<br>Projections of genital cancer Mx (0)<br>Projection of OSCC (0)

		View Ratio: 361.16<br><br>**VPI: 8.45**		Mucosal lesions (benign) (0)<br>Oral lesions (benign) (0)<br><br>Genital cancer Fx (1)<br>Genital cancer Mx (1)<br>OSCC (0)<br><br>**Content on HPV infection and related lesions Score: 6**	Neutral (0)<br><br>Fake news (0)<br><br>**Content on HPV vaccination Score: 4**	**Content on Epidemiology Score: 2**<br><br>**VIDEO TOTAL-CONTENT SCORE: 12**<br><br>**GQS: 3**<br>**Video Educational Value:** medium/good/excellent
Link: https://www.youtube.com/watch?app=desktop&v=vFHjK5L0t-Y<br><br>Lenght (min): 12.27<br><br>Time elapsed since upload (days): 3080<br><br>Target audience: Laypersons	Source: Other<br><br>**Video Source Reliability (JAMA): 2**	Views (n.8191)<br>Likes (n.12)<br>Dislikes (n.9)<br>Comments (disabled)<br>Subscriptions (26500)<br><br>Like Ratio: 0.25<br>View Ratio: 265.94<br><br>**VPI: 0.66**	Information flow (2)<br>Information accuracy (2)<br>Quality (2)<br>Sensitivity (2)<br><br>**VIQI: 8**	Route of transmission (1)<br>HPV risk factors (1)<br>HPV screening (0)<br>Genotypes (0)<br>Oncogenic role (1)<br><br>Skin lesions (benign) (1)<br>Mucosal lesions (benign) (0)<br>Oral lesions (benign) (0)<br><br>Genital cancer Fx (1)<br>Genital cancer Mx (1)<br>OSCC (0)<br><br>**Content on HPV infection and related lesions Score: 6**	Age (1)<br>Gender (0)<br>Type of vaccines (0)<br>HPV vaccine safety (1)<br><br>HPV vaccination advice:<br>Encouraging (1)<br>Discouraging (0)<br>Neutral (0)<br><br>Fake news (0)<br><br>**Content on HPV vaccination Score: 3**	Vaccination course (1)<br>Projections of genital cancer Fx (0)<br>Projections of genital cancer Mx (0)<br>Projection of OSCC (0)<br><br>**Content on Epidemiology Score: 1**<br><br>**VIDEO TOTAL-CONTENT SCORE: 10**<br><br>**GQS: 2**<br>**Video Educational Value:** very low/low
Link: https://www.youtube.com/watch?v=E36aShFlEYo<br><br>Lenght (min): 5.34<br><br>Time elapsed since upload (days): 3334<br><br>Target audience: Laypersons	Source: Other<br><br>**Video Source Reliability (JAMA): 2**	Views (n.653309)<br>Likes (n.66)<br>Dislikes (n.22)<br>Comments (disabled)<br>Subscriptions (609000)<br><br>Like Ratio: 0.01<br>View Ratio: 19595.35<br><br>**VPI: 1.96**	Information flow (1)<br>Information accuracy (1)<br>Quality (4)<br>Sensitivity (2)<br><br>**VIQI: 8**	Route of transmission (0)<br>HPV risk factors (0)<br>HPV screening (0)<br>Genotypes (0)<br>Oncogenic role (1)<br><br>Skin lesions (benign) (0)<br>Mucosal lesions (benign) (0)<br>Oral lesions (benign) (0)<br><br>Genital cancer Fx (1)<br>Genital cancer Mx (0)<br>OSCC (0)	Age (1)<br>Gender (0)<br>Type of vaccines (0)<br>HPV vaccine safety (1)<br><br>HPV vaccination advice:<br>Encouraging (1)<br>Discouraging (0)<br>Neutral (0)<br><br>Fake news (0)<br><br>**Content on HPV vaccination Score: 3**	Vaccination course (0)<br>Projections of genital cancer Fx (0)<br>Projections of genital cancer Mx (0)<br>Projection of OSCC (0)<br><br>**Content on Epidemiology Score: 0**<br><br>**VIDEO TOTAL-CONTENT SCORE: 5**<br><br>**GQS: 1**

				**Content on HPV infection and related lesions Score: 2**		**Video Educational Value:** very low/low
Link: https://www.youtube.com/watch?v=lEXb3lOWGos<br><br>Lenght (min): 4.03<br><br>Time elapsed since upload (days): 300<br><br>Target audience: Both	Source: Hospital/university<br><br>**Video Source Reliability (JAMA): 3**	Views (n.203)<br>Likes (n.5)<br>Dislikes (n.0)<br>Comments (n.24)<br>Subscriptions (n.680)<br><br>Like Ratio: 2.46<br>View Ratio: 67.67<br><br>**VPI: 1.66**	Information flow (5)<br>Information accuracy (3)<br>Quality (4)<br>Sensitivity (3)<br><br>**VIQI: 15**	Route of transmission (1)<br>HPV risk factors (1)<br>HPV screening (1)<br>Genotypes (0)<br>Oncogenic role (1)<br><br>Skin lesions (benign) (1)<br>Mucosal lesions (benign) (0)<br>Oral lesions (benign) (0)<br><br>Genital cancer Fx (1)<br>Genital cancer Mx (1)<br>OSCC (0)<br><br>**Content on HPV infection and related lesions Score: 7**	Age (1)<br>Gender (1)<br>Type of vaccines (0)<br>HPV vaccine safety (0)<br><br>HPV vaccination advice:<br>Encouraging (0)<br>Discouraging (0)<br>Neutral (1)<br><br>Fake news (0)<br><br>**Content on HPV vaccination Score: 3**	Vaccination course (0)<br>Projections of genital cancer Fx (1)<br>Projections of genital cancer Mx (1)<br>Projection of OSCC (0)<br><br>**Content on Epidemiology Score: 2**<br><br>**VIDEO TOTAL-CONTENT SCORE: 12**<br><br>**GQS: 4**<br>**Video Educational Value:** medium/good/excellent
Link: https://www.youtube.com/watch?app=desktop&v=nJqderdey_I<br><br>Lenght (min): 6.51<br><br>Time elapsed since upload (days): 3340<br><br>Target audience: Laypersons	Source: Other healthcare provider (Pediatrician)<br><br>**Video Source Reliability (JAMA): 3**	Views (n.106615)<br>Likes (n.3064)<br>Dislikes (n.146)<br>Comments (n.608)<br>Subscriptions (n.441000)<br><br>Like Ratio: 3.01<br>View Ratio: 3192.06<br><br>**VPI: 96.08**	Information flow (3)<br>Information accuracy (3)<br>Quality (2)<br>Sensitivity (3)<br><br>**VIQI: 11**	Route of transmission (1)<br>HPV risk factors (1)<br>HPV screening (1)<br>Genotypes (0)<br>Oncogenic role (1)<br><br>Skin lesions (benign) (0)<br>Mucosal lesions (benign) (0)<br>Oral lesions (benign) (0)<br><br>Genital cancer Fx (1)<br>Genital cancer Mx (1)<br>OSCC (1)<br><br>**Content on HPV infection and related lesions Score: 7**	Age (1)<br>Gender (0)<br>Type of vaccines (0)<br>HPV vaccine safety (1)<br><br>HPV vaccination advice:<br>Encouraging (1)<br>Discouraging (0)<br>Neutral (0)<br><br>Fake news (0)<br><br>**Content on HPV vaccination Score: 3**	Vaccination course (1)<br>Projections of genital cancer Fx (1)<br>Projections of genital cancer Mx (0)<br>Projection of OSCC (0)<br><br>**Content on Epidemiology Score: 2**<br><br>**VIDEO TOTAL-CONTENT SCORE: 12**<br><br>**GQS: 3**<br>**Video Educational Value:** medium/good/excellent
Link: https://www.youtube.com/watch?v=t8CkP11PUJ0<br><br>Lenght (min): 5.41<br><br>Time elapsed since upload (days): 1603	Source: Other<br><br>**Video Source Reliability (JAMA): 1**	Views (n.14)<br>Likes (n.0)<br>Dislikes (n.0)<br>Comments (n.0)	Information flow (4)<br>Information accuracy (2)<br>Quality (2)	Route of transmission (0)<br>HPV risk factors (1)<br>HPV screening (0)<br>Genotypes (0)<br>Oncogenic role (1)	Age (1)<br>Gender (0)<br>Type of vaccines (0)<br>HPV vaccine safety (1)	Vaccination course (0)<br>Projections of genital cancer Fx (1)<br>Projections of genital cancer Mx (0)

Target audience: Laypersons		Subscriptions (n.321000)<br><br>Like Ratio: 0<br>View Ratio: 0.87<br><br>**VPI: 0**	Sensitivity (2)<br><br>**VIQI: 10**	Skin lesions (benign) (0)<br>Mucosal lesions (benign) (0)<br>Oral lesions (benign) (0)<br><br>Genital cancer Fx (1)<br>Genital cancer Mx (1)<br>OSCC (1)<br><br>**Content on HPV infection and related lesions Score: 5**	HPV vaccination advice:<br>Encouraging (1)<br>Discouraging (0)<br>Neutral (0)<br><br>Fake news (0)<br><br>**Content on HPV vaccination Score: 3**	Projection of OSCC (0)<br><br>**Content on Epidemiology Score: 1**<br><br>**VIDEO TOTAL-CONTENT SCORE: 9**<br><br>**GQS: 2**<br>**Video Educational Value:** very low/low
Link: https://www.youtube.com/watch?v=thF2ADpyxL4<br><br>Lenght (min): 4.30<br><br>Time elapsed since upload (days): 1078<br><br>Target audience: Both	Source: Other<br><br>**Video Source Reliability (JAMA): 2**	Views (n.162)<br>Likes (n.4)<br>Dislikes (n.0)<br>Comments (n.0)<br>Subscriptions (n.3300)<br><br>Like Ratio: 2.47<br>View Ratio: 15.03<br><br>**VPI: 0.37**	Information flow (5)<br>Information accuracy (4)<br>Quality (4)<br>Sensitivity (5)<br><br>**VIQI: 18**	Route of transmission (1)<br>HPV risk factors (1)<br>HPV screening (1)<br>Genotypes (1)<br>Oncogenic role (1)<br><br>Skin lesions (benign) (0)<br>Mucosal lesions (benign) (0)<br>Oral lesions (benign) (0)<br><br>Genital cancer Fx (0)<br>Genital cancer Mx (0)<br>OSCC (0)<br><br>**Content on HPV infection and related lesions Score: 5**	Age (1)<br>Gender (1)<br>Type of vaccines (1)<br>HPV vaccine safety (1)<br><br>HPV vaccination advice:<br>Encouraging (0)<br>Discouraging (0)<br>Neutral (1)<br><br>Fake news (0)<br><br>**Content on HPV vaccination Score: 5**	Vaccination course (1)<br>Projections of genital cancer Fx (0)<br>Projections of genital cancer Mx (0)<br>Projection of OSCC (0)<br><br>**Content on Epidemiology Score: 1**<br><br>**VIDEO TOTAL-CONTENT SCORE: 11**<br><br>**GQS: 5**<br>**Video Educational Value:** medium/good/excellent
Link: https://www.youtube.com/watch?app=desktop&v=GhpwK9wdrCM<br><br>Lenght (min): 8.52<br><br>Time elapsed since upload (days): 1235<br><br>Target audience: Laypersons	Source: Other<br><br>**Video Source Reliability (JAMA): 3**	Views (n.77)<br>Likes (n.1)<br>Dislikes (n.2)<br>Comments (n.2)<br>Subscriptions (3540)<br><br>Like Ratio: 3.89<br>View Ratio: 6.23<br><br>**VPI: 0.24**	Information flow (2)<br>Information accuracy (2)<br>Quality (1)<br>Sensitivity (3)<br><br>**VIQI: 8**	Route of transmission (1)<br>HPV risk factors (1)<br>HPV screening (1)<br>Genotypes (0)<br>Oncogenic role (1)<br><br>Skin lesions (benign) (1)<br>Mucosal lesions (benign) (0)<br>Oral lesions (benign) (0)<br><br>Genital cancer Fx (1)<br>Genital cancer Mx (0)	Age (1)<br>Gender (0)<br>Type of vaccines (1)<br>HPV vaccine safety (1)<br><br>HPV vaccination advice:<br>Encouraging (1)<br>Discouraging (0)<br>Neutral (0)<br><br>Fake news (0)	Vaccination course (1)<br>Projections of genital cancer Fx (0)<br>Projections of genital cancer Mx (0)<br>Projection of OSCC (0)<br><br>**Content on Epidemiology Score: 1**<br><br>**VIDEO TOTAL-CONTENT SCORE: 11**

				OSCC (0)<br><br>**Content on HPV infection and related lesions Score: 6**	**Content on HPV vaccination Score: 4**	**GQS: 2**<br>**Video Educational Value:** very low/low
Link:<br>https://www.youtube.com/watch?app=desktop&v=aAb5Ib9rMWs<br><br>Lenght (min): 5.14<br><br>Time elapsed since upload (days): 3399<br><br>Target audience: Laypersons	Source: Other<br><br>**Video Source Reliability (JAMA): 3**	Views (n.6017)<br>Likes (n.27)<br>Dislikes (n.0)<br>Comments (n.25)<br>Subscriptions (n.22300)<br><br>Like Ratio: 0.44<br>View Ratio: 177.02<br><br>**VPI: 0.77**	Information flow (2)<br>Information accuracy (2)<br>Quality (3)<br>Sensitivity (4)<br><br>**VIQI: 11**	Route of transmission (1)<br>HPV risk factors (1)<br>HPV screening (1)<br>Genotypes (0)<br>Oncogenic role (1)<br><br>Skin lesions (benign) (0)<br>Mucosal lesions (benign) (0)<br>Oral lesions (benign) (0)<br><br>Genital cancer Fx (1)<br>Genital cancer Mx (0)<br>OSCC (0)<br><br>**Content on HPV infection and related lesions Score: 5**	Age (1)<br>Gender (0)<br>Type of vaccines (0)<br>HPV vaccine safety (1)<br><br>HPV vaccination advice:<br>Encouraging (1)<br>Discouraging (0)<br>Neutral (0)<br><br>Fake news (0)<br><br>**Content on HPV vaccination Score: 3**	Vaccination course (0)<br>Projections of genital cancer Fx (0)<br>Projections of genital cancer Mx (1)<br>Projection of OSCC (0)<br><br>**Content on Epidemiology Score: 1**<br><br>**VIDEO TOTAL-CONTENT SCORE: 9**<br><br>**GQS: 2**<br>**Video Educational Value:** very low/low
Link:<br>https://www.youtube.com/watch?app=desktop&v=_LDW8mY49_Q<br><br>Lenght (min): 5.36<br><br>Time elapsed since upload (days): 2595<br><br>Target audience: Both	Source: Other<br><br>**Video Source Reliability (JAMA): 2**	Views (n.644)<br>Likes (disabled)<br>Dislikes (disabled)<br>Comments (disabled)<br>Subscriptions (n.26700)<br><br>Like Ratio: Not evaluable<br>View Ratio: 24.81<br><br>**VPI: Not evaluable**	Information flow (1)<br>Information accuracy (2)<br>Quality (1)<br>Sensitivity (2)<br><br>**VIQI: 6**	Route of transmission (1)<br>HPV risk factors (1)<br>HPV screening (1)<br>Genotypes (0)<br>Oncogenic role (1)<br><br>Skin lesions (benign) (0)<br>Mucosal lesions (benign) (0)<br>Oral lesions (benign) (0)<br><br>Genital cancer Fx (1)<br>Genital cancer Mx (0)<br>OSCC (0)<br><br>**Content on HPV infection and related lesions Score: 5**	Age (1)<br>Gender (0)<br>Type of vaccines (0)<br>HPV vaccine safety (1)<br><br>HPV vaccination advice:<br>Encouraging (1)<br>Discouraging (0)<br>Neutral (0)<br><br>Fake news (0)<br><br>**Content on HPV vaccination Score: 3**	Vaccination course (1)<br>Projections of genital cancer Fx (0)<br>Projections of genital cancer Mx (0)<br>Projection of OSCC (0)<br><br>**Content on Epidemiology Score: 1**<br><br>**VIDEO TOTAL-CONTENT SCORE: 9**<br><br>**GQS: 2**<br>**Video Educational Value:** very low/low
Link:<br>https://www.youtube.com/watch?app=desktop&v=giGMhFl3bYA	Source: Hospital/university	Views (n.2400)<br>Likes (disabled)<br>Dislikes (disabled)	Information flow (3)	Route of transmission (1)<br>HPV risk factors (1)<br>HPV screening (0)	Age (1)<br>Gender (0)<br>Type of vaccines (0)	Vaccination course (0)<br>Projections of genital cancer Fx (1)

Lenght (min): 4.59<br><br>Time elapsed since upload (days): 1952<br><br>Target audience: Laypersons	**Video Source Reliability (JAMA): 3**	Comments (0)<br>Subscriptions (190000)<br><br>Like Ratio: Not evaluable<br>View Ratio: 122.95<br><br>**VPI: Not evaluable**	Information accuracy (2)<br>Quality (1)<br>Sensitivity (2)<br><br>**VIQI: 8**	Genotypes (0)<br>Oncogenic role (1)<br><br>Skin lesions (benign) (0)<br>Mucosal lesions (benign) (0)<br>Oral lesions (benign) (0)<br><br>Genital cancer Fx (1)<br>Genital cancer Mx (1)<br>OSCC (0)<br><br>**Content on HPV infection and related lesions Score: 5**	HPV vaccine safety (1)<br><br>HPV vaccination advice:<br>Encouraging (1)<br>Discouraging (0)<br>Neutral (0)<br><br>Fake news (0)<br><br>**Content on HPV vaccination Score: 3**	Projections of genital cancer Mx (1)<br>Projection of OSCC (0)<br><br>**Content on Epidemiology Score: 2**<br><br>**VIDEO TOTAL-CONTENT SCORE: 10**<br><br>**GQS: 2**<br>**Video Educational Value:** very low/low
Link: https://www.youtube.com/watch?v=7axOCz1yCdQ<br><br>Lenght (min): 17.36<br><br>Time elapsed since upload (days): 3595<br><br>Target audience: Laypersons	Source: Other healthcare provider (Sexual health pysician)<br><br>**Video Source Reliability (JAMA): 3**	Views (n.1185)<br>Likes (n.2)<br>Dislikes (n.0)<br>Comments (disabled)<br>Subscriptions (2010)<br><br>Like Ratio: 0.16<br>View Ratio: 32.96<br><br>**VPI: 0.05**	Information flow (3)<br>Information accuracy (3)<br>Quality (1)<br>Sensitivity (4)<br><br>**VIQI: 11**	Route of transmission (1)<br>HPV risk factors (1)<br>HPV screening (1)<br>Genotypes (1)<br>Oncogenic role (1)<br><br>Skin lesions (benign) (1)<br>Mucosal lesions (benign) (0)<br>Oral lesions (benign) (0)<br><br>Genital cancer Fx (1)<br>Genital cancer Mx (1)<br>OSCC (0)<br><br>**Content on HPV infection and related lesions Score: 8**	Age (1)<br>Gender (0)<br>Type of vaccines (0)<br>HPV vaccine safety (1)<br><br>HPV vaccination advice:<br>Encouraging (1)<br>Discouraging (0)<br>Neutral (0)<br><br>Fake news (0)<br><br>**Content on HPV vaccination Score: 3**	Vaccination course (0)<br>Projections of genital cancer Fx (0)<br>Projections of genital cancer Mx (0)<br>Projection of OSCC (0)<br><br>**Content on Epidemiology Score: 0**<br><br>**VIDEO TOTAL-CONTENT SCORE: 11**<br><br>**GQS: 3**<br>**Video Educational Value:** medium/good/excellent
Link: https://www.youtube.com/watch?v=TFrFV99Tcy0<br><br>Lenght (min): 8.18<br><br>Time elapsed since upload (days): 1596<br><br>Target audience: Both	Source: Hospital/university<br><br>**Video Source Reliability (JAMA): 3**	Views (n.405)<br>Likes (n.3)<br>Dislikes (n.0)<br>Comments (n.0)<br>Subscriptions (n.1070)<br><br>Like Ratio: 0.74<br>View Ratio: 25.38<br><br>**VPI: 0.19**	Information flow (4)<br>Information accuracy (4)<br>Quality (2)<br>Sensitivity (4)<br><br>**VIQI: 14**	Route of transmission (0)<br>HPV risk factors (0)<br>HPV screening (0)<br>Genotypes (0)<br>Oncogenic role (1)<br><br>Skin lesions (benign) (0)<br>Mucosal lesions (benign) (1)<br>Oral lesions (benign) (0)	Age (1)<br>Gender (1)<br>Type of vaccines (1)<br>HPV vaccine safety (1)<br><br>HPV vaccination advice:<br>Encouraging (1)<br>Discouraging (0)<br>Neutral (0)<br><br>Fake news (0)	Vaccination course (1)<br>Projections of genital cancer Fx (0)<br>Projections of genital cancer Mx (0)<br>Projection of OSCC (0)<br><br>**Content on Epidemiology Score: 1**

				Genital cancer Fx (1)<br>Genital cancer Mx (1)<br>OSCC (1)<br><br>**Content on HPV infection and related lesions Score: 5**	**Content on HPV vaccination Score: 5**	**VIDEO TOTAL-CONTENT SCORE: 11**<br><br>**GQS: 4**<br>**Video Educational Value:** medium/good/excellent
Link: https://www.youtube.com/watch?v=KtuVjEKYdDI<br><br>Lenght (min): 7.32<br><br>Time elapsed since upload (days): 1247<br><br>Target audience: Both	Source: Other<br><br>**Video Source Reliability (JAMA): 1**	Views (n.115)<br>Likes (n.3)<br>Dislikes (n.2)<br>Comments (disabled)<br>Subscriptions (11300)<br><br>Like Ratio: 4.35<br>View Ratio: 9.22<br><br>**VPI: 0.40**	Information flow (1)<br>Information accuracy (1)<br>Quality (3)<br>Sensitivity (1)<br><br>**VIQI: 6**	Route of transmission (0)<br>HPV risk factors (0)<br>HPV screening (0)<br>Genotypes (0)<br>Oncogenic role (1)<br><br>Skin lesions (benign) (0)<br>Mucosal lesions (benign) (0)<br>Oral lesions (benign) (0)<br><br>Genital cancer Fx (1)<br>Genital cancer Mx (0)<br>OSCC (0)<br><br>**Content on HPV infection and related lesions Score: 2**	Age (0)<br>Gender (0)<br>Type of vaccines (0)<br>HPV vaccine safety (1)<br><br>HPV vaccination advice:<br>Encouraging (1)<br>Discouraging (0)<br>Neutral (0)<br><br>Fake news (0)<br><br>**Content on HPV vaccination Score: 2**	Vaccination course (1)<br>Projections of genital cancer Fx (0)<br>Projections of genital cancer Mx (0)<br>Projection of OSCC (0)<br><br>**Content on Epidemiology Score: 1**<br><br>**VIDEO TOTAL-CONTENT SCORE: 5**<br><br>**GQS: 1**<br>**Video Educational Value:** very low/low
Link: https://www.youtube.com/watch?v=cdDtQrjNyZg<br><br>Lenght (min): 6.49<br><br>Time elapsed since upload (days): 3483<br><br>Target audience: Both	Source: Other<br><br>**Video Source Reliability (JAMA): 3**	Views (n.792)<br>Likes (n.4)<br>Dislikes (n.0)<br>Comments (disabled)<br>Subscriptions (3470000)<br><br>Like Ratio: 0.50<br>View Ratio: 22.74<br><br>**VPI: 0.11**	Information flow (3)<br>Information accuracy (4)<br>Quality (2)<br>Sensitivity (4)<br><br>**VIQI: 13**	Route of transmission (1)<br>HPV risk factors (0)<br>HPV screening (0)<br>Genotypes (0)<br>Oncogenic role (1)<br><br>Skin lesions (benign) (0)<br>Mucosal lesions (benign) (0)<br>Oral lesions (benign) (0)<br><br>Genital cancer Fx (1)<br>Genital cancer Mx (1)<br>OSCC (1)<br><br>**Content on HPV infection and related lesions Score: 5**	Age (1)<br>Gender (1)<br>Type of vaccines (0)<br>HPV vaccine safety (0)<br><br>HPV vaccination advice:<br>Encouraging (1)<br>Discouraging (0)<br>Neutral (0)<br><br>Fake news (0)<br><br>**Content on HPV vaccination Score: 3**	Vaccination course (1)<br>Projections of genital cancer Fx (1)<br>Projections of genital cancer Mx (0)<br>Projection of OSCC (0)<br><br>**Content on Epidemiology Score: 2**<br><br>**VIDEO TOTAL-CONTENT SCORE: 10**<br><br>**GQS: 3**<br>**Video Educational Value:** medium/good/excellent

Link: https://www.youtube.com/watch?v=7lkbGPGr2EQ<br><br>Lenght (min): 8.18<br><br>Time elapsed since upload (days): 491<br><br>Target audience: Both	Source: Other<br><br>**Video Source Reliability (JAMA): 3**	Views (n.63)<br>Likes (disabled)<br>Dislikes (disabled)<br>Comments (disabled)<br>Subscriptions (n.15300)<br><br>Like Ratio: Not evaluable<br>View Ratio: 12.83<br><br>**VPI: Not evaluable**	Information flow (3)<br>Information accuracy (3)<br>Quality (3)<br>Sensitivity (4)<br><br>**VIQI: 13**	Route of transmission (0)<br>HPV risk factors (0)<br>HPV screening (1)<br>Genotypes (0)<br>Oncogenic role (1)<br><br>Skin lesions (benign) (0)<br>Mucosal lesions (benign) (0)<br>Oral lesions (benign) (0)<br><br>Genital cancer Fx (1)<br>Genital cancer Mx (1)<br>OSCC (1)<br><br>**Content on HPV infection and related lesions Score: 5**	Age (1)<br>Gender (1)<br>Type of vaccines (0)<br>HPV vaccine safety (0)<br><br>HPV vaccination advice:<br>Encouraging (1)<br>Discouraging (0)<br>Neutral (0)<br><br>Fake news (0)<br><br>**Content on HPV vaccination Score: 3**	Vaccination course (0)<br>Projections of genital cancer Fx (1)<br>Projections of genital cancer Mx (0)<br>Projection of OSCC (0)<br><br>**Content on Epidemiology Score: 1**<br><br>**VIDEO TOTAL-CONTENT SCORE: 9**<br><br>**GQS: 3**<br>**Video Educational Value:**<br>medium/good/excellent
Link: https://www.youtube.com/watch?v=2xnoTXzP0qs<br><br>Lenght (min): 4.34<br><br>Time elapsed since upload (days): 3322<br><br>Target audience: Laypersons	Source: Other<br><br>**Video Source Reliability (JAMA): 4**	Views (n.30119)<br>Likes (n.190)<br>Dislikes (n.44)<br>Comments (n.211)<br>Subscriptions (n.22700)<br><br>Like Ratio: 0.78<br>View Ratio: 906.65<br><br>**VPI: 7.07**	Information flow (4)<br>Information accuracy (4)<br>Quality (5)<br>Sensitivity (5)<br><br>**VIQI: 18**	Route of transmission (0)<br>HPV risk factors (0)<br>HPV screening (0)<br>Genotypes (0)<br>Oncogenic role (1)<br><br>Skin lesions (benign) (0)<br>Mucosal lesions (benign) (0)<br>Oral lesions (benign) (0)<br><br>Genital cancer Fx (1)<br>Genital cancer Mx (1)<br>OSCC (1)<br><br>**Content on HPV infection and related lesions Score: 4**	Age (1)<br>Gender (1)<br>Type of vaccines (1)<br>HPV vaccine safety (1)<br><br>HPV vaccination advice:<br>Encouraging (0)<br>Discouraging (0)<br>Neutral (1)<br><br>Fake news (0)<br><br>**Content on HPV vaccination Score: 5**	Vaccination course (1)<br>Projections of genital cancer Fx (0)<br>Projections of genital cancer Mx (0)<br>Projection of OSCC (0)<br><br>**Content on Epidemiology Score: 1**<br><br>**VIDEO TOTAL-CONTENT SCORE: 10**<br><br>**GQS: 4**<br>**Video Educational Value:**<br>medium/good/excellent
Link: https://www.youtube.com/watch?v=Ls0lKw3IDLY<br><br>Lenght (min): 5.11<br><br>Time elapsed since upload (days): 882<br><br>Target audience: Laypersons	Source: Other<br><br>**Video Source Reliability (JAMA): 4**	Views (n.124)<br>Likes (n.0)<br>Dislikes (n.0)<br>Comments (n.2)<br>Subscriptions (n.321000)	Information flow (4)<br>Information accuracy (3)<br>Quality (3)<br>Sensitivity (4)	Route of transmission (1)<br>HPV risk factors (1)<br>HPV screening (1)<br>Genotypes (1)<br>Oncogenic role (1)<br><br>Skin lesions (benign) (1)	Age (1)<br>Gender (0)<br>Type of vaccines (0)<br>HPV vaccine safety (0)<br><br>HPV vaccination advice:<br>Encouraging (0)	Vaccination course (0)<br>Projections of genital cancer Fx (0)<br>Projections of genital cancer Mx (0)<br>Projection of OSCC (0)

		Like Ratio: 0<br>View Ratio: 14.06<br><br>**VPI: 0**	**VIQI: 14**	Mucosal lesions (benign) (0)<br>Oral lesions (benign) (0)<br><br>Genital cancer Fx (1)<br>Genital cancer Mx (1)<br>OSCC (1)<br><br>**Content on HPV infection and related lesions Score: 9**	Discouraging (0)<br>Neutral (1)<br><br>Fake news (0)<br><br>**Content on HPV vaccination Score: 2**	**Content on Epidemiology Score: 0**<br><br>**VIDEO TOTAL-CONTENT SCORE: 11**<br><br>**GQS: 3**<br>**Video Educational Value:** medium/good/excellent
Link: https://www.youtube.com/watch?v=aC2x0yrkFx4<br><br>Lenght (min): 6.37<br><br>Time elapsed since upload (days): 559<br><br>Target audience: Professional	Source: Other<br><br>**Video Source Reliability (JAMA): 4**	Views (n.111)<br>Likes (n.0)<br>Dislikes (n.0)<br>Comments (disabled)<br>Subscriptions (n.411)<br><br>Like Ratio: 0<br>View Ratio: 19.85<br><br>**VPI: 0**	Information flow (2)<br>Information accuracy (2)<br>Quality (3)<br>Sensitivity (2)<br><br>**VIQI: 9**	Route of transmission (0)<br>HPV risk factors (0)<br>HPV screening (0)<br>Genotypes (0)<br>Oncogenic role (0)<br><br>Skin lesions (benign) (0)<br>Mucosal lesions (benign) (0)<br>Oral lesions (benign) (0)<br><br>Genital cancer Fx (0)<br>Genital cancer Mx (0)<br>OSCC (0)<br><br>**Content on HPV infection and related lesions Score: 0**	Age (1)<br>Gender (0)<br>Type of vaccines (0)<br>HPV vaccine safety (1)<br><br>HPV vaccination advice:<br>Encouraging (1)<br>Discouraging (0)<br>Neutral (0)<br><br>Fake news (0)<br><br>**Content on HPV vaccination Score: 3**	Vaccination course (1)<br>Projections of genital cancer Fx (0)<br>Projections of genital cancer Mx (0)<br>Projection of OSCC (0)<br><br>**Content on Epidemiology Score: 1**<br><br>**VIDEO TOTAL-CONTENT SCORE: 4**<br><br>**GQS: 1**<br>**Video Educational Value:** very low/low
Link: https://www.youtube.com/watch?app=desktop&v=yQtmtv5ZY7A<br><br>Lenght (min): 4.10<br><br>Time elapsed since upload (days): 332<br><br>Target audience: Laypersons	Source: Other<br><br>**Video Source Reliability (JAMA): 2**	Views (n.1810)<br>Likes (n.22)<br>Dislikes (n.23)<br>Comments (n.44)<br>Subscriptions (n.1330000)<br><br>Like Ratio: 2.48<br>View Ratio: 545.18<br><br>**VPI: 13.52**	Information flow (2)<br>Information accuracy (2)<br>Quality (1)<br>Sensitivity (3)<br><br>**VIQI: 8**	Route of transmission (0)<br>HPV risk factors (0)<br>HPV screening (0)<br>Genotypes (0)<br>Oncogenic role (1)<br><br>Skin lesions (benign) (0)<br>Mucosal lesions (benign) (0)<br>Oral lesions (benign) (0)<br><br>Genital cancer Fx (1)<br>Genital cancer Mx (0)<br>OSCC (0)	Age (1)<br>Gender (0)<br>Type of vaccines (0)<br>HPV vaccine safety (1)<br><br>HPV vaccination advice:<br>Encouraging (1)<br>Discouraging (0)<br>Neutral (0)<br><br>Fake news (0)<br><br>**Content on HPV vaccination Score: 3**	Vaccination course (1)<br>Projections of genital cancer Fx (1)<br>Projections of genital cancer Mx (0)<br>Projection of OSCC (0)<br><br>**Content on Epidemiology Score: 2**<br>**VIDEO TOTAL-CONTENT SCORE: 7**<br><br>**GQS: 2**<br>**Video Educational Value:** very low/low

				**Content on HPV infection and related lesions Score: 2**		
Link: https://www.youtube.com/watch?app=desktop&v=So0zr__z6S4<br><br>Lenght (min): 10.36<br><br>Time elapsed since upload (days): 787<br><br>Target audience: Laypersons	Source: Other healthcare provider (Obstetrician-Gynecologist)<br><br>**Video Source Reliability (JAMA): 3**	Views (n.139737)<br>Likes (n.7461)<br>Dislikes (n.123)<br>Comments (n.1315)<br>Subscriptions (n.1240000)<br><br>Like Ratio: 5.42<br>View Ratio: 17755.65<br><br>**VPI: 962.35**	Information flow (2)<br>Information accuracy (3)<br>Quality (1)<br>Sensitivity (2)<br><br>**VIQI: 8**	Route of transmission (1)<br>HPV risk factors (0)<br>HPV screening (0)<br>Genotypes (1)<br>Oncogenic role (1)<br><br>Skin lesions (benign) (0)<br>Mucosal lesions (benign) (0)<br>Oral lesions (benign) (0)<br><br>Genital cancer Fx (1)<br>Genital cancer Mx (1)<br>OSCC (1)<br><br>**Content on HPV infection and related lesions Score: 6**	Age (1)<br>Gender (0)<br>Type of vaccines (1)<br>HPV vaccine safety (1)<br><br>HPV vaccination advice:<br>Encouraging (1)<br>Discouraging (0)<br>Neutral (0)<br><br>Fake news (0)<br><br>**Content on HPV vaccination Score: 4**	Vaccination course (1)<br>Projections of genital cancer Fx (0)<br>Projections of genital cancer Mx (0)<br>Projection of OSCC (0)<br><br>**Content on Epidemiology Score: 1**<br><br>**VIDEO TOTAL-CONTENT SCORE: 11**<br><br>**GQS: 2**<br>**Video Educational Value:** very low/low
Link: https://www.youtube.com/watch?app=desktop&v=3I2hWEpdBLE<br><br>Lenght (min): 4.38<br><br>Time elapsed since upload (days): 1449<br><br>Target audience: Laypersons	Source: Other<br><br>**Video Source Reliability (JAMA): 2**	Views (n.414)<br>Likes (n.6)<br>Dislikes (n.0)<br>Comments (n.22)<br>Subscriptions (n.131000)<br><br>Like Ratio: 1.44<br>View Ratio: 28.57<br><br>**VPI: 0.41**	Information flow (3)<br>Information accuracy (3)<br>Quality (2)<br>Sensitivity (3)<br><br>**VIQI: 11**	Route of transmission (1)<br>HPV risk factors (0)<br>HPV screening (0)<br>Genotypes (0)<br>Oncogenic role (1)<br><br>Skin lesions (benign) (0)<br>Mucosal lesions (benign) (0)<br>Oral lesions (benign) (0)<br><br>Genital cancer Fx (1)<br>Genital cancer Mx (1)<br>OSCC (0)<br><br>**Content on HPV infection and related lesions Score: 4**	Age (1)<br>Gender (0)<br>Type of vaccines (1)<br>HPV vaccine safety (1)<br><br>HPV vaccination advice:<br>Encouraging (1)<br>Discouraging (0)<br>Neutral (0)<br><br>Fake news (0)<br><br>**Content on HPV vaccination Score: 4**	Vaccination course (1)<br>Projections of genital cancer Fx (1)<br>Projections of genital cancer Mx (0)<br>Projection of OSCC (0)<br><br>**Content on Epidemiology Score: 2**<br><br>**VIDEO TOTAL-CONTENT SCORE: 10**<br><br>**GQS: 2**
Link: https://www.youtube.com/watch?v=eJrpzMeOUsY<br><br>Lenght (min): 7.18<br><br>Time elapsed since upload (days): 708	Source: Other<br><br>**Video Source Reliability (JAMA): 1**	Views (n.701)<br>Likes (n.11)<br>Dislikes (n.0)<br>Comments (n.2)	Information flow (3)<br>Information accuracy (2)<br>Quality (2)	Route of transmission (1)<br>HPV risk factors (0)<br>HPV screening (1)<br>Genotypes (1)<br>Oncogenic role (1)	Age (0)<br>Gender (0)<br>Type of vaccines (1)<br>HPV vaccine safety (1)	Vaccination course (0)<br>Projections of genital cancer Fx (1)<br>Projections of genital cancer Mx (0)

Target audience: Laypersons		Subscriptions (n.672000)<br><br>Like Ratio: 1.57<br>View Ratio: 99.01<br><br>**VPI: 1.55**	Sensitivity (3)<br><br>**VIQI: 10**	Skin lesions (benign) (0)<br>Mucosal lesions (benign) (0)<br>Oral lesions (benign) (0)<br><br>Genital cancer Fx (1)<br>Genital cancer Mx (0)<br>OSCC (0)<br><br>**Content on HPV infection and related lesions Score: 5**	HPV vaccination advice:<br>Encouraging (0)<br>Discouraging (0)<br>Neutral (1)<br><br>Fake news (0)<br><br>**Content on HPV vaccination Score: 3**	Projection of OSCC (0)<br><br>**Content on Epidemiology Score: 1**<br><br>**VIDEO TOTAL-CONTENT SCORE: 9**<br><br>**GQS: 3**
Link: https://www.youtube.com/watch?v=hl149tNGS_I<br><br>Lenght (min): 7.58<br><br>Time elapsed since upload (days): 1780<br><br>Target audience: Laypersons	Source: Other<br><br>**Video Source Reliability (JAMA): 2**	Views (n.189)<br>Likes (n.2)<br>Dislikes (n.0)<br>Comments (n.1)<br>Subscriptions (n.1470)<br><br>Like Ratio: 1.06<br>View Ratio: 10.62<br><br>**VPI: 11.26**	Information flow (1)<br>Information accuracy (1)<br>Quality (4)<br>Sensitivity (3)<br><br>**VIQI: 9**	Route of transmission (0)<br>HPV risk factors (0)<br>HPV screening (0)<br>Genotypes (0)<br>Oncogenic role (1)<br><br>Skin lesions (benign) (0)<br>Mucosal lesions (benign) (0)<br>Oral lesions (benign) (0)<br><br>Genital cancer Fx (1)<br>Genital cancer Mx (1)<br>OSCC (1)<br><br>**Content on HPV infection and related lesions Score: 4**	Age (1)<br>Gender (1)<br>Type of vaccines (0)<br>HPV vaccine safety (1)<br><br>HPV vaccination advice:<br>Encouraging (1)<br>Discouraging (0)<br>Neutral (0)<br><br>Fake news (0)<br><br>**Content on HPV vaccination Score: 4**	Vaccination course (0)<br>Projections of genital cancer Fx (0)<br>Projections of genital cancer Mx (0)<br>Projection of OSCC (0)<br><br>**Content on Epidemiology Score: 0**<br><br>**VIDEO TOTAL-CONTENT SCORE: 8**<br><br>**GQS: 1**
Link: https://www.youtube.com/watch?v=YXchqMdaA18<br><br>Lenght (min): 14.09<br><br>Time elapsed since upload (days): 470<br><br>Target audience: Both	Source: Other<br><br>**Video Source Reliability (JAMA): 3**	Views (n.2368)<br>Likes (n.45)<br>Dislikes (n.4)<br>Comments (n.20)<br>Subscriptions (n.3150000)<br><br>Like Ratio: 2.07<br>View Ratio: 503.83<br><br>**VPI: 10.43**	Information flow (3)<br>Information accuracy (3)<br>Quality (2)<br>Sensitivity (4)<br><br>**VIQI: 12**	Route of transmission (0)<br>HPV risk factors (0)<br>HPV screening (0)<br>Genotypes (0)<br>Oncogenic role (1)<br><br>Skin lesions (benign) (0)<br>Mucosal lesions (benign) (1)<br>Oral lesions (benign) (0)<br><br>Genital cancer Fx (1)<br>Genital cancer Mx (1)	Age (1)<br>Gender (1)<br>Type of vaccines (1)<br>HPV vaccine safety (1)<br><br>HPV vaccination advice:<br>Encouraging (0)<br>Discouraging (0)<br>Neutral (1)<br><br>Fake news (0)	Vaccination course (0)<br>Projections of genital cancer Fx (0)<br>Projections of genital cancer Mx (0)<br>Projection of OSCC (0)<br><br>**Content on Epidemiology Score: 0**<br>**VIDEO TOTAL-CONTENT SCORE: 9**<br><br>**GQS: 3**

				OSCC (0)<br><br>**Content on HPV infection and related lesions Score: 4**	**Content on HPV vaccination Score: 5**	
Link: https://www.youtube.com/watch?v=MDPqm__y2mY<br><br>Lenght (min): 12.38<br><br>Time elapsed since upload (days): 1602<br><br>Target audience: Laypersons	Source: Other<br><br>**Video Source Reliability (JAMA): 1**	Views (n.1130)<br>Likes (n.38)<br>Dislikes (n.0)<br>Comments (n.6)<br>Subscriptions (n.9670)<br><br>Like Ratio: 3.36<br>View Ratio: 70.54<br><br>**VPI: 2.37**	Information flow (2)<br>Information accuracy (1)<br>Quality (1)<br>Sensitivity (1)<br><br>**VIQI: 5**	Route of transmission (0)<br>HPV risk factors (0)<br>HPV screening (0)<br>Genotypes (0)<br>Oncogenic role (1)<br><br>Skin lesions (benign) (0)<br>Mucosal lesions (benign) (0)<br>Oral lesions (benign) (0)<br><br>Genital cancer Fx (1)<br>Genital cancer Mx (0)<br>OSCC (0)<br><br>**Content on HPV infection and related lesions Score: 2**	Age (1)<br>Gender (0)<br>Type of vaccines (1)<br>HPV vaccine safety (0)<br><br>HPV vaccination advice:<br>Encouraging (0)<br>Discouraging (1)<br>Neutral (0)<br><br>Fake news (0)<br><br>**Content on HPV vaccination Score: 3**	Vaccination course (0)<br>Projections of genital cancer Fx (0)<br>Projections of genital cancer Mx (0)<br>Projection of OSCC (0)<br><br>**Content on Epidemiology Score: 0**<br><br>**VIDEO TOTAL-CONTENT SCORE: 5**<br><br>**GQS: 1**
Link:<br>https://www.youtube.com/watch?app=desktop&v=01WpAlKiWFs<br><br>Lenght (min): 5.03<br><br>Time elapsed since upload (days): 1259<br><br>Target audience: Laypersons	Source: Other healthcare provider (Infectious disease specialist)<br><br>**Video Source Reliability (JAMA): 3**	Views (n.193)<br>Likes (disabled)<br>Dislikes (disabled)<br>Comments (disabled)<br>Subscriptions (772)<br><br>Like Ratio: Not evaluable<br>View Ratio: 15.33<br><br>**VPI: Not evaluable**	Information flow (2)<br>Information accuracy (2)<br>Quality (1)<br>Sensitivity (3)<br><br>**VIQI: 8**	Route of transmission (1)<br>HPV risk factors (0)<br>HPV screening (0)<br>Genotypes (0)<br>Oncogenic role (1)<br><br>Skin lesions (benign) (0)<br>Mucosal lesions (benign) (0)<br>Oral lesions (benign) (0)<br><br>Genital cancer Fx (1)<br>Genital cancer Mx (0)<br>OSCC (1)<br><br>**Content on HPV infection and related lesions Score: 4**	Age (0)<br>Gender (0)<br>Type of vaccines (0)<br>HPV vaccine safety (1)<br><br>HPV vaccination advice:<br>Encouraging (1)<br>Discouraging (0)<br>Neutral (0)<br><br>Fake news (0)<br><br>**Content on HPV vaccination Score: 2**	Vaccination course (0)<br>Projections of genital cancer Fx (0)<br>Projections of genital cancer Mx (1)<br>Projection of OSCC (1)<br><br>**Content on Epidemiology Score: 1**<br><br>**VIDEO TOTAL-CONTENT SCORE: 8**<br><br>**GQS: 2**
Link: https://www.youtube.com/watch?v=xk84dbXRU9M<br><br>Lenght (min): 6.33	Source: Hospital/university	Views (n.721)<br>Likes (n.5)<br>Dislikes (n.2)	Information flow (5)	Route of transmission (0)<br>HPV risk factors (0)<br>HPV screening (0)	Age (1)<br>Gender (1)<br>Type of vaccines (0)	Vaccination course (0)<br>Projections of genital cancer Fx (0)

Time elapsed since upload (days): 1634<br><br>Target audience: Both	**Video Source Reliability (JAMA): 3**	Comments (n.4)<br>Subscriptions (n.965)<br><br>Like Ratio: 0.97<br>View Ratio: 44.12<br><br>**VPI: 0.43**	Information accuracy (4)<br>Quality (4)<br>Sensitivity (5)<br><br>**VIQI: 18**	Genotypes (1)<br>Oncogenic role (1)<br><br>Skin lesions (benign) (1)<br>Mucosal lesions (benign) (1)<br>Oral lesions (benign) (1)<br><br>Genital cancer Fx (1)<br>Genital cancer Mx (1)<br>OSCC (1)<br><br>**Content on HPV infection and related lesions Score: 8**	HPV vaccine safety (1)<br><br>HPV vaccination advice:<br>Encouraging (1)<br>Discouraging (0)<br>Neutral (0)<br><br>Fake news (0)<br><br>**Content on HPV vaccination Score: 4**	Projections of genital cancer Mx (0)<br>Projection of OSCC (0)<br><br>**Content on Epidemiology Score: 0**<br><br>**VIDEO TOTAL-CONTENT SCORE: 12**<br><br>**GQS: 4**
Link: https://www.youtube.com/watch?v=5G426xW46fo<br><br>Lenght (min): 11.10<br><br>Time elapsed since upload (days): 3631<br><br>Target audience: Both	Source: Other<br><br>**Video Source Reliability (JAMA): 3**	Views (n.494)<br>Likes (n.2)<br>Dislikes (n.5)<br>Comments (n.4)<br>Subscriptions (n.1680)<br><br>Like Ratio: 1.42<br>View Ratio: 13.60<br><br>**VPI: 0.19**	Information flow (4)<br>Information accuracy (4)<br>Quality (2)<br>Sensitivity (2)<br><br>**VIQI: 12**	Route of transmission (1)<br>HPV risk factors (0)<br>HPV screening (1)<br>Genotypes (0)<br>Oncogenic role (1)<br><br>Skin lesions (benign) (0)<br>Mucosal lesions (benign) (1)<br>Oral lesions (benign) (0)<br><br>Genital cancer Fx (1)<br>Genital cancer Mx (1)<br>OSCC (1)<br><br>**Content on HPV infection and related lesions Score: 7**	Age (1)<br>Gender (1)<br>Type of vaccines (0)<br>HPV vaccine safety (0)<br><br>HPV vaccination advice:<br>Encouraging (1)<br>Discouraging (0)<br>Neutral (0)<br><br>Fake news (0)<br><br>**Content on HPV vaccination Score: 3**	Vaccination course (0)<br>Projections of genital cancer Fx (0)<br>Projections of genital cancer Mx (0)<br>Projection of OSCC (0)<br><br>**Content on Epidemiology Score: 0**<br><br>**VIDEO TOTAL-CONTENT SCORE: 10**<br><br>**GQS: 3**
Link: https://www.youtube.com/watch?v=z0MJI1J1Z-M<br><br>Lenght (min): 7.47<br><br>Time elapsed since upload (days): 1374<br><br>Target audience: Professional	Source: Other<br><br>**Video Source Reliability (JAMA): 4**	Views (n.587)<br>Likes (n.11)<br>Dislikes (n.0)<br>Comments (n.11)<br>Subscriptions (n.29800)<br><br>Like Ratio: 1.87<br>View Ratio: 42.72<br><br>**VPI: 0.80**	Information flow (4)<br>Information accuracy (5)<br>Quality (4)<br>Sensitivity (4)<br><br>**VIQI: 17**	Route of transmission (0)<br>HPV risk factors (0)<br>HPV screening (1)<br>Genotypes (1)<br>Oncogenic role (1)<br><br>Skin lesions (benign) (0)<br>Mucosal lesions (benign) (0)<br>Oral lesions (benign) (0)	Age (0)<br>Gender (0)<br>Type of vaccines (1)<br>HPV vaccine safety (0)<br><br>HPV vaccination advice:<br>Encouraging (1)<br>Discouraging (0)<br>Neutral (0)<br><br>Fake news (0)	Vaccination course (1)<br>Projections of genital cancer Fx (1)<br>Projections of genital cancer Mx (0)<br>Projection of OSCC (0)<br><br>**Content on Epidemiology Score: 2**<br>**VIDEO TOTAL-CONTENT SCORE: 10**

				Genital cancer Fx (1)<br>Genital cancer Mx (1)<br>OSCC (1)<br><br>**Content on HPV infection and related lesions Score: 6**	**Content on HPV vaccination Score: 2**	**GQS: 2**
Link: https://www.youtube.com/watch?v=1UDzAO0do6c<br><br>Lenght (min): 13.07<br><br>Time elapsed since upload (days): 1219<br><br>Target audience: Both	Source: Hospital/university<br><br>**Video Source Reliability (JAMA): 3**	Views (n.17)<br>Likes (n.0)<br>Dislikes (n.0)<br>Comments (disabled)<br>Subscriptions (n.4390)<br><br>Like Ratio: 0<br>View Ratio: 1.39<br><br>**VPI: 0**	Information flow (2)<br>Information accuracy (3)<br>Quality (2)<br>Sensitivity (3)<br><br>**VIQI: 10**	Route of transmission (1)<br>HPV risk factors (0)<br>HPV screening (0)<br>Genotypes (1)<br>Oncogenic role (1)<br><br>Skin lesions (benign) (0)<br>Mucosal lesions (benign) (0)<br>Oral lesions (benign) (0)<br><br>Genital cancer Fx (1)<br>Genital cancer Mx (0)<br>OSCC (1)<br><br>**Content on HPV infection and related lesions Score: 5**	Age (1)<br>Gender (1)<br>Type of vaccines (0)<br>HPV vaccine safety (1)<br><br>HPV vaccination advice:<br>Encouraging (1)<br>Discouraging (0)<br>Neutral (0)<br><br>Fake news (0)<br><br>**Content on HPV vaccination Score: 4**	Vaccination course (1)<br>Projections of genital cancer Fx (1)<br>Projections of genital cancer Mx (0)<br>Projection of OSCC (1)<br><br>**Content on Epidemiology Score: 3**<br><br>**VIDEO TOTAL-CONTENT SCORE: 12**<br><br>**GQS: 3**
Link: https://www.youtube.com/watch?app=desktop&v=6142nwh8JmI<br><br>Lenght (min): 17.08<br><br>Time elapsed since upload (days): 2610<br><br>Target audience: Both	Source: Hospital/university<br><br>**Video Source Reliability (JAMA): 4**	Views (n.45)<br>Likes (n.0)<br>Dislikes (n.0)<br>Comments (n.7)<br>Subscriptions (n.598)<br><br>Like Ratio: 0<br>View Ratio: 1.72<br><br>**VPI: 0**	Information flow (4)<br>Information accuracy (4)<br>Quality (3)<br>Sensitivity (2)<br><br>**VIQI: 13**	Route of transmission (0)<br>HPV risk factors (0)<br>HPV screening (1)<br>Genotypes (1)<br>Oncogenic role (1)<br><br>Skin lesions (benign) (1)<br>Mucosal lesions (benign) (1)<br>Oral lesions (benign) (0)<br><br>Genital cancer Fx (1)<br>Genital cancer Mx (0)<br>OSCC (1)<br><br>**Content on HPV infection and related lesions Score: 7**	Age (0)<br>Gender (0)<br>Type of vaccines (0)<br>HPV vaccine safety (1)<br><br>HPV vaccination advice:<br>Encouraging (1)<br>Discouraging (0)<br>Neutral (0)<br><br>Fake news (0)<br><br>**Content on HPV vaccination Score: 2**	Vaccination course (0)<br>Projections of genital cancer Fx (1)<br>Projections of genital cancer Mx (0)<br>Projection of OSCC (0)<br><br>**Content on Epidemiology Score: 1**<br><br>**VIDEO TOTAL-CONTENT SCORE: 10**<br><br>**GQS: 3**

Link: https://www.youtube.com/watch?app=desktop&v=HY1pUdSgLfQ<br><br>Lenght (min): 4.40<br><br>Time elapsed since upload (days): 3576<br><br>Target audience: Both	Source: Hospital/university<br><br>**Video Source Reliability (JAMA): 3**	Views (n.347)<br>Likes (n.4)<br>Dislikes (n.0)<br>Comments (n.13)<br>Subscriptions (n.1550)<br><br>Like Ratio: 1.15<br>View Ratio: 9.70<br><br>**VPI: 0.11**	Information flow (2)<br>Information accuracy (3)<br>Quality (2)<br>Sensitivity (2)<br><br>**VIQI: 9**	Route of transmission (1)<br>HPV risk factors (1)<br>HPV screening (1)<br>Genotypes (0)<br>Oncogenic role (1)<br><br>Skin lesions (benign) (1)<br>Mucosal lesions (benign) (1)<br>Oral lesions (benign) (0)<br><br>Genital cancer Fx (1)<br>Genital cancer Mx (0)<br>OSCC (0)<br><br>**Content on HPV infection and related lesions Score: 7**	Age (1)<br>Gender (0)<br>Type of vaccines (0)<br>HPV vaccine safety (1)<br><br>HPV vaccination advice:<br>Encouraging (1)<br>Discouraging (0)<br>Neutral (0)<br><br>Fake news (0)<br><br>**Content on HPV vaccination Score: 3**	Vaccination course (1)<br>Projections of genital cancer Fx (1)<br>Projections of genital cancer Mx (0)<br>Projection of OSCC (0)<br><br>**Content on Epidemiology Score: 2**<br><br>**VIDEO TOTAL-CONTENT SCORE: 12**<br><br>**GQS: 3**
Link: https://www.youtube.com/watch?app=desktop&v=b9QrKeDtZ9U<br><br>Lenght (min): 5.15<br><br>Time elapsed since upload (days): 4093<br><br>Target audience: Laypersons	Source: Other<br><br>**Video Source Reliability (JAMA): 2**	Views (n.3902)<br>Likes (n.14)<br>Dislikes (n.13)<br>Comments (disabled)<br>Subscriptions (n.3470000)<br><br>Like Ratio: 0.69<br>View Ratio: 95.33<br><br>**VPI: 0.65**	Information flow (3)<br>Information accuracy (3)<br>Quality (1)<br>Sensitivity (4)<br><br>**VIQI: 11**	Route of transmission (1)<br>HPV risk factors (1)<br>HPV screening (1)<br>Genotypes (0)<br>Oncogenic role (1)<br><br>Skin lesions (benign) (1)<br>Mucosal lesions (benign) (0)<br>Oral lesions (benign) (0)<br><br>Genital cancer Fx (1)<br>Genital cancer Mx (1)<br>OSCC (1)<br><br>**Content on HPV infection and related lesions Score: 8**	Age (1)<br>Gender (0)<br>Type of vaccines (0)<br>HPV vaccine safety (1)<br><br>HPV vaccination advice:<br>Encouraging (1)<br>Discouraging (0)<br>Neutral (0)<br><br>Fake news (0)<br><br>**Content on HPV vaccination Score: 3**	Vaccination course (1)<br>Projections of genital cancer Fx (0)<br>Projections of genital cancer Mx (1)<br>Projection of OSCC (0)<br><br>**Content on Epidemiology Score: 2**<br><br>**VIDEO TOTAL-CONTENT SCORE: 13**<br><br>**GQS: 2**
Link: https://www.youtube.com/watch?app=desktop&v=mStRHcwj7uU<br><br>Lenght (min): 13.12<br><br>Time elapsed since upload (days): 7<br><br>Target audience: Laypersons	Source: Hospital/university<br><br>**Video Source Reliability (JAMA): 3**	Views (n.77)<br>Likes (n.2)<br>Dislikes (n.0)<br>Comments (disabled)<br>Subscriptions (41400)	Information flow (3)<br>Information accuracy (4)<br>Quality (1)<br>Sensitivity (3)<br><br>**VIQI: 11**	Route of transmission (1)<br>HPV risk factors (1)<br>HPV screening (1)<br>Genotypes (0)<br>Oncogenic role (1)<br><br>Skin lesions (benign) (1)	Age (1)<br>Gender (0)<br>Type of vaccines (0)<br>HPV vaccine safety (1)<br><br>HPV vaccination advice:<br>Encouraging (1)<br>Discouraging (0)	Vaccination course (1)<br>Projections of genital cancer Fx (1)<br>Projections of genital cancer Mx (1)<br>Projection of OSCC (0)

		Like Ratio: 9.09<br>View Ratio: 1100<br><br>**VPI: 99.99**		Mucosal lesions (benign) (0)<br>Oral lesions (benign) (0)<br><br>Genital cancer Fx (1)<br>Genital cancer Mx (1)<br>OSCC (1)<br><br>**Content on HPV infection and related lesions Score: 8**	Neutral (0)<br><br>Fake news (0)<br><br>**Content on HPV vaccination Score: 3**	**Content on Epidemiology Score: 3**<br><br>**VIDEO TOTAL-CONTENT SCORE: 14**<br><br>**GQS: 3**
Link: https://www.youtube.com/watch?v=b-ask3b-_a8<br><br>Lenght (min): 17.10<br><br>Time elapsed since upload (days): 79<br><br>Target audience: Both	Source: Other<br><br>**Video Source Reliability (JAMA): 3**	Views (n.83)<br>Likes (n.2)<br>Dislikes (n.0)<br>Comments (n.3)<br>Subscriptions (n.1090)<br><br>Like Ratio: 2.40<br>View Ratio: 105.06<br><br>**VPI: 2.52**	Information flow (3)<br>Information accuracy (2)<br>Quality (1)<br>Sensitivity (2)<br><br>**VIQI: 8**	Route of transmission (1)<br>HPV risk factors (1)<br>HPV screening (1)<br>Genotypes (0)<br>Oncogenic role (1)<br><br>Skin lesions (benign) (0)<br>Mucosal lesions (benign) (0)<br>Oral lesions (benign) (0/)<br><br>Genital cancer Fx (1)<br>Genital cancer Mx (0)<br>OSCC (0)<br><br>**Content on HPV infection and related lesions Score: 5**	Age (1)<br>Gender (0)<br>Type of vaccines (0)<br>HPV vaccine safety (1)<br><br>HPV vaccination advice:<br>Encouraging (1)<br>Discouraging (0)<br>Neutral (0)<br><br>Fake news (0)<br><br>**Content on HPV vaccination Score: 3**	Vaccination course (1)<br>Projections of genital cancer Fx (1)<br>Projections of genital cancer Mx (0)<br>Projection of OSCC (0)<br><br>**Content on Epidemiology Score: 2**<br><br>**VIDEO TOTAL-CONTENT SCORE: 10**<br><br>**GQS: 2**
Link: https://www.youtube.com/watch?app=desktop&v=bc4d7XzKOXo<br><br>Lenght (min): 10.32<br><br>Time elapsed since upload (days): 1399<br><br>Target audience: Professional	Source: Other<br><br>**Video Source Reliability (JAMA): 3**	Views (n.28412)<br>Likes (n.165)<br>Dislikes (n.4)<br>Comments (disabled)<br>Subscriptions (n.2000)<br><br>Like Ratio: 0.59<br>View Ratio: 2030.87<br><br>**VPI: 11.98**	Information flow (4)<br>Information accuracy (3)<br>Quality (2)<br>Sensitivity (3)<br><br>**VIQI: 12**	Route of transmission (1)<br>HPV risk factors (1)<br>HPV screening (1)<br>Genotypes (1)<br>Oncogenic role (1)<br><br>Skin lesions (benign) (1)<br>Mucosal lesions (benign) (0)<br>Oral lesions (benign) (0)<br><br>Genital cancer Fx (1)<br>Genital cancer Mx (1)<br>OSCC (0)	Age (1)<br>Gender (0)<br>Type of vaccines (1)<br>HPV vaccine safety (1)<br><br>HPV vaccination advice:<br>Encouraging (1)<br>Discouraging (0)<br>Neutral (0)<br><br>Fake news (0)<br><br>**Content on HPV vaccination Score: 4**	Vaccination course (1)<br>Projections of genital cancer Fx (1)<br>Projections of genital cancer Mx (1)<br>Projection of OSCC (0)<br><br>**Content on Epidemiology Score: 3**<br><br>**VIDEO TOTAL-CONTENT SCORE: 15**<br><br>**GQS: 3**

				**Content on HPV infection and related lesions Score: 8**		
Link: https://www.youtube.com/watch?app=desktop&v=H1pC_lkHLg4<br><br>Lenght (min): 8.05<br><br>Time elapsed since upload (days): 93<br><br>Target audience: Laypersons	Source: Other<br><br>**Video Source Reliability (JAMA): 2**	Views (n.148)<br>Likes (n.2)<br>Dislikes (n.0)<br>Comments (n.0)<br>Subscriptions (n.10100)<br><br>Like Ratio: 1.35<br>View Ratio: 159.39<br><br>**VPI: 2.15**	Information flow (4)<br>Information accuracy (3)<br>Quality (3)<br>Sensitivity (5)<br><br>**VIQI: 15**	Route of transmission (1)<br>HPV risk factors (1)<br>HPV screening (0)<br>Genotypes (0)<br>Oncogenic role (1)<br><br>Skin lesions (benign) (0)<br>Mucosal lesions (benign) (0)<br>Oral lesions (benign) (0)<br><br>Genital cancer Fx (1)<br>Genital cancer Mx (0)<br>OSCC (0)<br><br>**Content on HPV infection and related lesions Score: 4**	Age (1)<br>Gender (0)<br>Type of vaccines (0)<br>HPV vaccine safety (1)<br><br>HPV vaccination advice:<br>Encouraging (1)<br>Discouraging (0)<br>Neutral (0)<br><br>Fake news (0)<br><br>**Content on HPV vaccination Score: 3**	Vaccination course (1)<br>Projections of genital cancer Fx (1)<br>Projections of genital cancer Mx (0)<br>Projection of OSCC (0)<br><br>**Content on Epidemiology Score: 2**<br><br>**VIDEO TOTAL-CONTENT SCORE: 9**<br><br>**GQS: 2**
Link: https://www.youtube.com/watch?v=A2JAgQCx3Dw<br><br>Lenght (min): 4.56<br><br>Time elapsed since upload (days): 2521<br><br>Target audience: Laypersons	Source: Other<br><br>**Video Source Reliability (JAMA): 2**	Views (n.748)<br>Likes (n.0)<br>Dislikes (n.0)<br>Comments (disabled)<br>Subscriptions (n.2430)<br><br>Like Ratio: 0<br>View Ratio: 29.67<br><br>**VPI: 0**	Information flow (3)<br>Information accuracy (3)<br>Quality (2)<br>Sensitivity (4)<br><br>**VIQI: 12**	Route of transmission (1)<br>HPV risk factors (1)<br>HPV screening (1)<br>Genotypes (1)<br>Oncogenic role (1)<br><br>Skin lesions (benign) (1)<br>Mucosal lesions (benign) (1)<br>Oral lesions (benign) (0)<br><br>Genital cancer Fx (1)<br>Genital cancer Mx (0)<br>OSCC (0)<br><br>**Content on HPV infection and related lesions Score: 8**	Age (1)<br>Gender (0)<br>Type of vaccines (0)<br>HPV vaccine safety (1)<br><br>HPV vaccination advice:<br>Encouraging (1)<br>Discouraging (0)<br>Neutral (0)<br><br>Fake news (0)<br><br>**Content on HPV vaccination Score: 3**	Vaccination course (1)<br>Projections of genital cancer Fx (1)<br>Projections of genital cancer Mx (0)<br>Projection of OSCC (0)<br><br>**Content on Epidemiology Score: 2**<br><br>**VIDEO TOTAL-CONTENT SCORE: 13**<br><br>**GQS: 3**
Link: https://www.youtube.com/watch?v=eqoCHYRf9Os<br><br>Lenght (min): 11.25<br><br>Time elapsed since upload (days): 489	Source: Hospital/university	Views (n.242)<br>Likes (n.2)<br>Dislikes (n.0)<br>Comments (n.12)	Information flow (4)<br>Information accuracy (4)<br>Quality (3)	Route of transmission (1)<br>HPV risk factors (1)<br>HPV screening (1)<br>Genotypes (1)<br>Oncogenic role (1)	Age (1)<br>Gender (0)<br>Type of vaccines (0)<br>HPV vaccine safety (1)	Vaccination course (1)<br>Projections of genital cancer Fx (1)<br>Projections of genital cancer Mx (1)

Target audience: Both	**Video Source Reliability (JAMA): 3**	Subscriptions (n.4840)<br><br>Like Ratio: 0.82<br>View Ratio: 49.48<br><br>**VPI: 0.40**	Sensitivity (3)<br><br>**VIQI: 14**	Skin lesions (benign) (1)<br>Mucosal lesions (benign) (1)<br>Oral lesions (benign) (0)<br><br>Genital cancer Fx (1)<br>Genital cancer Mx (1)<br>OSCC (1)<br><br>**Content on HPV infection and related lesions Score: 10**	HPV vaccination advice:<br>Encouraging (1)<br>Discouraging (0)<br>Neutral (0)<br><br>Fake news (0)<br><br>**Content on HPV vaccination Score: 3**	Projection of OSCC (0)<br><br>**Content on Epidemiology Score: 3**<br><br>**VIDEO TOTAL-CONTENT SCORE: 16**<br><br>**GQS: 4**
Link: https://www.youtube.com/watch?app=desktop&v=SfDfCJHL9GM<br><br>Lenght (min): 7.51<br><br>Time elapsed since upload (days): 1626<br><br>Target audience: Laypersons	Source: Other<br><br>**Video Source Reliability (JAMA): 3**	Views (n.1716)<br>Likes (n.29)<br>Dislikes (n.3)<br>Comments (n.16)<br>Subscriptions (n.318000)<br><br>Like Ratio: 1.86<br>View Ratio: 105.53<br><br>**VPI: 1.96**	Information flow (2)<br>Information accuracy (2)<br>Quality (2)<br>Sensitivity (3)<br><br>**VIQI: 9**	Route of transmission (1)<br>HPV risk factors (1)<br>HPV screening (1)<br>Genotypes (0)<br>Oncogenic role (1)<br><br>Skin lesions (benign) (0)<br>Mucosal lesions (benign) (1)<br>Oral lesions (benign) (1)<br><br>Genital cancer Fx (1)<br>Genital cancer Mx (0)<br>OSCC (1)<br><br>**Content on HPV infection and related lesions Score: 8**	Age (1)<br>Gender (0)<br>Type of vaccines (0)<br>HPV vaccine safety (1)<br><br>HPV vaccination advice:<br>Encouraging (1)<br>Discouraging (0)<br>Neutral (0)<br><br>Fake news (0)<br><br>**Content on HPV vaccination Score: 3**	Vaccination course (1)<br>Projections of genital cancer Fx (1)<br>Projections of genital cancer Mx (0)<br>Projection of OSCC (0)<br><br>**Content on Epidemiology Score: 2**<br><br>**VIDEO TOTAL-CONTENT SCORE: 13**<br><br>**GQS: 2**
Link: https://www.youtube.com/watch?app=desktop&v=lJ99iMGYs7I<br><br>Lenght (min): 17.43<br><br>Time elapsed since upload (days): 1754<br><br>Target audience: Both	Source: Other<br><br>**Video Source Reliability (JAMA): 3**	Views (n.1594)<br>Likes (n.33)<br>Dislikes (n.6)<br>Comments (n.9)<br>Subscriptions (n.37300000)<br><br>Like Ratio: 2.44<br>View Ratio: 90.87<br><br>**VPI: 2.21**	Information flow (3)<br>Information accuracy (4)<br>Quality (4)<br>Sensitivity (3)<br><br>**VIQI: 14**	Route of transmission (1)<br>HPV risk factors (1)<br>HPV screening (1)<br>Genotypes (1)<br>Oncogenic role (1)<br><br>Skin lesions (benign) (0)<br>Mucosal lesions (benign) (1)<br>Oral lesions (benign) (0)<br><br>Genital cancer Fx (1)<br>Genital cancer Mx (1)	Age (1)<br>Gender (0)<br>Type of vaccines (0)<br>HPV vaccine safety (1)<br><br>HPV vaccination advice:<br>Encouraging (1)<br>Discouraging (0)<br>Neutral (0)<br><br>Fake news (0)	Vaccination course (1)<br>Projections of genital cancer Fx (1)<br>Projections of genital cancer Mx (0)<br>Projection of OSCC (0)<br><br>**Content on Epidemiology Score: 2**<br><br>**VIDEO TOTAL-CONTENT SCORE: 14**

				OSCC (1)<br><br>**Content on HPV infection and related lesions Score: 9**	**Content on HPV vaccination Score: 3**	**GQS: 3**
Link: https://www.youtube.com/watch?app=desktop&v=xXjsn9L-SGI<br><br>Lenght (min): 6.30<br><br>Time elapsed since upload (days): 2200<br><br>Target audience: Laypersons	Source: Commercial<br><br>**Video Source Reliability (JAMA): 1**	Views (n.695711)<br>Likes (n.2976)<br>Dislikes (n.746)<br>Comments (disabled)<br>Subscriptions (n.2270000)<br><br>Like Ratio: 0.53<br>View Ratio: 31623.22<br><br>**VPI: 167.50**	Information flow (1)<br>Information accuracy (1)<br>Quality (1)<br>Sensitivity (4)<br><br>**VIQI: 7**	Route of transmission (0)<br>HPV risk factors (0)<br>HPV screening (0)<br>Genotypes (0)<br>Oncogenic role (0)<br><br>Skin lesions (benign) (0)<br>Mucosal lesions (benign) (0)<br>Oral lesions (benign) (0)<br><br>Genital cancer Fx (0)<br>Genital cancer Mx (0)<br>OSCC (0)<br><br>**Content on HPV infection and related lesions Score: 0**	Age (0)<br>Gender (0)<br>Type of vaccines (0)<br>HPV vaccine safety (1)<br><br>HPV vaccination advice:<br>Encouraging (0)<br>Discouraging (1)<br>Neutral (0)<br><br>Fake news (1)<br><br>**Content on HPV vaccination Score: 3**	Vaccination course (0)<br>Projections of genital cancer Fx (0)<br>Projections of genital cancer Mx (0)<br>Projection of OSCC (0)<br><br>**Content on Epidemiology Score: 0**<br><br>**VIDEO TOTAL-CONTENT SCORE: 3**<br><br>**GQS: 1**
Link: https://www.youtube.com/watch?app=desktop&v=bg1NBTEhcU8<br><br>Lenght (min): 9.57<br><br>Time elapsed since upload (days): 4590<br><br>Target audience: Laypersons	Source: Commercial<br><br>**Video Source Reliability (JAMA): 1**	Views (n.33561)<br>Likes (n.794)<br>Dislikes (n.19)<br>Comments (n.251)<br>Subscriptions (n.3650000)<br><br>Like Ratio: 2.42<br>View Ratio: 731.17<br><br>**VPI: 17.69**	Information flow (1)<br>Information accuracy (1)<br>Quality (2)<br>Sensitivity (2)<br><br>**VIQI: 6**	Route of transmission (0)<br>HPV risk factors (0)<br>HPV screening (1)<br>Genotypes (1)<br>Oncogenic role (1)<br><br>Skin lesions (benign) (0)<br>Mucosal lesions (benign) (0)<br>Oral lesions (benign) (0)<br><br>Genital cancer Fx (0)<br>Genital cancer Mx (0)<br>OSCC (0)<br><br>**Content on HPV infection and related lesions Score: 3**	Age (0)<br>Gender (0)<br>Type of vaccines (1)<br>HPV vaccine safety (1)<br><br>HPV vaccination advice:<br>Encouraging (0)<br>Discouraging (1)<br>Neutral (0)<br><br>Fake news (1)<br><br>**Content on HPV vaccination Score: 4**	Vaccination course (0)<br>Projections of genital cancer Fx (0)<br>Projections of genital cancer Mx (0)<br>Projection of OSCC (0)<br><br>**Content on Epidemiology Score: 0**<br><br>**VIDEO TOTAL-CONTENT SCORE: 7**<br><br>**GQS: 1**
Link: https://www.youtube.com/watch?app=desktop&v=vD-M-6d8RJw	Source: Other	Views (n.88)<br>Likes (n.1)<br>Dislikes (n.0)	Information flow (3)	Route of transmission (1)<br>HPV risk factors (1)<br>HPV screening (1)	Age (1)<br>Gender (0)<br>Type of vaccines (0)	Vaccination course (0)<br>Projections of genital cancer Fx (1)

Lenght (min): 4.54<br><br>Time elapsed since upload (days): 4207<br><br>Target audience: Both	**Video Source Reliability (JAMA): 3**	Comments (n.0)<br>Subscriptions (n.34600)<br><br>Like Ratio: 1.13<br>View Ratio: 2.09<br><br>**VPI: 0.02**	Information accuracy (3)<br>Quality (2)<br>Sensitivity (4)<br><br>**VIQI: 12**	Genotypes (0)<br>Oncogenic role (1)<br><br>Skin lesions (benign) (0)<br>Mucosal lesions (benign) (0)<br>Oral lesions (benign) (0)<br><br>Genital cancer Fx (1)<br>Genital cancer Mx (1)<br>OSCC (1)<br><br>**Content on HPV infection and related lesions Score: 7**	HPV vaccine safety (1)<br><br>HPV vaccination advice:<br>Encouraging (1)<br>Discouraging (0)<br>Neutral (0)<br><br>Fake news (0)<br><br>**Content on HPV vaccination Score: 3**	Projections of genital cancer Mx (1)<br>Projection of OSCC (0)<br><br>**Content on Epidemiology Score: 2**<br><br>**VIDEO TOTAL-CONTENT SCORE: 12**<br><br>**GQS: 3**
Link: https://www.youtube.com/watch?app=desktop&v=tfsB8DwtIHE<br><br>Lenght (min): 11.02<br><br>Time elapsed since upload (days): 10<br><br>Target audience: Professional	Source: Other<br><br>**Video Source Reliability (JAMA): 3**	Views (n.8)<br>Likes (n.0)<br>Dislikes (n.0)<br>Comments (disabled)<br>Subscriptions (n.411)<br><br>Like Ratio: 0<br>View Ratio: 80<br><br>**VPI: 0**	Information flow (3)<br>Information accuracy (3)<br>Quality (4)<br>Sensitivity (2)<br><br>**VIQI: 12**	Route of transmission (1)<br>HPV risk factors (1)<br>HPV screening (1)<br>Genotypes (0)<br>Oncogenic role (1)<br><br>Skin lesions (benign) (0)<br>Mucosal lesions (benign) (0)<br>Oral lesions (benign) (0)<br><br>Genital cancer Fx (1)<br>Genital cancer Mx (0)<br>OSCC (0)<br><br>**Content on HPV infection and related lesions Score: 5**	Age (1)<br>Gender (0)<br>Type of vaccines (1)<br>HPV vaccine safety (1)<br><br>HPV vaccination advice:<br>Encouraging (1)<br>Discouraging (0)<br>Neutral (0)<br><br>Fake news (0)<br><br>**Content on HPV vaccination Score: 4**	Vaccination course (1)<br>Projections of genital cancer Fx (1)<br>Projections of genital cancer Mx (0)<br>Projection of OSCC (0)<br><br>**Content on Epidemiology Score: 2**<br><br>**VIDEO TOTAL-CONTENT SCORE: 11**<br><br>**GQS: 3**
Link: https://www.youtube.com/watch?app=desktop&v=iGQBQPqT6os<br><br>Lenght (min): 10.39<br><br>Time elapsed since upload (days): 1898<br><br>Target audience: Laypersons	Source: Other<br><br>**Video Source Reliability (JAMA): 3**	Views (n.54982)<br>Likes (n.2891)<br>Dislikes (n.77)<br>Comments (n.535)<br>Subscriptions (n.1000060)<br><br>Like Ratio: 5.39<br>View Ratio: 2896.83<br><br>**VPI: 156.13**	Information flow (4)<br>Information accuracy (3)<br>Quality (1)<br>Sensitivity (3)<br><br>**VIQI: 11**	Route of transmission (1)<br>HPV risk factors (1)<br>HPV screening (0)<br>Genotypes (0)<br>Oncogenic role (1)<br><br>Skin lesions (benign) (0)<br>Mucosal lesions (benign) (1)<br>Oral lesions (benign) (0)	Age (1)<br>Gender (0)<br>Type of vaccines (0)<br>HPV vaccine safety (1)<br><br>HPV vaccination advice:<br>Encouraging (1)<br>Discouraging (0)<br>Neutral (0)	Vaccination course (0)<br>Projections of genital cancer Fx (0)<br>Projections of genital cancer Mx (0)<br>Projection of OSCC (0)<br><br>**Content on Epidemiology Score: 0**

				Genital cancer Fx (1)<br>Genital cancer Mx (1)<br>OSCC (1)<br><br>**Content on HPV infection and related lesions Score: 7**	Fake news (0)<br><br>**Content on HPV vaccination Score:3**	**VIDEO TOTAL-CONTENT SCORE: 10**<br><br>**GQS: 3**
Link: https://www.youtube.com/watch?app=desktop&v=4aZYMOR5GqE<br><br>Lenght (min): 4.02<br><br>Time elapsed since upload (days): 1236<br><br>Target audience: Laypersons	Source: Hosital/university<br><br>**Video Source Reliability (JAMA): 3**	Views (n.144)<br>Likes (disabled)<br>Dislikes (disabled)<br>Comments (disabled)<br>Subscriptions (n.19900)<br><br>Like Ratio: Not evaluable<br>View Ratio: 11.65<br><br>**VPI: Not evaluable**	Information flow (3)<br>Information accuracy (3)<br>Quality (1)<br>Sensitivity (3)<br><br>**VIQI: 10**	Route of transmission (1)<br>HPV risk factors (1)<br>HPV screening (0)<br>Genotypes (1)<br>Oncogenic role (1)<br><br>Skin lesions (benign) (0)<br>Mucosal lesions (benign) (0)<br>Oral lesions (benign) (0)<br><br>Genital cancer Fx (1)<br>Genital cancer Mx (1)<br>OSCC (1)<br><br>**Content on HPV infection and related lesions Score: 7**	Age (1)<br>Gender (0)<br>Type of vaccines (0)<br>HPV vaccine safety (1)<br><br>HPV vaccination advice:<br>Encouraging (1)<br>Discouraging (0)<br>Neutral (0)<br><br>Fake news (0)<br><br>**Content on HPV vaccination Score: 3**	Vaccination course (1)<br>Projections of genital cancer Fx (0)<br>Projections of genital cancer Mx (0)<br>Projection of OSCC (0)<br><br>**Content on Epidemiology Score: 1**<br><br>**VIDEO TOTAL-CONTENT SCORE: 11**<br><br>**GQS: 3**
Link: https://www.youtube.com/watch?app=desktop&v=O61uiPqvSZ8<br><br>Lenght (min): 9.27<br><br>Time elapsed since upload (days): 1553<br><br>Target audience: Laypersons	Source: Other<br><br>**Video Source Reliability (JAMA): 3**	Views (n.49)<br>Likes (n.0)<br>Dislikes (n.0)<br>Comments (disabled)<br>Subscriptions (617)<br><br>Like Ratio: 0<br>View Ratio: 3.15<br><br>**VPI: 0**	Information flow (3)<br>Information accuracy (4)<br>Quality (4)<br>Sensitivity (3)<br><br>**VIQI: 14**	Route of transmission (1)<br>HPV risk factors (1)<br>HPV screening (1)<br>Genotypes (1)<br>Oncogenic role (1)<br><br>Skin lesions (benign) (0)<br>Mucosal lesions (benign) (0)<br>Oral lesions (benign) (0)<br><br>Genital cancer Fx (1)<br>Genital cancer Mx (1)<br>OSCC (1)<br><br>**Content on HPV infection and related lesions Score: 8**	Age (1)<br>Gender (0)<br>Type of vaccines (0)<br>HPV vaccine safety (1)<br><br>HPV vaccination advice:<br>Encouraging (1)<br>Discouraging (0)<br>Neutral (0)<br><br>Fake news (0)<br><br>**Content on HPV vaccination Score: 3**	Vaccination course (0)<br>Projections of genital cancer Fx (1)<br>Projections of genital cancer Mx (1)<br>Projection of OSCC (1)<br><br>**Content on Epidemiology Score: 3**<br><br>**VIDEO TOTAL-CONTENT SCORE: 14**<br><br>**GQS: 3**

Link:<br>https://www.youtube.com/watch?app=desktop&v=zYr7xDIq0P4<br><br>Lenght (min): 11.32<br><br>Time elapsed since upload (days): 1243<br><br>Target audience: Laypersons	Source: Commercial<br><br>**Video Source Reliability (JAMA): 3**	Views (n.412)<br>Likes (n.11)<br>Dislikes (n.7)<br>Comments (n.11)<br>Subscriptions (n.1000080)<br><br>Like Ratio: 4.36<br>View Ratio: 33.14<br><br>**VPI: 1.44**	Information flow (2)<br>Information accuracy (2)<br>Quality (1)<br>Sensitivity (4)<br><br>**VIQI: 9**	Route of transmission (1)<br>HPV risk factors (1)<br>HPV screening (1)<br>Genotypes (0)<br>Oncogenic role (1)<br><br>Skin lesions (benign) (0)<br>Mucosal lesions (benign) (0)<br>Oral lesions (benign) (0)<br><br>Genital cancer Fx (1)<br>Genital cancer Mx (1)<br>OSCC (0)<br><br>**Content on HPV infection and related lesions Score: 6**	Age (1)<br>Gender (0)<br>Type of vaccines (0)<br>HPV vaccine safety (1)<br><br>HPV vaccination advice:<br>Encouraging (1)<br>Discouraging (0)<br>Neutral (0)<br><br>Fake news (0)<br><br>**Content on HPV vaccination Score: 3**	Vaccination course (0)<br>Projections of genital cancer Fx (0)<br>Projections of genital cancer Mx (0)<br>Projection of OSCC (0)<br><br>**Content on Epidemiology Score: 0**<br><br>**VIDEO TOTAL-CONTENT SCORE: 9**<br><br>**GQS: 2**
Link:<br>https://www.youtube.com/watch?app=desktop&v=_AEaLaypWMI<br><br>Lenght (min): 5.57<br><br>Time elapsed since upload (days): 3957<br><br>Target audience: Laypersons	Source: Other<br><br>**Video Source Reliability (JAMA): 3**	Views (n.844)<br>Likes (n.2)<br>Dislikes (n.0)<br>Comments (n.0)<br>Subscriptions (n.10800)<br><br>Like Ratio: 0.23<br>View Ratio: 21.32<br><br>**VPI: 0.04**	Information flow (3)<br>Information accuracy (3)<br>Quality (4)<br>Sensitivity (3)<br><br>**VIQI: 13**	Route of transmission (1)<br>HPV risk factors (1)<br>HPV screening (1)<br>Genotypes (1)<br>Oncogenic role (1)<br><br>Skin lesions (benign) (0)<br>Mucosal lesions (benign) (0)<br>Oral lesions (benign) (0)<br><br>Genital cancer Fx (1)<br>Genital cancer Mx (1)<br>OSCC (1)<br><br>**Content on HPV infection and related lesions Score: 8**	Age (1)<br>Gender (0)<br>Type of vaccines (0)<br>HPV vaccine safety (1)<br><br>HPV vaccination advice:<br>Encouraging (1)<br>Discouraging (0)<br>Neutral (0)<br><br>Fake news (0)<br><br>**Content on HPV vaccination Score: 3**	Vaccination course (1)<br>Projections of genital cancer Fx (0)<br>Projections of genital cancer Mx (0)<br>Projection of OSCC (0)<br><br>**Content on Epidemiology Score: 1**<br><br>**VIDEO TOTAL-CONTENT SCORE: 12**<br><br>**GQS: 3**
Link:<br>https://www.youtube.com/watch?app=desktop&v=MPJrKrrXXEU<br><br>Lenght (min): 4.52<br><br>Time elapsed since upload (days): 1706<br><br>Target audience: Laypersons	Source: Other<br><br>**Video Source Reliability (JAMA): 2**	Views (n.659)<br>Likes (n.4)<br>Dislikes (n.0)<br>Comments (n.2)<br>Subscriptions (n.53200)<br><br>Like Ratio: 0.60	Information flow (3)<br>Information accuracy (3)<br>Quality (3)<br>Sensitivity (3)<br><br>**VIQI:** 12	Route of transmission (0)<br>HPV risk factors (0)<br>HPV screening (0)<br>Genotypes (1)<br>Oncogenic role (1)<br><br>Skin lesions (benign) (0)	Age (1)<br>Gender (0)<br>Type of vaccines (0)<br>HPV vaccine safety (1)<br><br>HPV vaccination advice:<br>Encouraging (1)<br>Discouraging (0)	Vaccination course (0)<br>Projections of genital cancer Fx (0)<br>Projections of genital cancer Mx (0)<br>Projection of OSCC (0)

		View Ratio: 38.63<br><br>**VPI: 0.23**		Mucosal lesions (benign) (0)<br>Oral lesions (benign) (0)<br><br>Genital cancer Fx (1)<br>Genital cancer Mx (1)<br>OSCC (0)<br><br>**Content on HPV infection and related lesions Score: 4**	Neutral (0)<br><br>Fake news (0)<br><br>**Content on HPV vaccination Score: 3**	**Content on Epidemiology Score: 0**<br><br>**VIDEO TOTAL-CONTENT SCORE: 7**<br><br>**GQS: 3**
Link: https://www.youtube.com/watch?app=desktop&v=IjX1p1EfzC0<br><br>Lenght (min): 5.05<br><br>Time elapsed since upload (days): 1450<br><br>Target audience: Laypersons	Source: Commercial<br><br>**Video Source Reliability (JAMA): 2**	Views (n.5)<br>Likes (n.0)<br>Dislikes (n.0)<br>Comments (n.0)<br>Subscriptions (n.10100)<br><br>Like Ratio: 0<br>View Ratio: 0.34<br><br>**VPI: 0**	Information flow (3)<br>Information accuracy (2)<br>Quality (2)<br>Sensitivity (2)<br><br>**VIQI: 9**	Route of transmission (1)<br>HPV risk factors (1)<br>HPV screening (1)<br>Genotypes (0)<br>Oncogenic role (1)<br><br>Skin lesions (benign) (0)<br>Mucosal lesions (benign) (0)<br>Oral lesions (benign) (0)<br><br>Genital cancer Fx (1)<br>Genital cancer Mx (0)<br>OSCC (0)<br><br>**Content on HPV infection and related lesions Score: 5**	Age (1)<br>Gender (0)<br>Type of vaccines (0)<br>HPV vaccine safety (1)<br><br>HPV vaccination advice:<br>Encouraging (1)<br>Discouraging (0)<br>Neutral (0)<br><br>Fake news (0)<br><br>**Content on HPV vaccination Score: 3**	Vaccination course (1)<br>Projections of genital cancer Fx (0)<br>Projections of genital cancer Mx (0)<br>Projection of OSCC (0)<br><br>**Content on Epidemiology Score: 1**<br><br>**VIDEO TOTAL-CONTENT SCORE: 9**<br><br>**GQS: 2**
Link: https://www.youtube.com/watch?app=desktop&v=DQQFskRbTOI<br><br>Lenght (min): 16.19<br><br>Time elapsed since upload (days): 239<br><br>Target audience: Professional	Source: Other<br><br>**Video Source Reliability (JAMA): 4**	Views (n.150)<br>Likes (n.3)<br>Dislikes (n.0)<br>Comments (n.0)<br>Subscriptions (n.200000)<br><br>Like Ratio: 2<br>View Ratio: 62.76<br><br>**VPI: 1.25**	Information flow (4)<br>Information accuracy (5)<br>Quality (5)<br>Sensitivity (3)<br><br>**VIQI: 17**	Route of transmission (1)<br>HPV risk factors (0)<br>HPV screening (1)<br>Genotypes (0)<br>Oncogenic role (1)<br><br>Skin lesions (benign) (0)<br>Mucosal lesions (benign) (0)<br>Oral lesions (benign) (0)<br><br>Genital cancer Fx (1)<br>Genital cancer Mx (1)<br>OSCC (1)	Age (1)<br>Gender (0)<br>Type of vaccines (0)<br>HPV vaccine safety (0)<br><br>HPV vaccination advice:<br>Encouraging (1)<br>Discouraging (0)<br>Neutral (0)<br><br>Fake news (0)<br><br>**Content on HPV vaccination Score: 2**	Vaccination course (1)<br>Projections of genital cancer Fx (0)<br>Projections of genital cancer Mx (0)<br>Projection of OSCC (0)<br><br>**Content on Epidemiology Score: 1**<br><br>**VIDEO TOTAL-CONTENT SCORE: 9**<br><br>**GQS: 2**

Content on HPV infection and related lesions Score: 6

Abbreviations: Human PapillomaVirus, “HPV”; minutes, “min”; Journal of American Medical Association, “JAMA”; number, “n.”; Video Power Index, “VPI”; Video Information and Quality Index, “VIQI”; Oral Squamous Cell Carcinoma, “OSCC”; General Quality Score, “GQS”. Characteristics: link; length (minutes); time elapsed since upload (days); target audience (layperson; professional; both). Source: dental care provider; other healthcare providers (any) and specialist; hospital/university; pharmaceutical industries; commercial; other; Video Source Reliability based on JAMA benchmark (0-4 total score). Popularity: views (n.); likes (n.); dislikes (n.); comments (n.); subscriptions (n.); like ratio; view ratio; Video Power Index “VPI”. Information and quality: information flow (1-5 score); information accuracy (1-5 score); quality (1-5 score); sensitivity (1-5 score); Video Information and Quality Index “VIQI” (1-20 total score). Content on HPV infection and related lesions: route of transmission (yes=1/no=0); HPV risk factors (yes=1/no=0); HPV screening (yes=1/no=0); genotypes (yes=1/no=0); oncogenic role (yes=1/no=0); skin lesions (benign) (yes=1/no=0); mucosal lesions (benign) (yes=1/no=0); oral lesions (benign) (yes=1/no=0); female genital cancer (yes=1/no=0); male genital cancer (yes=1/no=0); OSCC (yes=1/no=0); Content on HPV infection and related lesions Score (0-11). Content on HPV vaccine: age (yes=1/no=0); gender (yes=1/no=0); type of vaccines (yes=1/no=0); HPV vaccine safety (yes=1/no=0); HPV vaccination advice (encouraging/discouraging/neutral) (yes=1/no=0); fake news (yes=1/no=0); Content HPV vaccine Score (1-8). Content on the epidemiology of HPV vaccination and HPV-related cancers: vaccination course (yes=1/no=0); projections of female genital cancer (yes=1/no=0); projections of male genital cancer (yes=1/no=0); projection of OSCC (yes=1/no=0); Content Epidemiology Score (0-4). Video Total-Content Score (1-23). Global Quality Scale “GQS”. Video Educational Value (very low/low for GQS 3<; medium/good/excellent for GQS ≥3).
